# Host-specific sensing of coronaviruses and picornaviruses by the CARD8 inflammasome

**DOI:** 10.1371/journal.pbio.3002144

**Published:** 2023-06-08

**Authors:** Brian V. Tsu, Rimjhim Agarwal, Nandan S. Gokhale, Jessie Kulsuptrakul, Andrew P. Ryan, Elizabeth J. Fay, Lennice K. Castro, Christopher Beierschmitt, Christina Yap, Elizabeth A. Turcotte, Sofia E. Delgado-Rodriguez, Russell E. Vance, Jennifer L. Hyde, Ram Savan, Patrick S. Mitchell, Matthew D. Daugherty

**Affiliations:** 1 Department of Molecular Biology, University of California, San Diego, La Jolla, California, United States of America; 2 Department of Immunology, University of Washington; Seattle, Washington, United States of America; 3 Molecular and Cellular Biology Graduate Program, University of Washington; Seattle, Washington, United States of America; 4 Department of Microbiology, University of Washington; Seattle, Washington, United States of America; 5 Division of Immunology and Pathogenesis, University of California, Berkeley, Berkeley, California, United States of America; 6 Howard Hughes Medical Institute, University of California, Berkeley, Berkeley, California, United States of America; New York University School of Medicine, UNITED STATES

## Abstract

Hosts have evolved diverse strategies to respond to microbial infections, including the detection of pathogen-encoded proteases by inflammasome-forming sensors such as NLRP1 and CARD8. Here, we find that the 3CL protease (3CL^pro^) encoded by diverse coronaviruses, including Severe Acute Respiratory Syndrome Coronavirus 2 (SARS-CoV-2), cleaves a rapidly evolving region of human CARD8 and activates a robust inflammasome response. CARD8 is required for cell death and the release of pro-inflammatory cytokines during SARS-CoV-2 infection. We further find that natural variation alters CARD8 sensing of 3CL^pro^, including 3CL^pro^-mediated antagonism rather than activation of megabat CARD8. Likewise, we find that a single nucleotide polymorphism (SNP) in humans reduces CARD8’s ability to sense coronavirus 3CL^pros^ and, instead, enables sensing of 3C proteases (3C^pro^) from select picornaviruses. Our findings demonstrate that CARD8 is a broad sensor of viral protease activities and suggests that CARD8 diversity contributes to inter- and intraspecies variation in inflammasome-mediated viral sensing and immunopathology.

## Abstract 2

This study describes an innate immune mechanism for sensing coronavirus and picornavirus infection by detecting the activity of virus-encoded proteases. Genetic variants among mammalian hosts affect which viruses can be sensed by this host ‘tripwire’ system, confirming its importance in the evolutionary arms race between viruses and the host immune response.

## Introduction

Effector-triggered immunity (ETI) is a host defense strategy by which innate immune sensors recognize pathogens via the detection of pathogen-specific activities [1–7]. Long recognized as an important strategy of host defense in plants, ETI has only recently been considered to have a role in vertebrate pathogen recognition [1–3,7]. In particular, a subset of inflammasome-forming innate immune sensors detect pathogen-specific activities. Inflammasomes are large, intracellular immune complexes that coordinate downstream inflammatory responses. Upon pathogen detection, inflammasome-forming sensors oligomerize into platforms for the recruitment and activation of pro-inflammatory caspases, predominantly caspase-1 (CASP1), to initiate inflammatory signaling via interleukin (IL)-1β and IL-18 and pyroptotic cell death through cleavage of the pore-forming protein Gasdermin D (GSDMD) [8–10].

In humans, the activity of viral proteases can be sensed by the inflammasome-forming sensors CARD8 and NLRP1 [11–15]. CARD8 comprises a disordered N-terminal region and a C-terminal function-to-find domain (FIIND) and caspase activation and recruitment domain (CARD). The FIIND undergoes self-cleavage resulting in a bipartite sensor, with the disordered N-terminus acting as a “tripwire” for viral proteases [16]. For instance, proteolytic cleavage of CARD8 by the HIV-1 protease (HIV-1^pro^) leads to proteasome-dependent “functional degradation” [17,18] of the cleaved N-terminus and release of the bioactive CARD-containing C-terminus, which is sufficient for inflammasome assembly and activation [15]. However, the extent to which CARD8 has evolved to sense other viral proteases and function as an innate immune sensor of viral infection has been unclear.

Here, we find that CARD8 is a dynamically evolving ETI sensor of a diverse range of viral proteases and is an important component of cellular sensing of coronavirus infection. First, we demonstrate that site-specific cleavage of CARD8 by the coronavirus 3C-like protease (3CL^pro^) activates the CARD8 inflammasome and that infection with Severe Acute Respiratory Syndrome Coronavirus 2 (SARS-CoV-2) or the seasonal coronavirus, hCoV-229E, leads to CARD8-dependent cell death and pro-inflammatory cytokine release. Our evolutionary and functional studies further indicate that CARD8 sequence variation between species and within humans has a profound impact on the ability to sense viral proteases. For instance, we find that bats have either lost CARD8 or have a CARD8 protein that is antagonized, rather than activated, by coronavirus 3CL^pro^ cleavage. Furthermore, we find that a high-frequency human single nucleotide polymorphism (SNP) within the 3CL^pro^ cleavage site attenuates the ability of CARD8 to sense coronavirus 3CL^pro^. Strikingly, we find that this same human SNP potentiates the ability of CARD8 to sense the 3C proteases (3C^pros^) from a subset of human picornaviruses, suggestive of an evolutionary trade-off in CARD8 that may shape individual responses to viral infection. In total, our work reveals the broad capacity of CARD8 to sense a diverse set of coronavirus 3CL and picornaviral 3C^pros^ and promote an inflammasome response in a manner that is exquisitely host and virus specific.

### The SARS-CoV-2 3CL^pro^ activates the human CARD8 inflammasome via proteolysis within the disordered N-terminus

**Fig 1:**
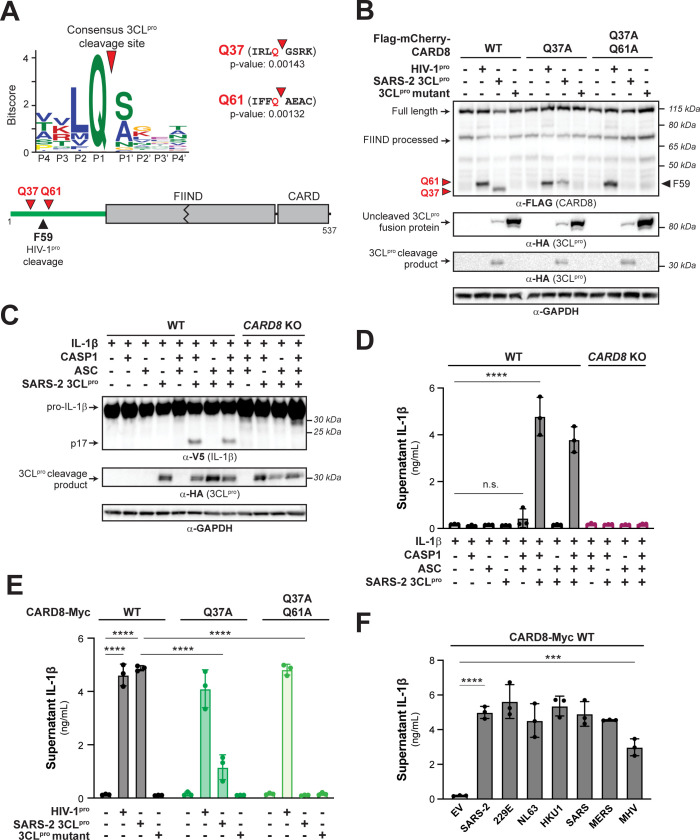
3CL^pro^ from SARS-CoV-2 and other coronaviruses cleaves and activates the human CARD8 inflammasome. (A) A consensus Beta-CoV 3CL^pro^ cleavage motif (upper panel; S1 Fig and Materials and methods) was used to predict two 3CL^pro^ cleavage sites (red triangles) within the disordered “tripwire” N-terminus of human CARD8 (lower panel, green) near the described site of HIV-1^pro^ cleavage (black triangle). Flanking residues and *p*-values of prediction for each site (Q37 and Q61) are shown. (B) HEK293T cells were transfected with the indicated CARD8 construct in the presence (“+”) or absence (“−”) of indicated proteases. Active (SARS-2 3CL^pro^) or catalytically inactive (3CL^pro^ mutant) protease from SARS-CoV-2 was expressed as an HA-tagged fusion construct (S2 Fig). HIV-1^pro^ was expressed from an untagged gag-pol construct. Triangles are as described in (A). (C, D) WT or *CARD8* KO HEK293T cells were transfected with (“+”) or without (“−”) indicated constructs. Inflammasome activation was monitored by immunoblotting for mature IL-1β (p17) (C) or measuring culture supernatant levels of bioactive IL-1β using IL1R-expressing reporter cells (D). (E, F) *CARD8* KO HEK293T cells were cotransfected with the indicated CARD8 and protease constructs and supernatant levels of bioactive IL-1β were measured by IL1R reporter assay. 3CL^pros^ from the following viruses were used: hCoV-229E (229E), hCoV-NL63 (NL63), hCoV-HKU1 (HKU1), SARS-CoV (SARS), MERS-CoV (MERS), and mouse hepatitis virus (MHV). (D-F) Individual values (*n =* 3), averages, and standard deviations shown are representative of experiments performed in triplicate. Data were analyzed using two-way ANOVA with Šidák’s post-test (D, E) or one-way ANOVA with Tukey’s post-test (F). *** = *p* < 0.001, **** = *p* < 0.0001, n.s. = not significant. Data for Fig 1A, 1D, 1E, and 1F can be found in S1 Data. Beta-CoV, betacoronavirus; HIV-1^pro^, HIV-1 protease; IL, interleukin; MHV, mouse hepatitis virus; SARS-CoV-2, Severe Acute Respiratory Syndrome Coronavirus 2; WT, wild-type; 3CL^pro^, 3CL protease.

Based on prior work showing that HIV-1^pro^ could activate CARD8 [15], we reasoned that other viral proteases might activate the CARD8 inflammasome. To predict if the CARD8 inflammasome can sense coronavirus infection by mimicking sites of viral polyprotein cleavage, we adapted our previous bioinformatic approach [14] to generate a predictive model for the cleavage specificity of *Coronaviridae* 3CL^pro^ (also known as main protease (M^pro^)), which has been shown to cleave host proteins in addition to the coronavirus polyprotein [16,19–22]. The resulting 3CL^pro^ cleavage motif, XXΦQ[G/A/S]XXX (where Φ denotes a hydrophobic residue and X denotes any amino acid) (**Figs 1A and S**1 **and S1 and S2 Tables**), is broadly consistent with previous studies [23–26] and allowed us to predict 2 putative 3CL^pro^ cleavage sites within the N-terminus of human CARD8 (**Fig 1A**).

To determine if human CARD8 is cleaved by coronavirus 3CL^pro^, we coexpressed an N-terminal 3xFlag/mCherry-tagged isoform of human CARD8 (wild-type (WT)) with 3CL^pro^ (encoded by nsp5) from SARS-CoV-2 in HEK293T cells (**Figs 1B and S2**). We used HIV-1^pro^ as a positive control since it had previously been shown to cleave CARD8 between residues F59-F60 (site F59 in **Fig 1A and 1B**). We observed an approximately 34 kDa CARD8 product in the presence of SARS-CoV-2 3CL^pro^ but not the catalytically inactive C145A 3CL^pro^ mutant. The approximately 34 kDa product is predicted to result from cleavage at site Q37 (**Fig 1A**), which migrated slightly below the cleavage product of HIV-1^pro^. Mutating the putative P1 residue in the Q37 site (CARD8 Q37A) eliminated the 34 kDa product, confirming SARS-CoV-2 3CL^pro^ cleavage at this site. The CARD8 Q37A mutant also revealed a cryptic approximately 37 kDa 3CL^pro^-dependent product, which matches cleavage at the predicted site Q61 (**Figs 1A and S2**). The CARD8 Q37A Q61A mutant was completely insensitive to cleavage by SARS-CoV-2 3CL^pro^ (**Fig 1B**), whereas cleavage by the HIV-1^pro^ was unperturbed by either mutant. Thus, CARD8 can be cleaved by SARS-CoV-2 3CL^pro^ at amino acid sequences that mimic the coronavirus polyprotein cleavage site.

For both NLRP1 and CARD8, N-terminal proteolytic cleavage can activate CASP1 in a reconstituted inflammasome assay [13–15,18,27]. Consistent with the prior observation that CARD8 is endogenously expressed in some HEK293T cell lines [15], transfection of our HEK293T cells with only CASP1, pro-IL-1β, and SARS-CoV-2 3CL^pro^ resulted in robust CASP1-dependent processing of pro-IL-1β to mature bioactive IL-1β (p17) as measured by immunoblot or IL-1β reporter assay (see **Materials and methods**) (**Fig 1C and 1D**). Importantly, 3CL^pro^-mediated inflammasome activation occurred independently of ASC, which is required for NLRP3 and NLRP1 inflammasome activation, but not CARD8 inflammasome activation [28–30]. We further confirmed a requirement for CARD8 by showing that there was no inflammasome activation when 3CL^pro^ was expressed in *CARD8* knockout (KO) cells (**Fig 1D**).

To confirm that SARS-CoV-2 3CL^pro^ cleavage of CARD8 is responsible for inflammasome activation, we complemented *CARD8* KO cells with WT CARD8 or cleavage site mutants. Complementation with WT CARD8 rescued both HIV-1^pro^- and 3CL^pro^-induced inflammasome activation (**Figs 1E and S3**). In contrast, whereas CARD8 3CL^pro^ cleavage site mutants had no effect on HIV-1^pro^-induced inflammasome activation, 3CL^pro^-induced inflammasome activation was reduced or abolished in *CARD8* KO cells complemented with CARD8 Q37A or CARD8 Q37A Q61A, respectively (**Figs 1E and S3)**. We observed that 3CL^pro^-induced inflammasome activation in *CARD8* KO cells complemented with CARD8 Q61A, however, was not significantly different compared to cells complemented with WT CARD8 (**S4 Fig**). These results validate that 3CL^pro^ site-specific cleavage at site Q37 but not Q61 is required for CARD8 inflammasome activation. As expected, 3CL^pro^ inflammasome activation did not occur in *CARD8* KO cells complemented with the CARD8 FIIND autoprocessing mutant (S297A) (**S5 Fig**) [15,31,32]. Taken together, our results indicate that human CARD8 senses the proteolytic activity of the SARS-CoV-2 3CL^pro^, which drives inflammasome activation via functional degradation.

To test if proteases from other coronaviruses also cleave CARD8, we cloned 3CL^pros^ from other human-relevant betacoronaviruses (Beta-CoVs) (SARS-CoV (SARS), MERS-CoV (MERS), hCoV-HKU1 (HKU1)), 2 human Alpha-CoVs (hCoV-229E (229E) and hCoV-NL63 (NL63)), and the mouse Beta-CoV murine hepatitis virus (MHV) (**S6 Fig**). Consistent with their structural and cleavage motif similarity [33], we found that every tested 3CL^pro^ was able to cleave and activate CARD8 in a site-specific manner (**Figs 1F and S7**). Thus, ETI by human CARD8 takes advantage of the evolutionary constraint on viral protease cleavage specificity to sense both endemic and pandemic human coronaviruses.

### Coronavirus infection activates the CARD8 inflammasome

**Fig 2:**
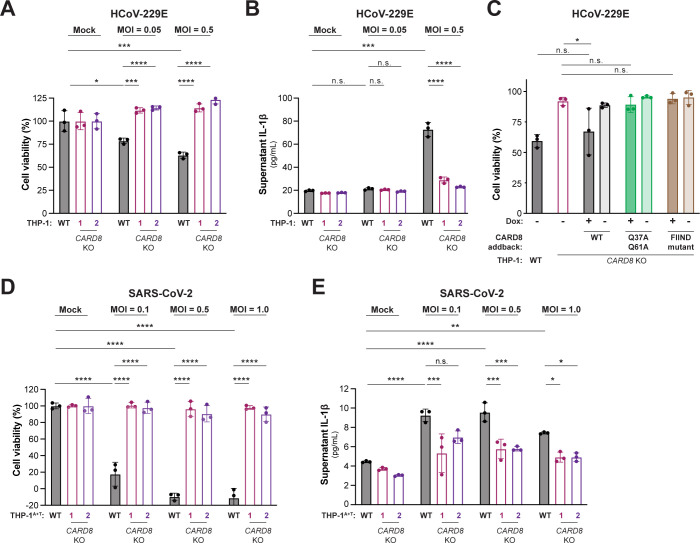
Coronavirus infection activates the CARD8 inflammasome in THP-1 cells. WT, *CARD8* KO1, and *CARD8* KO2 THP-1 cells (A, B), or THP-1 cells expressing ACE2 and TMPRSS2 (THP-1^T+A^) (D, E) were primed with 0.5 μg/mL Pam3CSK4 for 6 h, followed by infection with the coronaviruses hCoV-229E (A, B) or SARS-CoV-2 (SARS-2) (D, E) at the indicated MOI. (C) *CARD8* KO1 THP-1 cells were complemented with a using a Dox-inducible lentiviral construct expressing the indicated CARD8 variant. WT, *CARD8* KO1, or complemented cell lines were treated with Dox as indicated and infected with 200 PFUs hCoV-229E. After 48 h, cell viability (A, C, D) was measured using the Cell Titer Glo assay and IL-1β levels were measured using the IL1R reporter assay (B, E) as in **Fig 1D**. Data were analyzed using two-way ANOVA with Šidák’s post-test (A, B, D, E) or one-way ANOVA with Tukey’s post-test (C). * = *p* < 0.05, ** = *p* < 0.01, *** = *p* < 0.001, **** = *p* < 0.0001, n.s. = not significant. Data for Fig 2A, 2B, 2C, 2D and 2E can be found in S1 Data. Dox, doxycycline; IL, interleukin; MOI, multiplicity of infection; PFU, plaque-forming unit; SARS-CoV-2, Severe Acute Respiratory Syndrome Coronavirus 2; WT, wild-type.

We next evaluated the consequences of CARD8 inflammasome activation by infecting the human monocyte-like cell line THP-1 with hCoV-229E. We found that hCoV-229E-infected WT but not 2 independently derived *CARD8* KO THP-1 cell lines underwent significant cell death (**Fig 2A**) and release of IL-1β (**Fig 2B**), a result similar to treatment with Val-boroPro (VbP) (**S8 Fig**), which specifically activates the CARD8 inflammasome in myeloid and lymphoid lineages [32,34]. Virus-induced cell death and VbP-induced cell death were both dependent on CASP1, validating the involvement of the inflammasome for viral sensing (**S9 Fig**).

To further confirm that specific cleavage of CARD8 by 3CL^pro^ during viral infection is required for inflammasome-mediated sensing and cell death, we complemented *CARD8* KO THP-1 cells with either WT CARD8, cleavage resistant CARD8 (Q37A Q61A) or FIIND mutant CARD8 (S297A). As expected, complementation of *CARD8* KO cells with WT or cleavage-resistant CARD8 restored responsiveness to VbP, whereas complementation with FIIND mutant CARD8 did not (**S10 Fig**). We next infected these cell lines with hCoV-229E and compared cell viability of the complemented lines to the parental THP-1 and *CARD8 KO* THP-1 cells. Importantly, we observed virus-induced cell death when we complemented cells with WT CARD8 but not the cleavage resistant CARD8 or FIIND mutant CARD8 (**Figs 2C and S11**). These results validate that CARD8 senses coronavirus infection through protease-mediated cleavage of the CARD8 tripwire.

Recent evidence indicates that SARS-CoV-2 infection [35,36] or uptake via FcγR-mediated antibody-dependent enhancement [37] by monocytes or macrophages induces inflammasome activation. To determine if CARD8 senses and responds to SARS-CoV-2 infection in THP-1 cells, we engineered WT or *CARD8* KO THP-1 cells to express ACE2 and TMPRSS2 (THP-1^A+T^). As with hCoV-229E, we found that SARS-CoV-2 infection of THP-1^A+T^ WT but not *CARD8* KO cells induced both cell death and IL-1β release (**Fig 2D and 2E**). Together, our results demonstrate that CARD8 is a bona fide innate immune sensor of coronavirus infection via the detection of the enzymatic activity of viral 3CL^pro^.

### Inter- and intrahost diversity in CARD8 impacts inflammatory responses to coronavirus proteases

Host–virus interactions, including those between viral proteases and host cleavage targets, are often engaged in evolutionary arms races that shape the species specificity of these interactions [16,38–40]. Indeed, we have previously shown that the CARD8 homolog and viral protease sensor, NLRP1, has been duplicated and recurrently lost across mammalian evolution. *NLRP1* also displays strong signatures of positive selection in an N-terminal region of the protein that is cleaved by pathogen-encoded proteases, which we refer to as the “tripwire” region due to its role in host-specific virus detection and subsequent inflammasome activation [14,27]. We thus predicted that *CARD8* may have a similarly dynamic evolutionary history and that host inter- and intraspecies variation would underlie differences in CARD8 cleavage and inflammasome activation by coronavirus 3CL^pros^.

**Fig 3:**
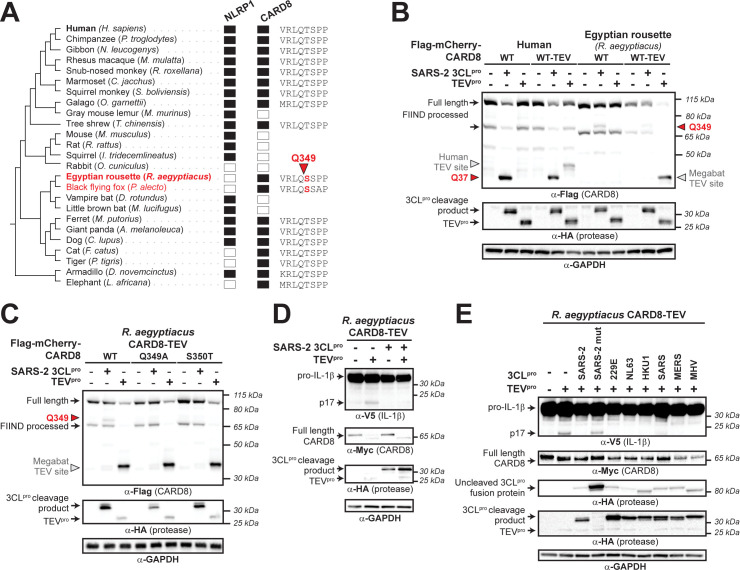
Megabat CARD8 is antagonized rather than activated by coronavirus 3CL^pro^. (A) Presence (filled rectangle) or absence (empty rectangle) of predicted *NLRP1* or *CARD8* orthologs in the indicated mammalian species. To the left is a species phylogeny. Megabat species are indicated in red. To the right is an alignment of a predicted 3CL^pro^ cleavage site in *Rousettus aegyptiacus* CARD8 (red triangle indicates site and number indicates residue position). (B) Human CARD8 or *R*. *aegyptiacus* CARD8 was cotransfected with either SARS-CoV-2 (SARS-2) 3CL^pro^ or protease from tobacco etch virus (TEV^pro^). For human or *R*. *aegyptiacus* CARD8 constructs labeled “WT-TEV,” a TEV^pro^ site was introduced into the N-terminus. The red triangles and amino acid number indicate the sites of 3CL^pro^ cleavage in human and *R*. *aegyptiacus* CARD8. The gray triangles indicate the sites of TEV protease cleavage within each CARD8 WT-TEV. (C) Mapping of the 3CL^pro^ site within *R*. *aegyptiacus* CARD8 was performed by transfecting the indicated point mutants with SARS-CoV-2 (SARS-2) 3CL^pro^ or TEV^pro^. 3CL^pro^ and TEV^pro^ sites are marked by triangles as in (B). (D) *CARD8* KO HEK293T cells were cotransfected with *R*. *aegyptiacus* IL-1β and CASP1, along with the indicated CARD8 and protease constructs. Presence of mature IL-1 β (p17) upon TEV^pro^ addition indicates successful reconstitution of the *R*. *aegyptiacus* CARD8 inflammasome, whereas absence of p17 upon SARS-2 3CL^pro^ indicates antagonism of the *R*. *aegyptiacus* CARD8 inflammasome. (C) *R*. *aegyptiacus* CARD8 inflammasome activation assays were performed as in (D) with the indicated 3CL^pro^ constructs. IL, interleukin; SARS-CoV-2, Severe Acute Respiratory Syndrome Coronavirus 2; TEV, tobacco etch virus; 3CL^pro^, 3CL protease.

We first found that both *CARD8* and *NLRP1* are each present in only certain mammalian lineages, consistent with their dynamic roles in host defense as opposed to dedicated housekeeping functions (**Fig 3A**). For instance, we found that within the order *Chiroptera* (bats), microbats retain a *NLRP1* ortholog but have lost *CARD8*, whereas megabats have lost *NLRP1* and only encode *CARD8*. Because bats serve as main reservoir hosts of emerging coronaviruses [41,42], but CARD8 is missing from microbats, we tested if the CARD8 inflammasome could serve as a sensor for 3CL^pro^ in the megabat species *Rousettus aegyptiacus*. Unlike human CARD8, the *R*. *aegyptiacus* CARD8 (CARD8_*Ra*_) N-terminus lacks sites Q37 and Q61 and was not cleaved by SARS-CoV-2 3CL^pro^. We did, however, observe a cleavage product that matched a predicted 3CL^pro^ site at Q349 in CARD8_*Ra*_ downstream of the FIIND autoprocessing site in the inflammasome-forming C-terminus (**Fig 3A and 3B**). Interestingly, megabats are the only mammals with a serine in the P1’ position of the cleavage site (**Figs 3A and S12)**, which is preferred for cleavage based on our computational model (**Fig 1A**). Indeed, a threonine in this position (S350T), which is found in human CARD8 as well as most other mammals (**S12 Fig**), prevents cleavage of CARD8_*Ra*_ by SARS-CoV-2 3CL^pro^ (**Fig 3C**). We next wished to test the functional effect of 3CL^pro^ on the megabat CARD8 inflammasome. First, we determined if functional degradation could activate megabat CARD8 by inserting a TEV^pro^ cleavage site into the N-terminus of CARD8_*Ra*_, permitting TEV^pro^ cleavage of CARD8_*Ra*_-TEV but not WT CARD8_*Ra*_. When coexpressed with CASP1 and pro-IL-1β from *R*. *aegyptiacus*, TEV^pro^ cleavage of CARD8_*Ra*_-TEV resulted in inflammasome activation, indicating that we can reconstitute the *R*. *aegyptiacus* CARD8 inflammasome in human cells (**Fig 3D**). Using this reconstitution system, we found that SARS-CoV-2 3CL^pro^ does not activate and, in fact, antagonizes TEV-mediated CARD8_*Ra*_ inflammasome activation (**Fig 3D)**, similar to our previous observations of viral proteases that antagonize the activation of the NLRP1 inflammasome [14]. We further found that all 3CL^pros^ that we tested can instead prevent TEV^pro^-mediated activation of the CARD8_*Ra*_ inflammasome (**Fig 3E**). Together with the loss of CARD8 from many bat species, these data provide a putative mechanism of disease tolerance that protects bats from immunopathogenic effects of inflammasome activation.

**Fig 4:**
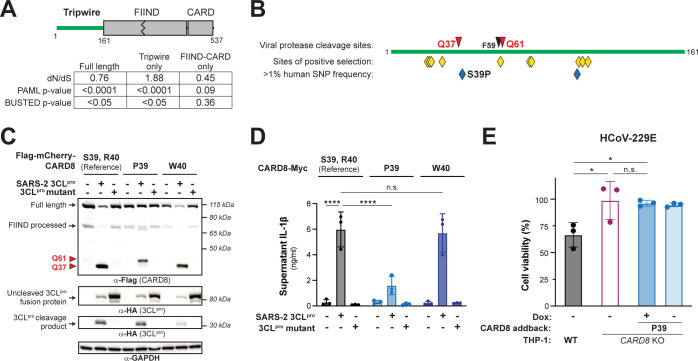
Human polymorphism in CARD8 reduces sensing of coronavirus 3CL^pro^. (A) Evolutionary analyses of positive selection were performed on full length *CARD8* (encoding residues 1–537), the disordered N-terminal “tripwire” region (encoding residues 1–161), and the FIIND-CARD region (encoding residues 162–537). *P* values from PAML and BUSTED analyses are shown, along with the dN/dS value obtained from PAML. (B) Schematic of the CARD8 “tripwire” region. Red and black triangles and amino acid numbers indicate sites of 3CL^pro^ and HIV-1^pro^ cleavage, respectively. Yellow diamonds indicate codons predicted to be evolving under positive selection by at least one evolutionary analysis (S4 Table). Blue diamonds indicate high frequency (>1% allele frequency) nonsynonymous SNPs in humans (S5 and S6 Tables). The position of the S39P substitution that results from SNP rs12463023 is shown. (C, D) Reference human CARD8 (S39, R40) or human CARD8 variants (P39 or W40) were coexpressed with the indicated protease construct and assayed for 3CL^pro^-mediated cleavage (C) or CARD8 inflammasome activation (D). (E) The CARD8 variant P39 was complemented into *CARD8* KO1 THP-1 cells using a Dox-inducible lentiviral construct and were infected along with WT and *CARD8* KO1 THP-1 cells with hCoV-229E in the presence or absence of 100 ng/mL Dox. Cell viability was measured using the Cell Titer Glo assay, 48 h post-infection. Individual values (*n =* 3), averages, and standard deviations shown are representative of experiments performed at least twice. Data were analyzed using two-way ANOVA with Šidák’s post-test (D) or one-way ANOVA with Tukey’s post-test (E). * = *p* < 0.05, **** = *p* < 0.0001, n.s. = not significant. Data for Fig 4D and 4E can be found in S1 Data. Dox, doxycycline; SNP, single nucleotide polymorphism; WT, wild-type; 3CL^pro^, 3CL protease.

We next focused on the evolution of human *CARD8*. Supporting a previous genome-wide study [43], we found evidence that *CARD8* has evolved under recurrent positive selection in hominoids and Old World monkeys, which we find is primarily driven by the N-terminal region of the protein (**Fig 4A**). Codon-based analyses also show that positively selected sites are predominantly found in the N-terminus, including a codon at position 60 that lies in the HIV-1^pro^ site and the secondary coronavirus 3CL^pro^ site (**Figs 4B and S13 and S5 Table)**. We thus infer that, like NLRP1, the CARD8 disordered N-terminus is a molecular “tripwire” that is rapidly evolving to mimic viral polyprotein sites and sense diverse viral proteases.

We further analyzed the human population for nonsynonymous SNPs in *CARD8* (**Fig 4B**). Within the N-terminus, we found several high frequency human SNPs, including a S39P variant that resides at the P2’ position within the 3CL^pro^ cleavage site and is present in >20% of all sampled African and African American individuals (GnomAD v3.1.2; [44]) (**Fig 4B and S5 and S6 Tables**). Strikingly, while CARD8 S39 and P39 variants are similarly cleaved and activated by HIV-1^pro^, the CARD8 P39 variant exhibits reduced sensitivity to cleavage and activation by coronavirus 3CL^pros^ (**Figs 4C, 4D and S14**). This is reinforced by our observation that a proline in the P2’ position is never found in the >10,000 polyprotein cleavage sites we sampled from Beta-CoVs (**Fig 1A and S1 Table**). In contrast, another human SNP (R40W) found in approximately 1 in every 2,000 alleles (**S5 Table**) does not detectably affect CARD8 cleavage in our assays (**Fig 4C and 4D**).

To determine if the CARD8 polymorphism at CARD8 residue 39 affects its sensing of coronaviruses, we complemented our *CARD8* KO THP-1 cells with the P39 CARD8 variant. We found that VbP responsiveness was restored when we complemented *CARD8* KO cells with CARD8 P39 (**S15 Fig)**, indicating that the P39 CARD8 variant is functional. However, unlike what we observed for CARD8 S39 (i.e., the reference allele) (**Fig 2C)**, we observed that CARD8 P39 did not restore responsiveness to hCoV-229E infection (**Fig 4E)**. These data suggest that polymorphism within the tripwire region of human CARD8 alters sensing and inflammasome responses to coronavirus infection.

### A human SNP confers a specificity switch for CARD8 sensing of coronavirus 3CL^pro^ and human rhinovirus 3C^pro^

**Fig 5:**
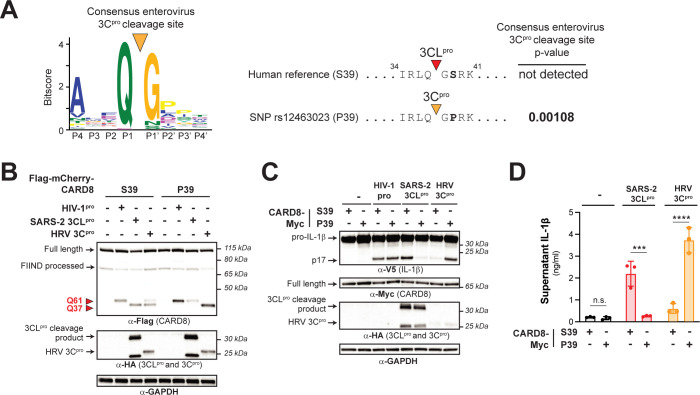
A human polymorphism dictates CARD8 sensing of coronavirus 3CL^pro^ and human rhinovirus 3C^pro^. (A) A previously generated 3C^pro^ consensus cleavage motif for enteroviruses (a genus within picornaviruses) [14], in which a proline is the most common amino acid found at the P2’ position, is shown. A 3C^pro^ cleavage site is predicted in the CARD8 P39 variant (encoded by rs12463023) but not the CARD8 S39 variant (encoded by the reference allele). (B-D). Human CARD8 S39 or CARD8 P39 were transfected with the indicated proteases and assayed for 3CL^pro^-mediated cleavage (B), CARD8 inflammasome-mediated maturation of IL-1β (C), or the release of bioactive IL-1β (D), as in **Fig 1**. Individual values (*n =* 3), averages, and standard deviations shown are representative of experiments performed in triplicate. Data were analyzed using two-way ANOVA with Šidák’s post-test. *** = *p* < 0.001, **** = *p* < 0.0001, n.s. = not significant. Data for Fig 5A and 5D can be found in S1 Data. HRV 3C^pro^, human rhinovirus 3C^pro^; IL, interleukin; 3C^pro^, 3C protease; 3CL^pro^, 3CL protease.

We next considered if the CARD8 P39 variant, in addition to affecting sensing of coronavirus 3CL^pros^, might also alter recognition of other viral pathogens. We noticed that although a P2’ proline is disfavored in our model for 3CL^pro^ cleavage, a P2’ proline is strongly preferred in our previously published model for cleavage site specificity for the 3C^pro^ from members of the enterovirus genus within the viral family *Picornaviridae* (**Fig 5A**) [14]. Indeed, our 3C^pro^ motif search [14] identified a cleavage site in CARD8 P39 but not CARD8 S39 (**Fig 5A**). Validating these bioinformatic predictions, cleavage assays with the 3C^pro^ from the respiratory enterovirus, human rhinovirus (HRV), revealed that HRV 3C^pro^ cleavage of human CARD8 at site Q37 is considerably more pronounced for the P39 variant than the S39 variant (**Fig 5B**). Likewise, we found that inflammasome activation by HRV 3C^pro^ was nearly absent in HEK293T *CARD8* KO cells complemented with CARD8 S39, whereas we observed robust inflammasome activation in cells complemented with CARD8 P39—the opposite sensitivity observed for SARS-CoV-2 3CL^pro^ (**Fig 5C and 5D**).

Finally, we wished to determine how human CARD8 sequence variation impacts cleavage by 3C^pros^ from a diverse range of picornaviruses. A recent paper found that infection with the enterovirus Coxsackievirus B3 (CVB3) could activate the inflammasome via cleavage of the S39 variant of CARD8 [11]. Although we do observe some cleavage of the CARD8 S39 variant by enterovirus 3C^pros^, we found that every tested enterovirus 3C^pro^ cleaves the CARD8 P39 with higher efficiency than the S39 variant, as we saw with HRV (**S16 Fig**). Also like HRV, we observe increased inflammasome activation when the P39 variant is tested against diverse enterovirus 3C^pros^. For instance, 3C^pro^ from either enterovirus D68 (EV68) or poliovirus (PV1) activates the CARD8 inflammasome when the P39 variant is used but results in little to no activation when the S39 variant is used (**S16 Fig**). In contrast, 3C^pros^ from non-enteroviral picornaviruses exhibit different cleavage and activation patterns when comparing the S39 and P39 variants. For instance, 3C^pro^ from Aichi virus (Aichi) resembles SARS-CoV-2 3CL^pro^ by resulting in activation of inflammasomes reconstituted with CARD8 S39 but not the P39 variant whereas rosavirus (Rosa2) 3C^pro^ resulted in activation regardless of the variant (**S16 Fig**). Thus, a single amino acid change in CARD8 functions as a viral specificity switch between coronaviruses and enteroviruses, and even among diverse picornaviruses, underscoring the importance of pathogen-driven evolution in shaping inflammasome responses.

## Discussion

As is clear from the ongoing Coronavirus Disease 2019 (COVID-19) pandemic, understanding the molecular mechanisms that drive viral sensing and inflammatory pathogenesis during infection remains key to developing rationalized, host-directed treatments to support antiviral defense or quell severe disease. CARD8 is expressed in airway epithelia [45] and other cell types, including monocytes and T cells [32,34,46] that are physiologically relevant for respiratory coronaviruses and picornaviruses [45,47,48], including SARS-CoV-2 [35–37]. Our data showing that SARS-CoV-2 infection activates the CARD8 inflammasome in THP-1 cells is consistent with findings that inflammasome activation contributes to severe COVID-19 and suggests that the CARD8 inflammasome in monocytes and macrophages contribute to inflammation in COVID-19 patients.

Based on our results and the finding that FcγR-mediated uptake of SARS-CoV-2 virions into monocytes leads to abortive infection [37], we speculate that CARD8-dependent pyroptosis contributes to the poor permissiveness of myeloid cells for SARS-CoV-2 [49], wherein the myeloid compartment may not substantially contribute to viral load but likely impacts immunopathology via inflammasome-driven inflammation [50,51]. Prior reports have proposed a similar role for the NLRP3 inflammasome in myeloid cells [35–37]. One potential model is that CARD8-dependent GSDMD pore formation contributes to NLRP3 inflammasome activation, which also offers an explanation for CARD8-dependent release of IL-1β [30]. Interestingly, the SARS-CoV-2 3CL^pro^ also cleaves and activates NLRP1 in airway epithelia [12]. This results in cell death via a noncanonical NLRP1>CASP8>CASP3>GSDME inflammasome pathway, suggesting that the cellular context of inflammasome responses may uniquely shape antiviral defense and/or inflammation.

Beyond SARS-CoV-2, we demonstrate that 3CL^pros^ from human-relevant coronaviruses such as 229E, NL63, HKU1, SARS, and MERS also cleave CARD8 at the evolutionarily dynamic Q37 site, leading to activation of the CARD8 inflammasome and release of pro-inflammatory cytokines such as IL-1β. Furthermore, numerous 3C^pros^ from human-relevant picornaviruses including the enteroviruses CVB3, PV1, EV68, HRV, as well as non-enteroviruses such as encephalomyocarditis virus, human parechovirus, Aichi virus, hepatitis A virus, salivirus, and rosavirus also cleave and activate the CARD8 inflammasome. Although each of these viruses infect humans, their disease outcomes range from mild inflammation to acute respiratory distress syndrome, and we speculate that a multitude of variables such as cell tropism and viral replication kinetics dictate the outcome of virus-induced inflammasome activation in antiviral immunity and pathogenesis.

The potential role of human CARD8 in inflammatory pathogenesis raised the possibility that bats, the presumed reservoir for many human-relevant Beta-CoVs [41], may limit disease by avoiding 3CL^pro^-induced inflammasome activation. Consistent with this possibility, we found that CARD8 was either lacking in bats, or coronavirus 3CL^pros^ antagonize rather than activate megabat CARD8, reminiscent of prior work demonstrating that bats have a dampened NLRP3 inflammasome response [52]. We infer that viral evasion or antagonism of inflammasomes may be a general mechanism underlying the diminished immunopathologies of reservoir hosts of pathogenic viruses.

We also identified a high-frequency nonsynonymous SNP in human CARD8 that changes its specificity for viral proteases. The CARD8 P39 variant does not detect coronavirus 3CL^pros^ and, instead, enables sensing of several enteroviral 3C^pros^, including PV1, EV68, and HRV, suggestive of an evolutionary trade-off for CARD8-mediated detection of different groups of human viruses. Given the impact of SNPs on human CARD8 sensing of pathogenic viruses, it is tempting to speculate that diminished CARD8 inflammasome activation may be a contributing factor to variation in COVID-19 disease outcomes, and more generally for other human pathogenic coronavirus and picornavirus infections. Further studies are required to establish this connection.

Taken together, our findings establish CARD8 as a rapidly evolving, polymorphic, innate immune sensor of positive-sense RNA viruses. We demonstrate that CARD8 has the capacity to detect viral proteases from at least 3 viral families that include important human pathogens: *Coronaviridae*, *Picornaviridae*, and *Retroviridae*. This diverse pathogen sensing is the result of viral protease site mimics in the disordered N-terminus of CARD8, which allows activation by one or more viruses using the same tripwire region. Our results also indicate that CARD8 evolution may dynamically reshape the repertoire of host-specific sensing of viral infection. Thus, our work not only identifies an important new mechanism of sensing coronavirus infection in humans, but also builds on the emerging concept that ETI is an important mechanism of pathogen recognition, including the use of host mimicry of viral polyprotein cleavage motifs as an evolutionary strategy in the ongoing arms race between host and viruses.

## Materials and methods

### Motif generation and search

To build the Beta-CoV 3CL^pro^ cleavage motif, 995 nonredundant Beta-CoV polyprotein sequences were collected from the Viral Pathogen Resource (ViPR) [53] and aligned with 5 well-annotated reference enteroviral polyprotein sequences from RefSeq (**S1A and S1B Fig**). P1 and P1’ of the annotated cleavage sites across the RefSeq sequences served as reference points for putative cleavage sites across the 995 ViPR sequences (**S1 Table**). Four amino acid residues upstream (P4-P1) and downstream (P1’-P4’) of each cleavage site were extracted from every MAFFT-aligned [54] polyprotein sequence, resulting in 1000 sets of cleavage sites (RefSeq sites included) (**S1 Table**). Each set of cleavage sites representative of each polyprotein was then concatenated (**S2 Table**). Next, duplicates were removed from the concatenated cleavage sites (**S2 Table**). The remaining 60 nonredundant, concatenated cleavage sites were then split into individual 8-mer cleavage sites and were aligned using MAFFT [54] to generate Geneious-defined [55] sequence logo information at each aligned position (**S2 Table**). Pseudo-counts to the position-specific scoring matrix were adjusted as described previously [14] and a motif *p*-value cutoff of 0.00231 corresponding to detection of 99% of the initial polyprotein cleavage sites was selected (**S1 Fig**).

#### Sequence alignments and phylogenetic trees

Complete polyprotein sequences from 60 Beta-CoVs with nonredundant cleavage sites (see “Motif generation and search” section above) were downloaded from ViPR. Sequences were aligned using MAFFT [54] and a neighbor-joining phylogenetic tree was generated using Geneious software [55].

#### Evolutionary analyses

For phylogenomic analyses of CARD8 and NLRP1 (**Fig 3A)**, human CARD8 (accession NP_001338711) and human NLRP1 (accession NP_127497.1) were used as BLASTP [56] search queries against the indicated mammalian proteomes (**Fig 3A)**. A species was determined to have an ortholog if it had a protein with >50% sequence identity, >70% sequence coverage, and was the bidirectional best hit to the indicated human protein. The species tree shown in **Fig 3A** is based on NCBI Common Tree (https://www.ncbi.nlm.nih.gov/Taxonomy/CommonTree/wwwcmt.cgi). To identify regions of mammalian CARD8s that are orthologous to the 3CL^pro^ cleavage site (Q349) in *R*. *aegyptiacus* CARD8, a 50 amino acid region of *R*. *aegyptiacus* CARD8 that was centered on the Q349 cleavage site was used as a BLASTP query against the entire RefSeq protein database with a 60% sequence identity cutoff. A single CARD8 sequence from each species (**S12 Fig)** was aligned using MAFFT [54] and trimmed to only include the 8 amino acids spanning the cleavage site.

For positive selection analyses, primate nucleotide sequences that aligned to human full-length CARD8 (Protein: NP_001338711, mRNA: NM_001351782.2) were downloaded from NCBI and aligned using MAFFT [54]. Only 8 other primate sequences, only from hominoids and Old World monkeys, were fully alignable to full-length human CARD8 (sequence accessions in S3 Table). Maximum likelihood (ML) tests were performed with codeml in the PAML software suite [57] or using BUSTED [58] on the DataMonkey [59] server. For PAML, aligned sequences were subjected to ML tests using NS sites models disallowing (M7) or allowing (M8) positive selection. The *p*-value reported is the result of a chi-squared test on twice the difference of the log likelihood (lnL) values between the 2 models using 2 degrees of freedom. We confirmed convergence of lnL values by performing each analysis using 2 starting omega (dN/dS) values (0.4 and 1.5). Results are reported from analyses using the F61 codon frequency model. Analyses with the F3x4 model gave similar results. For evolutionary analyses of regions of CARD8, the full-length alignment was truncated to only include codons 1 to 161 (“tripwire” region) or 162 to 537 (FIIND-CARD region) and PAML or BUSTED analyses were performed as described above.

We used 3 independent methods to estimate individual codons within CARD8 that have been subject to positive selection (**S4 Table**). PAML was used to identify positively selected codons with a posterior probability greater than 0.90 using a Bayes Empirical Bayes (BEB) analysis and the F61 or F3x4 codon frequency models. The same CARD8 alignment was also used as input for FEL [60] and FUBAR [61] using the DataMonkey [59] server. In both cases, default parameters were used and codons with a signature of positive selection with a *p*-value of <0.1 are reported. In all cases, codon numbers correspond to the amino acid position and residue in human CARD8 (NCBI accession NP_001338711).

#### Plasmids and constructs

Megabat CARD8, CASP1, IL-1β, and all 3CL^pro^ sequences were ordered as either gBlocks (Integrated DNA Technologies, San Diego, CA) or Twist Gene Fragments (Twist Biosciences, South San Francisco, CA). All sequences are found in **S7 Table**. Vectors containing the coding sequences of human CARD8, ASC, human CASP1, human IL-1β-V5, and TEV^pro^ were previously described [27]. Vector psPAX2 containing the untagged coding sequence for HIV-1 gag-pol was a gift from Didier Trono (Addgene plasmid # 12260).

For CARD8 cleavage assays, the coding sequences of human CARD8 (NCBI accession NP_001171829.1), human CARD8 mutants (Q37A, Q37A Q61A, S39P, R40W), human CARD8 TEV, *R*. *aegyptiacus* (megabat) CARD8 (NCBI accession XP_016010896), and megabat CARD8 TEV were cloned into the pcDNA5/FRT/TO backbone (Invitrogen, Carlsbad, CA) with an N-terminal 3xFlag-mCherry tag. For CARD8 activation and complementation assays, the same sequences were cloned into the pQCXIP vector backbone (Takara Bio, Mountain View, CA) or the doxycycline-inducible plasmid pLKO-puro (gift from Melissa Kane) with a C-terminal Myc tag, respectively. Megabat CASP1 (NCBI accession KAF6464288) and megabat IL-1β (NCBI accession KAF6447073), also from *R*. *aegyptiacus*, were cloned in the same vector as their respective human orthologues. 3CL^pro^ sequences were cloned with an N-terminal HA tag into the QCXIP vector backbone, flanked by polyprotein cleavage sites fused to N-terminal eGFP and C-terminal mCherry (**S2 Fig)**. 3C^pro^ constructs were described previously [14].

Single point mutations were made using overlapping stitch PCR. All plasmid stocks were sequenced across the entire inserted region to verify that no mutations were introduced during the cloning process. The primers used for cloning are described in **S7 Table**.

#### Cell culture and transient transfection

All cell lines (HEK293T, HEK-Blue-IL-1β) are routinely tested for mycoplasma by PCR kit (ATCC, Manassas, VA) and kept at a low passage number to maintain less than 1 year since purchase, acquisition, or generation. HEK293T cells were obtained from ATCC (catalog # CRL-3216) and HEK-Blue-IL-1β cells were obtained from Invivogen (catalog # hkb-il1b) and all lines were verified by those sources and were grown in complete media containing DMEM (Gibco, Carlsbad, CA), 10% FBS, and appropriate antibiotics (Gibco, Carlsbad, CA). THP-1 cells were purchased from ATCC and grown in complete media containing RPMI (Gibco, Carlsbad, CA), 10% FBS, and 1% L-glutamine. For transient transfections, HEK293T cells were seeded the day prior to transfection in a 24-well plate (Genesee, El Cajon, CA) with 500 μl complete media. Cells were transiently transfected with 500 ng of total DNA and 1.5 μl of Transit X2 (Mirus Bio, Madison, WI) following the manufacturer’s protocol. HEK-Blue IL-1β reporter cells (Invivogen, San Diego, CA) were grown and assayed in 96-well plates (Genesee, El Cajon, CA).

#### Generation of knockout and transgenic cell lines

*CARD8* KOs in HEK293T cells were generated similarly to *NLRP1* KOs described in [14]. Briefly, lentivirus-like particles were made by transfecting HEK293T cells with the plasmids psPAX2 (gift from Didier Trono, Addgene plasmid # 12260), pMD2.G (gift from Didier Trono, Addgene plasmid # 12259), and either pLB-Cas9 (gift from Feng Zhang, Addgene plasmid # 52962) [62] or plentiGuide-Puro, which was adapted for ligation-independent cloning (gift from Moritz Gaidt) [63]. Conditioned supernatant was harvested 48 and 72 h post-transfection and used for spinfection of HEK293T cells at 1,200 × *g* for 90 min at 32°C. Cells with stable expression of Cas9 were selected in media containing 100 μg/ml blasticidin, 48 h post-spinfection. Blasticidin-resistant cells were then transduced with sgRNA-encoding lentivirus-like particles and selected in media containing 0.5 μg/ml puromycin. Cells resistant to blasticidin and puromycin were single cell cloned by limiting dilution in 96-well plates and confirmed as KOs by Sanger sequencing. *CARD8* and *CASP1* KO THP-1 cells were generated as described previously [64]. Briefly, *CARD8*-specific and *CASP1*-specific sgRNAs were designed using CHOPCHOP [65] and cloned into a plasmid containing U6-sgRNA-CMV-mCherry-T2A-Cas9 using ligation-independent cloning. THP-1 cells were electroporated using the BioRad GenePulser Xcell. After 24 h, mCherry-positive cells were sorted and plated for cloning by limiting dilution. Monoclonal lines were validated as KOs by deep sequencing and OutKnocker analysis, as described previously [66,67]. KO lines were further validated by immunoblot and functional assays. sgRNA used to generate KOs are described in **S7 Table**. To make THP-1 cells susceptible to SARS-CoV-2 infection [68], ACE2 and TMPRSS2 expressing THP-1 cells were made using the same lentiviral transduction protocol as described above, but using the transfer plasmid PpWIP-IRES-Bla-AK-ACE2-IRES-TMPRSS2 (gift from Sonja Best). THP-1 cells were selected with 10 μg/ml blasticidin.

#### THP-1 treatments and viral infections of WT and CARD8 KO THP-1 cells

Around 50,000 to 100,000 THP-1 cells were seeded per well in 96-well round bottom plates in 50 μl OptiMEM containing 500 ng/mL Pam3CSK4 for 6 h, followed by treatment with VbP (10 μM) or infection with the coronaviruses hCoV-229E (BEI NR-52726) or SARS-CoV-2 (USA/WA-1/2020, BEI NR-52281) at indicated multiplicities of infection (MOIs). Supernatants were harvested for the detection of IL-1β (see below), 48 h post-treatment or infection. Cells were transferred to a white-walled 96-well assay plate and mixed with an equal volume of Cell Titer Glo reagent (Promega). Measurements for fluorescence at 544–15 nm (excitation), 620–20 nm (emission), or luminescence at 555–70 (emission) were taken following incubation at room temperature, rocking for 10 min.

#### CARD8 complementation and infection

Lentiviruses used for complementation were generated by transfecting HEK293T in 6-well format (2 mL total volume) using TransIT-LT1 reagent (Mirus Bio LLC). Cells were cotransfected with doxycycline-inducible pLKO-CARD8 complementing constructs, psPAX2, and pMD2.G, and media was replaced the next day. Virus-containing supernatants were harvested 2 d post-transfection and used to transduce *CARD8* KO THP-1 cells. *CARD8* KO THP-1 cells were seeded at 2 × 10^5^ cells/well in 6-well plates and transduced with 800 μL virus in the presence of 1 μg/mL polybrene via spinoculation at 1,200 × g for 90 min at 32°C, then puro-selected 24 h post-transduction. Following selection, cells were kept at low passage for all functional assays.

For infection experiments with complemented cell lines, 15,000 THP-1 cells (parental, *CARD8* KO, and complemented *CARD8* KO cells) were seeded in white 96-well plates. After 48 h, cells were treated with doxycycline (10 ng/μL) and infected with 200 PFU hCoV-229E unless otherwise indicated. Cells were incubated at 33°C for 48 h. Cell viability was measured using the CellTiter-Glo Luminescent Cell Viability kit (Promega). Briefly, 48 h post-infection, half of the uninfected cells were treated with Triton-X (1% final solution). The CellTiter-Glo Buffer was mixed with the CellTiter-Glo Substrate and added to each well. The cells were gently rocked for 2 min and then incubated at room temperature for an additional 10 min. Luminescence was read on a BioTek Cytation5 Imaging Reader (Agilent). To calculate percent viability, the average luminescence units for Triton-X treated cells was subtracted from the virus infected cells. This value was divided by the luminescence units for Triton-X subtracted from uninfected, untreated cells. Experiments were performed 3 to 10 independent times with 3 to 6 replicates per condition.

#### CARD8 cleavage assays

100 ng of epitope-tagged human CARD8 (WT, Q37A, Q37A Q61A, S39P, R40W), human CARD8 TEV, megabat CARD8 WT, or megabat CARD8 TEV was cotransfected with either HA-tagged QCXIP empty vector (“−”), 250 ng of TEV^pro^, 250 ng of untagged HIV-1^pro^ (HIV-1 gag-pol carrying HIV-1 protease activity), 5 ng of HA-tagged 3CL^pro^, or 250 ng of HA-tagged 3C^pro^-encoding constructs. The cells were harvested, lysed in 1× NuPAGE LDS sample buffer (Invitrogen, Carlsbad, CA) containing 5% β-mercaptoethanol (Fisher Scientific, Pittsburg, PA), and immunoblotted with antibodies described in **S8 Table**, 24 h post-transfection.

#### CARD8 activity assays

To reconstitute the human CARD8 inflammasome, 100 ng of human CASP1 and 50 ng of human IL-1β-V5 were cotransfected with 50 ng of either HA-tagged QCXIP empty vector, WT, or mutant pQCXIP-CARD8-Myc constructs in *CARD8* KO HEK293T cells. To reconstitute the megabat CARD8 inflammasome, 10 ng of megabat CASP1 and 50 ng of megabat IL-1β-V5 were cotransfected with 2 ng megabat CARD8 constructs in *CARD8* KO HEK293T cells. These cells were further cotransfected with either empty vector (“−”), 250 ng of TEV^pro^, 100 ng of untagged HIV-1 gag-pol (with HIV-1 protease activity), 5 ng of HA-tagged 3CL^pro^, 100 ng of HA-tagged enteroviral 3C^pro^, or 20 ng of HA-tagged non-enteroviral 3C^pro^-encoding constructs. After 24 h, cells were harvested and lysed in 1× NuPAGE LDS sample buffer containing 5% β-mercaptoethanol and immunoblotted with antibodies described in **S8 Table**, or culture media was harvested for quantification of IL-1β levels by HEK-Blue assays (see below). Appearance of the mature p17 band of IL-1β indicates successful assembly and activation of the inflammasome.

#### HEK-Blue IL-1β assay

To quantify the levels of bioactive IL-1β released from cells, we employed HEK-Blue IL-1β reporter cells (Invivogen, San Diego, CA). In these cells, binding to IL-1β to the surface receptor IL-1R1 results in the downstream activation of NF-kB and subsequent production of secreted embryonic alkaline phosphatase (SEAP) in a dose-dependent manner [14]. SEAP levels were detected using a colorimetric substrate assay, QUANTI-Blue (Invivogen, San Diego, CA), by measuring an increase in absorbance at OD655.

Culture supernatant from inflammasome-reconstituted HEK293T cells or HEK293T *CARD8* KO cells that had been transfected with 3CL^pro^ was added to HEK-Blue IL-1β reporter cells plated in 96-well format in a total volume of 200 μl per well. On the same plate, serial dilutions of recombinant human IL-1β (Invivogen, San Diego, CA) were added to generate a standard curve for each assay. SEAP levels were assayed by taking 20 μl of the supernatant from HEK-Blue IL-1β reporter cells and adding to 180 μl of QUANTI-Blue colorimetric substrate following the manufacturer’s protocol, 24 h later. After incubation at 37°C for 30 to 60 min, absorbance at OD655 was measured on a BioTek Cytation5 plate reader (BioTek Instruments, Winooski, VT), and absolute levels of IL-1β were calculated relative to the standard curve. All assays, beginning with independent transfections or infections, were performed in triplicate.

#### Immunoblotting and antibodies

Harvested cell pellets were washed with 1× PBS and lysed with 1× NuPAGE LDS sample buffer containing 5% β-mercaptoethanol at 98°C for 10 min. The lysed samples were spun down at 15,000 RPM for 2 min, followed by loading into a 4% to 12% Bis-Tris SDS-PAGE gel (Life Technologies, San Diego, CA) with 1× MOPS buffer (Life Technologies, San Diego, CA) and wet transfer onto a nitrocellulose membrane (Life Technologies, San Diego, CA). Membranes were blocked with PBS-T containing 5% bovine serum albumin (BSA) (Spectrum, New Brunswick, NJ), followed by incubation with primary antibodies for V5 (IL-1β), FLAG (mCherry-fused CARD8 for protease assays), Myc (CARD8-Myc for activation assays), HA (viral protease), or GAPDH. Membranes were rinsed 3 times in PBS-T and then incubated with the appropriate HRP-conjugated secondary antibodies. Membranes were rinsed again 3 times in PBS-T and developed with SuperSignal West Pico PLUS Chemiluminescent Substrate (Thermo Fisher Scientific, Carlsbad, CA). The specifications, source, and clone info for antibodies are described in **S8 Table**.

## Supporting information

S1 Fig Motif generation of coronaviral 3CL^pro^ polyprotein cleavage. (A) Schematic of 3CL^pro^ cleavage sites (11 red triangles) within the polyprotein of SARS-CoV-2 (SARS-2), the causative agent of COVID-19. Shown are 4 amino acids flanking each side of the cleavage site within the polyprotein. (B) Phylogenetic tree of 60 coronavirus polyprotein coding sequences depicting the Beta-CoVs sampled in this study with human-relevant coronaviruses labeled (S2 Table). (C) Training set data used to determine the motif search threshold for FIMO (S1 Table). The X-axis represents a log10 of the *p*-value reported by FIMO as an indicator for the strength of the cleavage motif hit (cleavage score). (Left) The Y-axis depicts the number of uncalled true positives, or motif hits that overlap with the initial set of 8mer polyprotein cleavage sites used to generate the motif, in the training set of coronavirus polyprotein sequences (black line). (Right) The Y-axis depicts the number of called false positive sites, or any motif hits found in the polyprotein that are not known to be cleaved by 3CL^pro^, in the training set of coronaviral polyprotein sequences (gray). A red vertical dashed line indicates the threshold that captures 99% of true positive polyprotein hits (*p*-value = 0.00231). Data for S1B Fig can be found in S1 Data. Data for S1C Fig can be found in S2 Data. Beta-CoV, betacoronavirus; COVID-19, Coronavirus Disease 2019; SARS-CoV-2, Severe Acute Respiratory Syndrome Coronavirus 2; 3CL^pro^, 3CL protease.
(EPS)

S2 Fig Schematic of CARD8 and 3CL^pro^ expression constructs for cleavage assays and immunoblot depicting HA-tagged SARS-2 3CL^pro^ and SARS-2 3CL^pro^ mutant. (A) Schematic of the expression constructs used for CARD8 cleavage assays. Full-length CARD8 was fused to a 3xFlag-mCherry domain to increase the stability and our ability to detect viral protease cleavage products in the N-terminus of CARD8. Below are shown the expected sizes of full-length and FIIND-processed CARD8, as well as the expected sizes that would result from viral protease cleavage at the indicated sites. (B) Schematic of the expression constructs used for 3CL^pros^ (nsp5s). A region of the viral polyprotein spanning the C-terminal 9 residues of nsp4 through the N-terminal 9 residues of nsp6 was cloned between eGFP and mCherry-HA. Inactive protease is expressed as a full-length fusion protein (92 kDa predicted molecular weight). Active 3CL^pro^ cleaves at the indicated polyprotein cleavage sites (red triangles), liberating the active protease from the construct and resulting in an HA-tagged mCherry product (30 kDa predicted molecular weight). (C) An expanded view of the anti-HA-stained immunoblot shown in **Fig 1B** highlighting uncleaved (Uncleaved 3CL^pro^ fusion protein) and cleaved (3CL^pro^ cleavage product) HA-tagged protein products. FIIND, function-to-find domain; 3CL^pro^, 3CL protease.
(EPS)

S3 Fig CARD8 cleavage by viral proteases results in inflammasome activation and IL-1β maturation. CARD8 inflammasome assay. *CARD8* KO HEK293T cells were cotransfected using the indicated Myc-tagged CARD8 plasmid constructs, V5-IL-1β, CASP1, and HA-tagged protease constructs (SARS-CoV-2 3CL^pro^ (SARS-2 3CL^pro^), SARS-CoV-2 3CL^pro^ catalytic mutant C145A (3CL^pro^ mutant), HIV-1 gag-pol (HIV-1^pro^), or empty vector (−)). Appearance of a mature bioactive IL-1β (p17) indicates inflammasome activation. HIV-1^pro^, HIV-1 protease; IL, interleukin; KO, knockout; SARS-CoV-2, Severe Acute Respiratory Syndrome Coronavirus 2; 3CL^pro^, 3CL protease.
(EPS)

S4 Fig Cleavage at Q61 is not required for CARD8 inflammasome activation by coronavirus 3CL^pro^. (A, B) *CARD8* KO HEK293T cells were transfected with the indicated CARD8 construct in the presence (“+”) or absence (“−”) of indicated proteases and plasmids expressing CASP1 and V5-tagged IL-1b. Active (SARS-2 3CL^pro^) or catalytically inactive (3CL^pro^ mutant) protease from SARS-CoV-2 was expressed as an HA-tagged fusion construct. HIV-1^pro^ was expressed from an untagged gag-pol construct. Inflammasome activation was monitored by immunoblotting for mature IL-1β (p17) (A) or measuring culture supernatant levels of bioactive IL-1β using IL1R-expressing reporter cells (B). Individual values (*n =* 3), averages, and standard deviations are shown. Data were analyzed using two-way ANOVA with Šidák’s post-test. *** = *p* < 0.001, **** = *p* < 0.0001, n.s. = not significant. Data for S4B Fig can be found in S1 Data. HIV-1^pro^, HIV-1 protease; IL, interleukin; KO, knockout; SARS-CoV-2, Severe Acute Respiratory Syndrome Coronavirus 2; 3CL^pro^, 3CL protease.
(EPS)

S5 Fig 3CL^pro^-mediated activation of the human CARD8 inflammasome depends on FIIND autoprocessing. CARD8 inflammasome activation assay depicting loss of CARD8 activation with a FIIND autoprocessing mutant (S297A). FIIND, function-to-find domain; 3CL^pro^, 3CL protease.
(EPS)

S6 Fig 3CL^pros^ used in this study demonstrate similar polyprotein cleavage. Phylogenetic tree of 3CL^pro^ protein sequences used in this study from the indicated coronaviruses. Shown next to the virus name is the sequence motif generated from the 3CL^pro^ polyprotein cleavage sites from that specific virus. 3CL^pro^, 3CL protease.
(EPS)

S7 Fig Site-specific CARD8 cleavage and inflammasome activation by diverse coronavirus 3CL^pros^. (A) Cleavage assay depicting cleavage of human CARD8 by the indicated 3CL^pros^ from diverse coronaviruses (SARS-CoV-2 (SARS-2), hCoV-229E (229E), hCoV-NL63 (NL63), hCoV-HKU1 (HKU1), SARS-CoV (SARS), MERS-CoV (MERS), and murine hepatitis virus (MHV)). (B) CARD8 inflammasome activation assay with human CARD8 WT and the indicated 3CL^pro^. (C, D) Cleavage assays mapping the cleavage specificity of diverse 3CL^pros^. Indicated proteases were cotransfected with WT and Q37A (C) or Q37A Q61A (D). MHV, murine hepatitis virus; SARS-CoV-2, Severe Acute Respiratory Syndrome Coronavirus 2; WT, wild-type; 3CL^pro^, 3CL protease.
(EPS)

S8 Fig Validation of *CARD8* KO THP-1 cells. (A) Immunoblot of WT or *CARD8* KO1 and KO2 THP-1 cells. The sgRNA used to edit *CARD8* results in a truncated CARD8 (grey arrow) that removes the CTD, including the CARD. (B, C) Indicated THP-1 cells were primed with 0.5 μg/mL Pam3CSK4 for 6 h, followed by treatment with 10 μM VbP or media only. Cell viability (B) was measured 48 h post-treatment via the Cell Titer Glo assay, and IL-1β levels (C) were measured from cell supernatants using the IL1R reporter assay as in **Fig 1D** (see **Materials and methods**). Data presented are representative of experiments performed in triplicate. Data were analyzed using two-way ANOVA with Šidák’s post-test. **** = *p* < 0.0001. Data for S8B and S8C Fig can be found in S1 Data. CARD, caspase activation and recruitment domain; CTD, C-terminal domain; IL, interleukin; KO, knockout; VbP, Val-boroPro; WT, wild-type.
(EPS)

S9 Fig CASP1 is required for CARD8-mediated sensing of coronavirus infection. WT, *CARD8*, and *CASP1* KO1 and *CASP1* KO2 THP-1 cells were primed with 0.5 μg/mL Pam3CSK4 for 6 h, followed by infection with the coronavirus hCoV-229E at an MOI of 0.4, treatment with VbP (10 μM), or treated with media alone (Mock). After 48 h, cell viability was measured using the Cell Titer Glo assay as in **Fig 2A**. Data were analyzed using one-way ANOVA with Tukey’s post-test. n.s. = not significant, **** = *p* < 0.0001. Data for S9 Fig can be found in S1 Data. CASP1, caspase-1; KO, knockout; MOI, multiplicity of infection; VbP, Val-boroPro; WT, wild-type.
(EPS)

S10 Fig Functional complementation of *CARD8* KO THP-1 cells with CARD8 variants. *CARD8* KO1 THP-1 cells complemented with the indicated CARD8 variant were primed with 0.5 μg/mL Pam3CSK4 for 6 h in the presence or absence of 100 ng/mL Dox and were treated with VbP (10 μM) or with media alone (Mock). After 48 h, cell viability was measured using the Cell Titer Glo assay as in Fig 2A. Data were analyzed using two-way ANOVA with Šidák’s post-test. n.s. = not significant, **** = *p* < 0.0001. Data for S10 Fig can be found in S1 Data. Dox, Doxycycline; KO, knockout; VbP, Val-boroPro.
(EPS)

S11 Fig WT CARD8, but not uncleavable or FIIND mutant CARD8, restores responsiveness to coronavirus infection. WT CARD8, *CARD8* KO1, or *CARD8* KO2 THP-1 cells complemented with the indicated CARD8 variant were plated into wells with 15,000 cells per well. Dox was added where indicated to a final concentration of 100 ng/mL, and cells were infected with the indicated amount (in PFUs) of hCoV-229E. After 48 h, cell viability was measured using the Cell Titer Glo assay. Data were analyzed using one-way ANOVA with Tukey’s post-test. n.s. = not significant, * = *p* < 0.05. Data for S11 Fig can be found in S1 Data. Dox, Doxycycline; FIIND, function-to-find domain; KO, knockout; PFU, plaque-forming unit; WT, wild-type.
(EPS)

S12 Fig A coronavirus 3CL^pro^ cleavage site in the CARD8 C-terminus is unique to megabats. An alignment of protein sequences homologous to the coronavirus 3CL^pro^ cleavage site in megabat (*R*. *aegyptiacus*) CARD8 is shown for indicated species (scientific name, accession number). Amino acid numbering is based on *R*. *aegyptiacus* CARD8. Changes relative to the consensus, depicted as a sequence logo at the top of the alignment, are highlighted. Four species of megabats are indicated and are unique in having a serine in the P1’ position of the cleavage site (highlighted in red). Most other species have a threonine in this position, which makes the protein uncleavable at this site (**Fig 3E**). 3CL^pro^, 3CL protease.
(EPS)

S13 Fig Rapid evolution and human polymorphism in the “tripwire” region of CARD8 that is targeted by viral proteases. An alignment of the CARD8 N-terminus (amino acids 15–65, where amino acid numbering is based on human CARD8) or “tripwire” region from humans and selected nonhuman primates. Human nonsynonymous SNP that encode CARD8 P39 and W40 are shown. Differences relative to the human reference protein sequence (accession NP_001338711) are indicated in red font. Coronavirus 3CL^pro^ (Q37 and Q61; red arrows) and HIV-1^pro^ cleavages sites in CARD8 are shown. “-” = indel. HIV-1^pro^, HIV-1 protease; SNP, single nucleotide polymorphism; 3CL^pro^, 3CL protease.
(EPS)

S14 Fig The human CARD8 S39P variant has reduced sensitivity to coronavirus 3CL^pro^ cleavage and inflammasome activation. (A) CARD8 inflammasome activation assay, where inflammasome activation is measured by CASP1-dependent processing of pro-IL-1β to p17. *CARD8* KO HEK293T cells were cotransfected with Myc-tagged constructs encoding the reference allele of human CARD8 (protein accession NP_001338711, mRNA accession NM_001351782.2) or the human CARD8 nonsynonymous SNP CARD8-P39 (rs12463023) or CARD8-W40 (rs138177358) variants and the HA-tagged SARS-CoV-2 (SARS-2) 3CL^pro^. (B) Comparison of CARD8 S39 and CARD8 P39 cleavage by diverse coronavirus 3CL^pros^. *CARD8* KO HEK293T cells were cotransfected using the indicated Flag-tagged mCherry-CARD8 fusion constructs with HA-tagged protease constructs: empty vector (“-”), SARS-2 catalytically inactive mutant (3CL^pro^ mutant) or active 3CL^pro^ from SARS-2, 229E, NL63, HKU1, SARS, MERS, or MHV. Red triangles indicate cleavage sites 3CL^pro^. IL, interleukin; KO, knockout; MHV, murine hepatitis virus; SARS-CoV-2, Severe Acute Respiratory Syndrome Coronavirus 2; SNP, single nucleotide polymorphism; 3CL^pro^, 3CL protease.
(EPS)

S15 Fig Functional complementation of *CARD8* KO THP-1 cells with human CARD8 P39 variant. WT CARD8, *CARD8* KO1, or *CARD8* KO2 THP-1 cells complemented with the CARD8 cleavage site mutant S39P were primed with 0.5 μg/mL Pam3CSK4 for 6 h in the presence or absence of 100 ng/mL Dox and were treated with VbP (10 μM) or with media alone (Mock). After 48 h, cell viability was measured using the Cell Titer Glo assay as in **Fig 2A**. Data were analyzed using one-way ANOVA with Tukey’s post-test. n.s. = not significant. **** = *p* < 0.0001. Data for S15 Fig can be found in S1 Data. Dox, Doxycycline; KO, knockout; VbP, Val-boroPro; WT, wild-type.
(EPS)

S16 Fig The CARD8 P39 variant is variably susceptible to picornavirus 3C^pros^. (A) WT HEK293T cells were cotransfected using the indicated Flag-tagged mCherry-CARD8 fusion plasmid constructs with V5-IL-1β, CASP1, and HA-tagged 3C^pro^ constructs or empty vector (“-”). 3C^pros^ from the following viruses were used: enterovirus A71 (EV71), coxsackievirus B3 (CVB3), poliovirus 1 (PV1), enterovirus D68 (EV68), human rhinovirus A (HRV), encephalomyocarditis virus (EMCV), human parechovirus A (ParA), Aichi virus (Aichi), hepatitis A virus (HepA), human salivirus A (SaliA), and human rosavirus 2 (Rosa2). Red arrows denote CARD8 fragments resulting from 3C^pro^ cleavage at indicated sites. (B) CARD8 inflammasome activation assay, where inflammasome activation is measured by CASP1-dependent processing of pro-IL-1β to p17. *CARD8* KO HEK293T cells were cotransfected with Myc-tagged constructs encoding the reference allele of human CARD8 (protein accession NP_001338711, mRNA accession NM_001351782.2) or the human CARD8 nonsynonymous SNP CARD8-P39 (rs12463023) and the indicated 3C^pro^, 3CL^pro^, or HIV-1^pro^ constructs, or empty vector (“-”). (C) IL-1β assay, which measures the release of bioactive IL-1β in the culture supernatant of cells transfected as described in (B). Individual values (*n =* 3), averages, and standard deviations shown are representative of experiments performed in triplicate. Data were analyzed using one-way ANOVA with Tukey’s post-test. n.s. = not significant. **** = *p* < 0.0001. Data for S16C Fig can be found in S1 Data. CASP1, caspase-1; HIV-1^pro^, HIV-1 protease; IL, interleukin; KO, knockout; SNP, single nucleotide polymorphism; WT, wild-type; 3C^pro^, 3C protease; 3CL^pro^, 3CL protease.
(EPS)

S1 Data Source data for graphs, phylogenetic tree, and sequence logos shown in this paper. (XLSX)

S2 Data Source data for S1C Fig. (XLSX)

S1 Raw Images Uncropped and unedited images for western blots. (PDF)

S1 Table VIPR and RefSeq betacoronavirus (Beta-CoV) polyproteins with 3CL cleavage site concatenations. (XLSX)

S2 Table Beta-CoV polyproteins with unique 8mer 3CL cleavage site concatenations. (XLSX)

S3 Table Accession numbers of CARD8 sequences used for evolutionarily analyses. (XLSX)

S4 Table Codon positions in full length CARD8 from hominoids and Old World monkeys predicted to be evolving under recurrent positive selection by PAML, FUBAR, and FEL analyses. (XLSX)

S5 Table CARD8 missense variants (>100 allele counts) mapped to GRCh38 reported in gnomAD v3.1.2. (XLSX)

S6 Table CARD8 missense variants (>100 allele counts) mapped to GRCh38 reported in gnomAD v3.1.2 represented by population with the highest allele frequency. (XLSX)

S7 Table Primers, gBlocks, Twist fragments, and sgRNA. (XLSX)

S8 Table List of antibody specifications. (XLSX)

## Abbreviations

Beta-CoV: betacoronavirus
CARD: caspase activation and recruitment domain
CASP1: caspase-1
COVID-19: Coronavirus Disease 2019
CVB3: Coxsackievirus B3
ETI: effector-triggered immunity
FIIND: function-to-find domain
GSDMD: Gasdermin D
HIV-1^pro^: HIV-1 protease
HRV: human rhinovirus
IL: interleukin
KO: knockout
MHV: murine hepatitis virus
ML: maximum likelihood
MOI: multiplicity of infection
M^pro^: main protease
SARS-CoV-2: Severe Acute Respiratory Syndrome Coronavirus 2
SEAP: secreted embryonic alkaline phosphatase
SNP: single nucleotide polymorphism
VbP: Val-boroPro
ViPR: Viral Pathogen Resource
WT: wild-type
3C^pro^: 3C protease
3CL^pro^: 3CL protease
