# Supplementary material for: Host-specific sensing of coronaviruses and picornaviruses by the CARD8 inflammasome
Source: PLoS Biol. 2023 Jun 8;21(6):e3002144. doi: 10.1371/journal.pbio.3002144 (PMC10249858; doi:10.1371/journal.pbio.3002144)

Figure 1B aFLAG

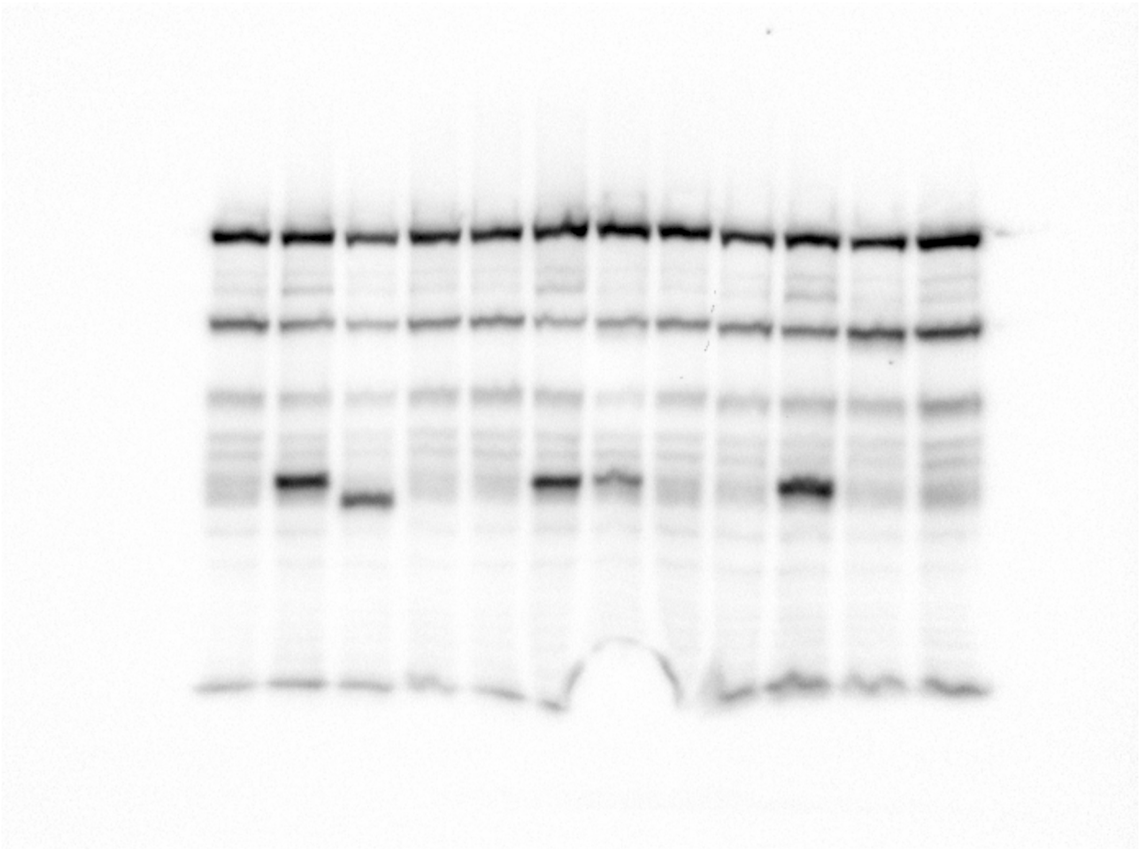

Figure 1B aHA

low

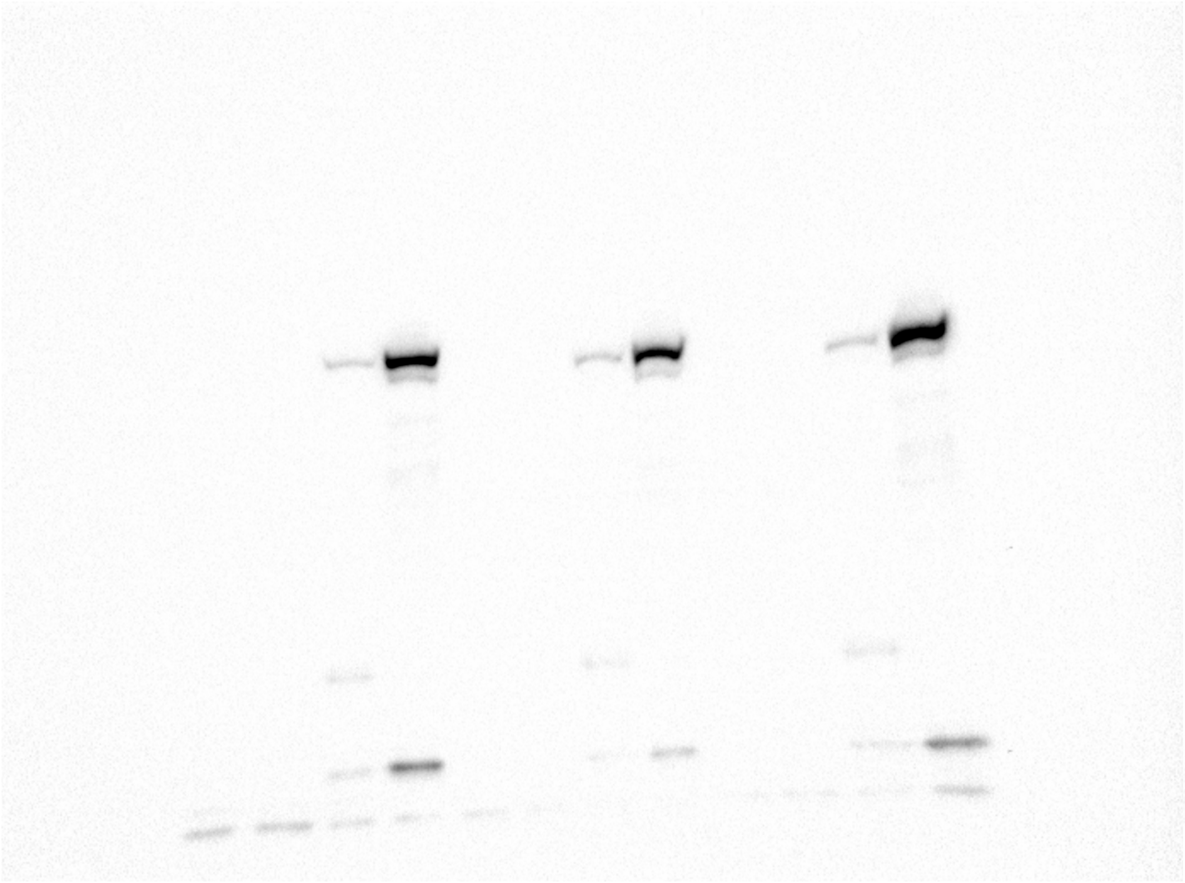

high

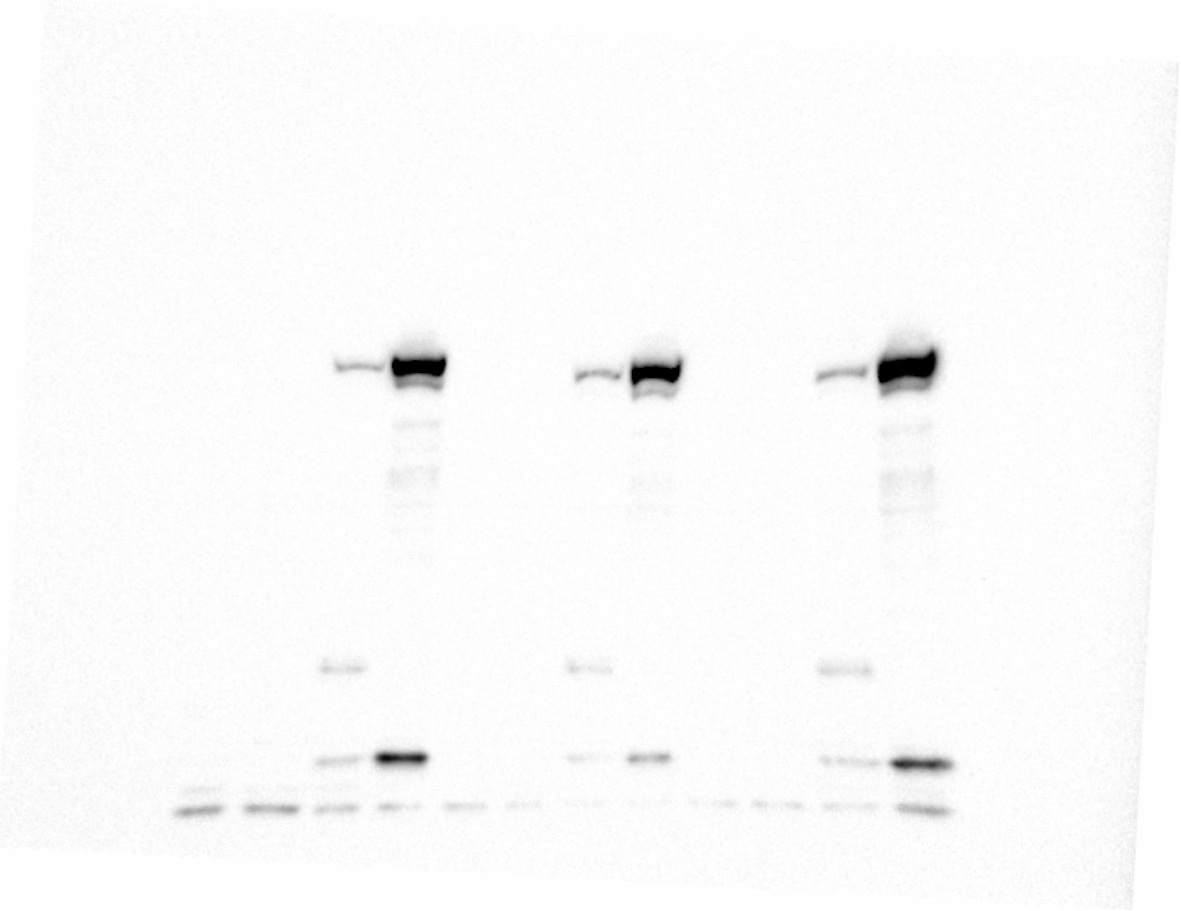

Figure 1B aGAPDH

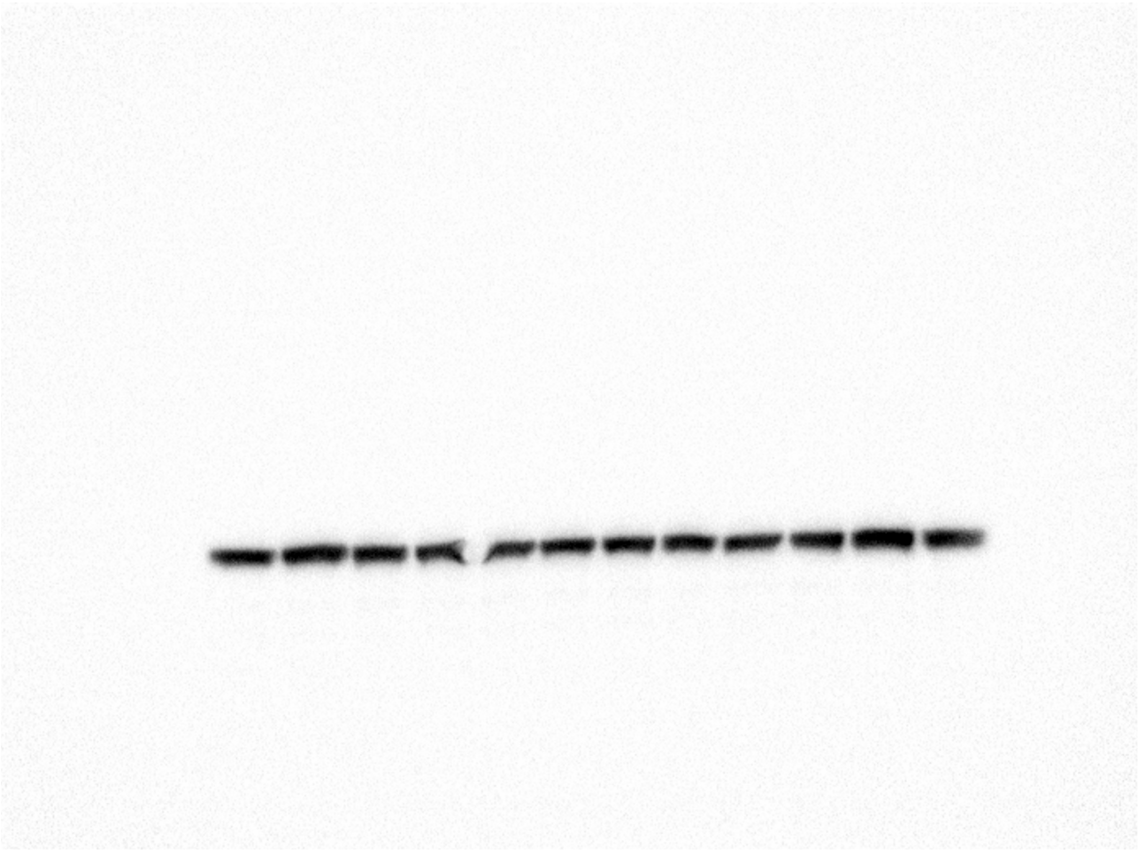

Figure 1C aV5

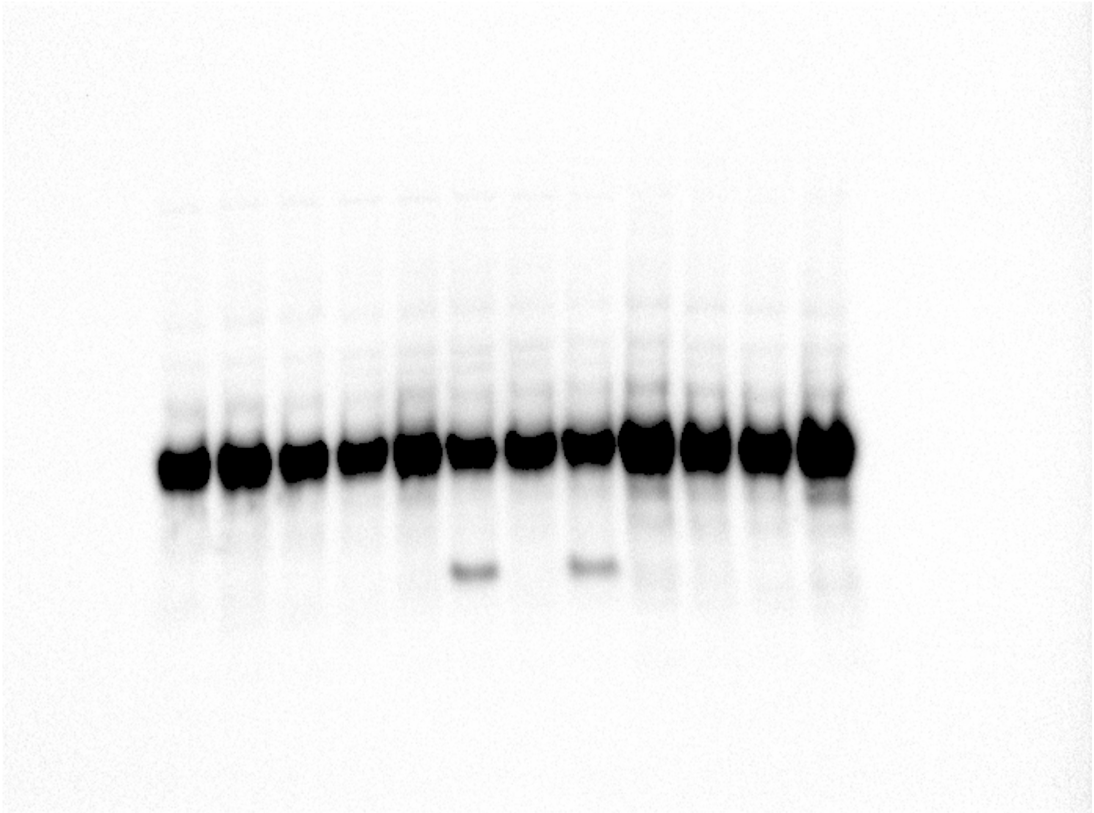

Figure 1C aHA

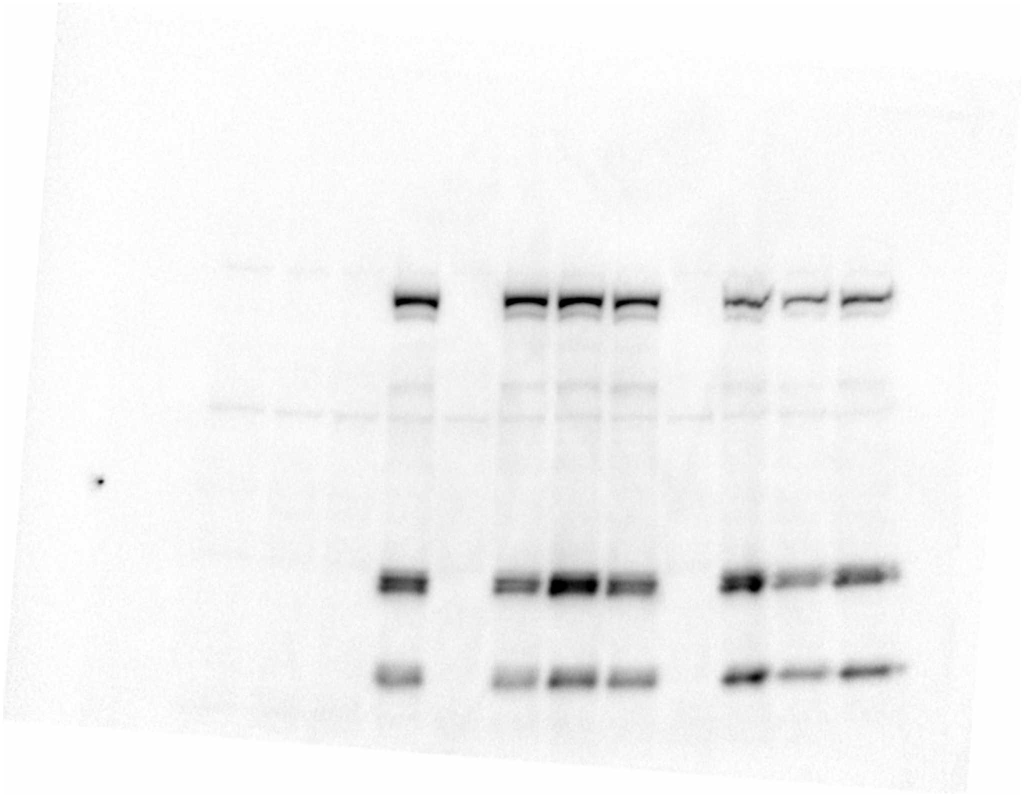

Figure 1C aGAPDH

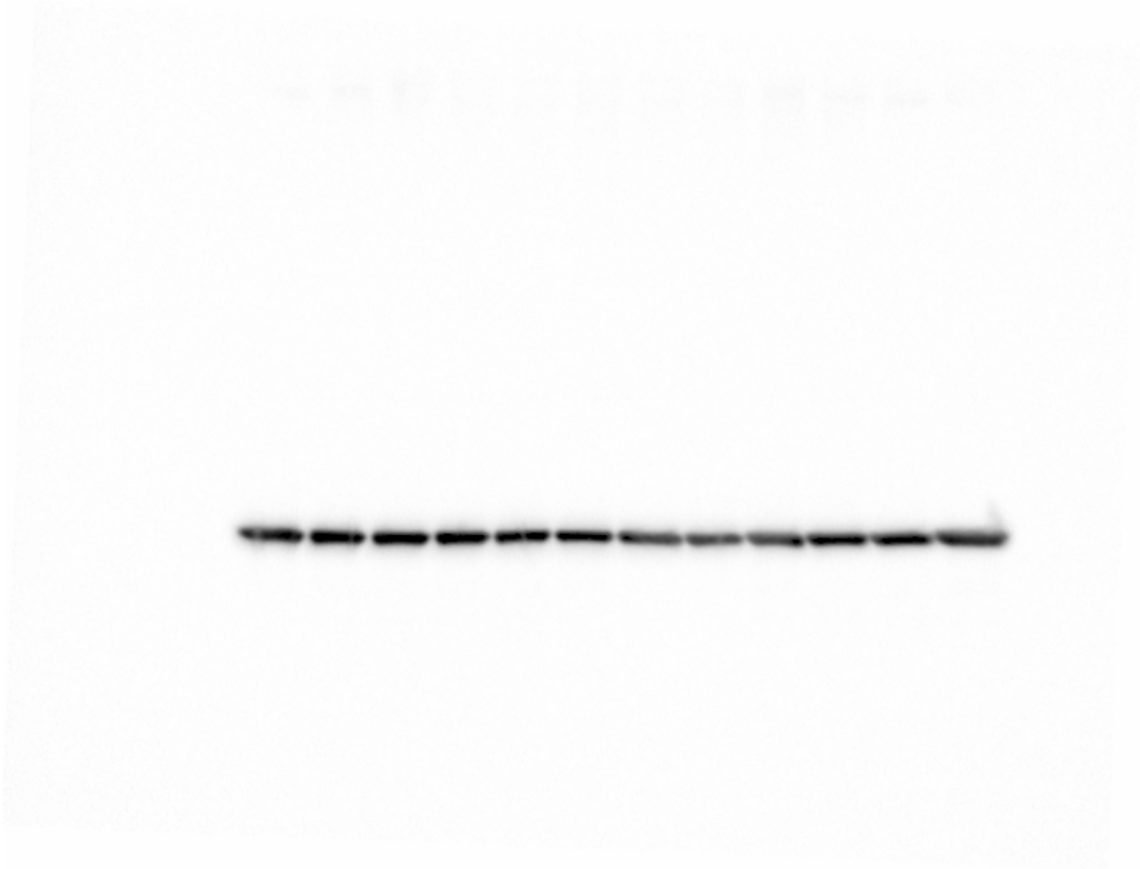

Figure 3B aFLAG

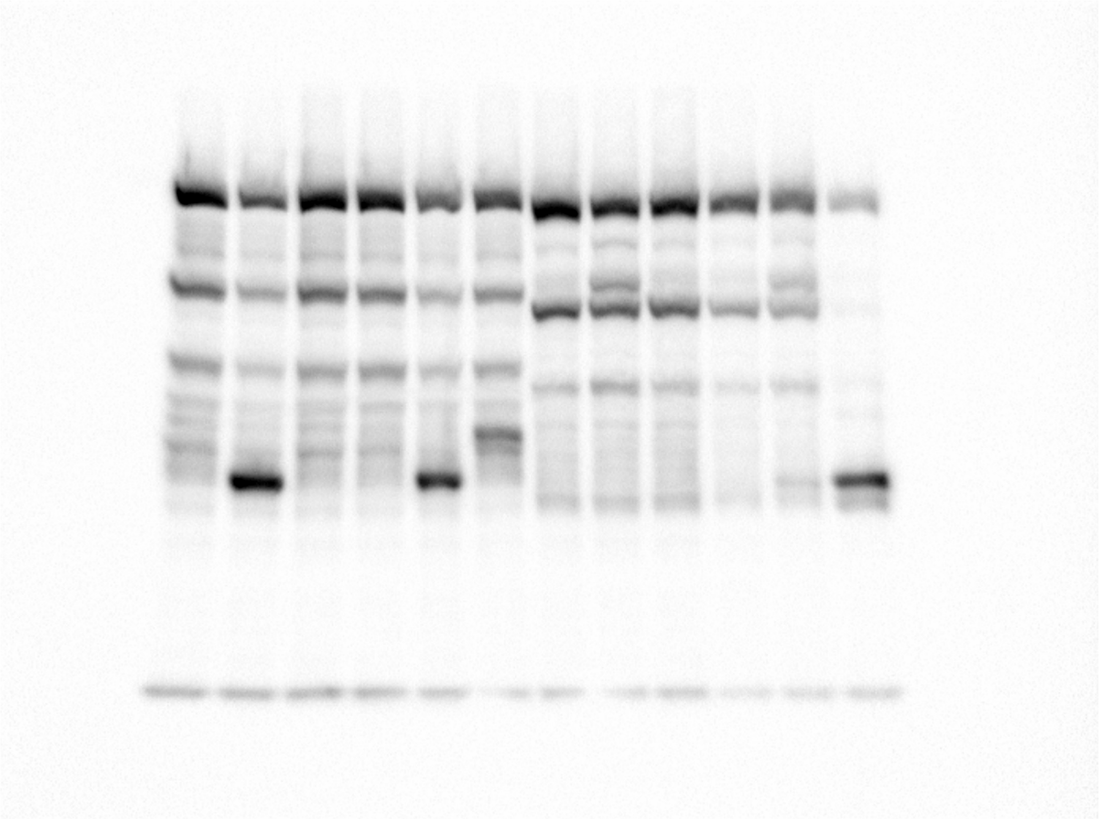

Figure 3B aHA

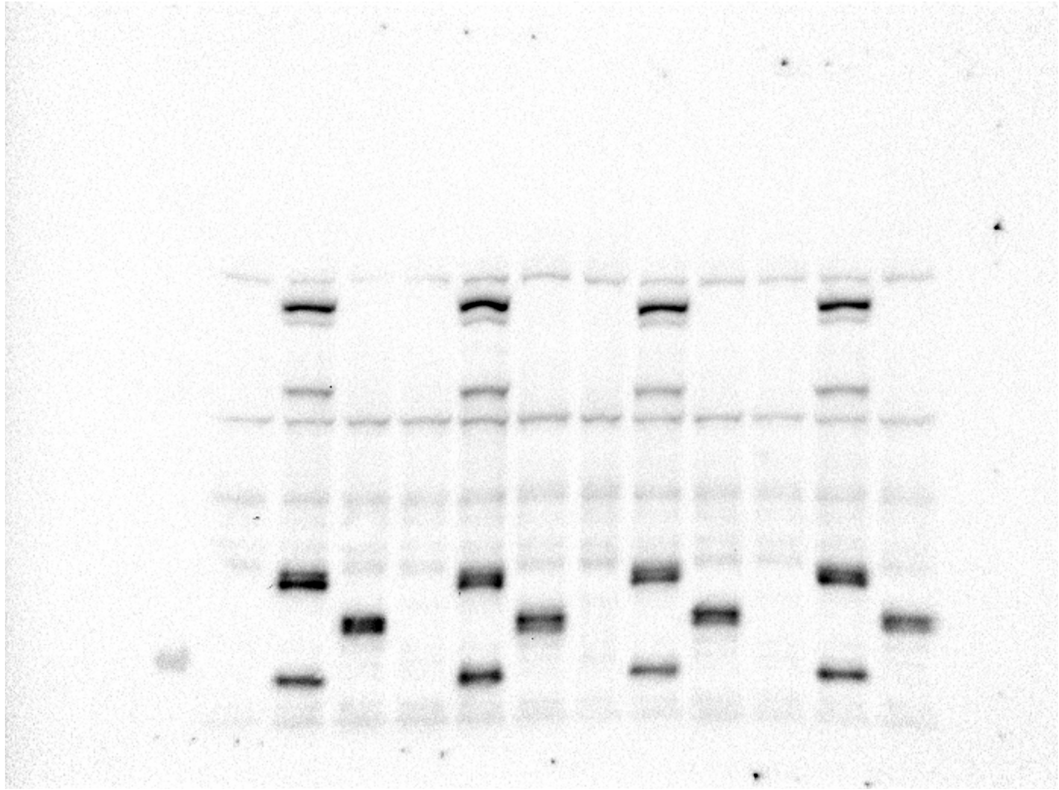

Figure 3B aGAPDH

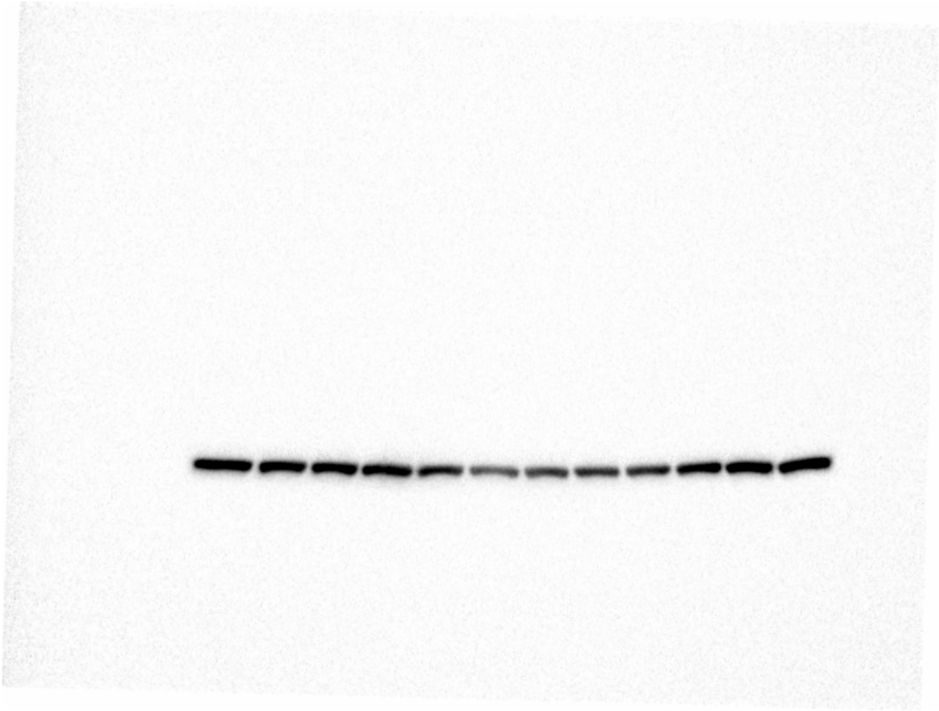

Figure 3C aFLAG

First 9

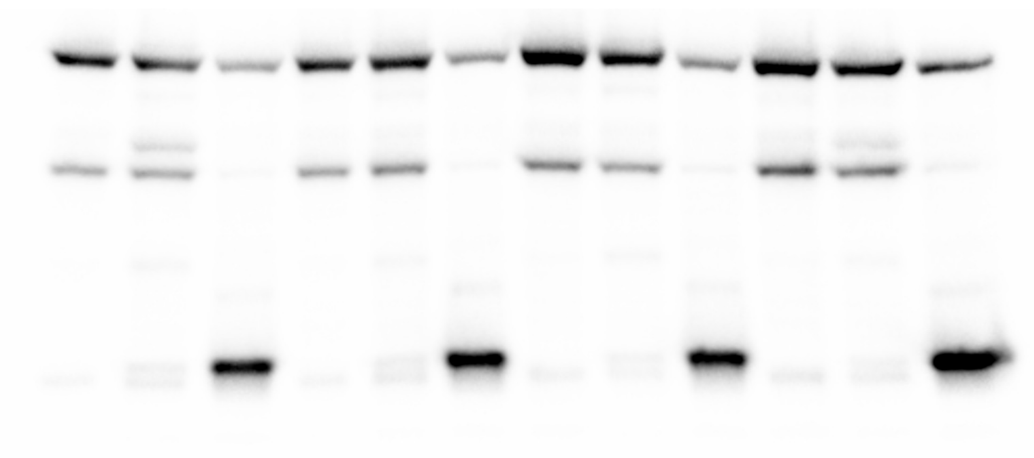

Figure 3C aHA

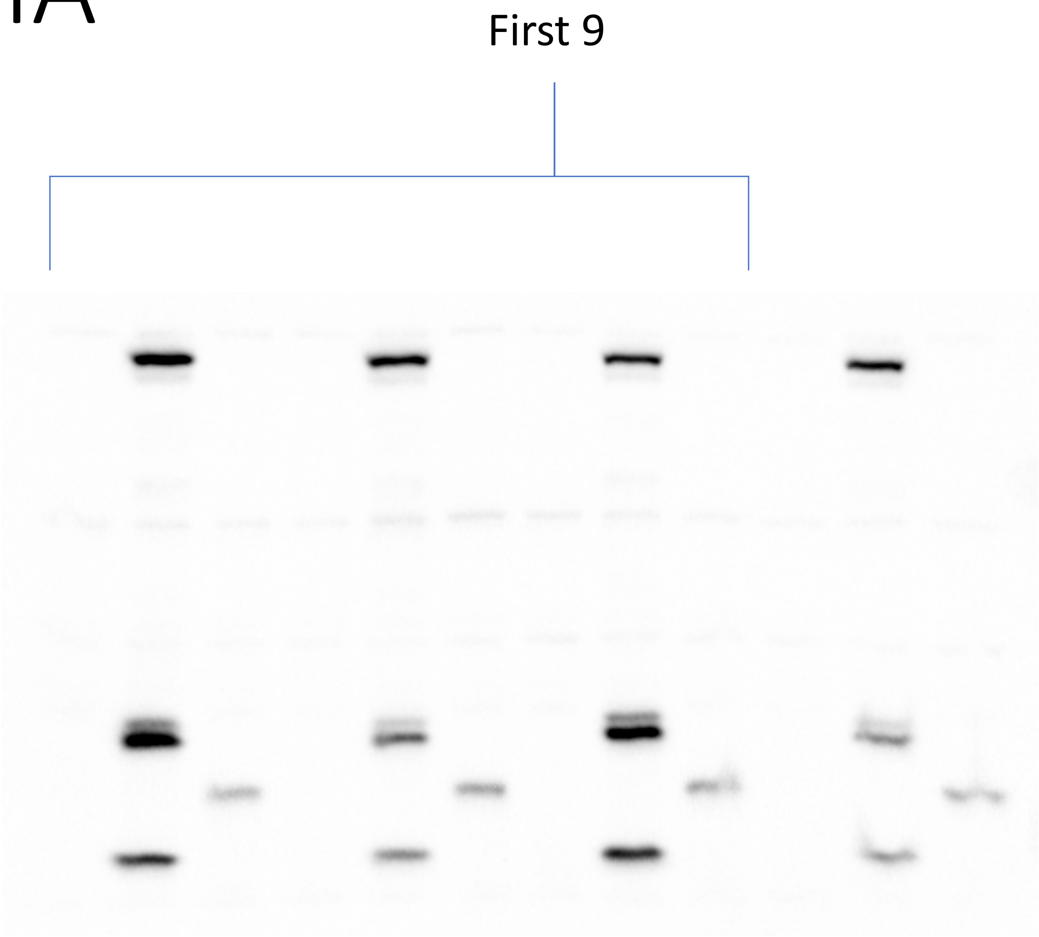

Figure 3C aGAPDH

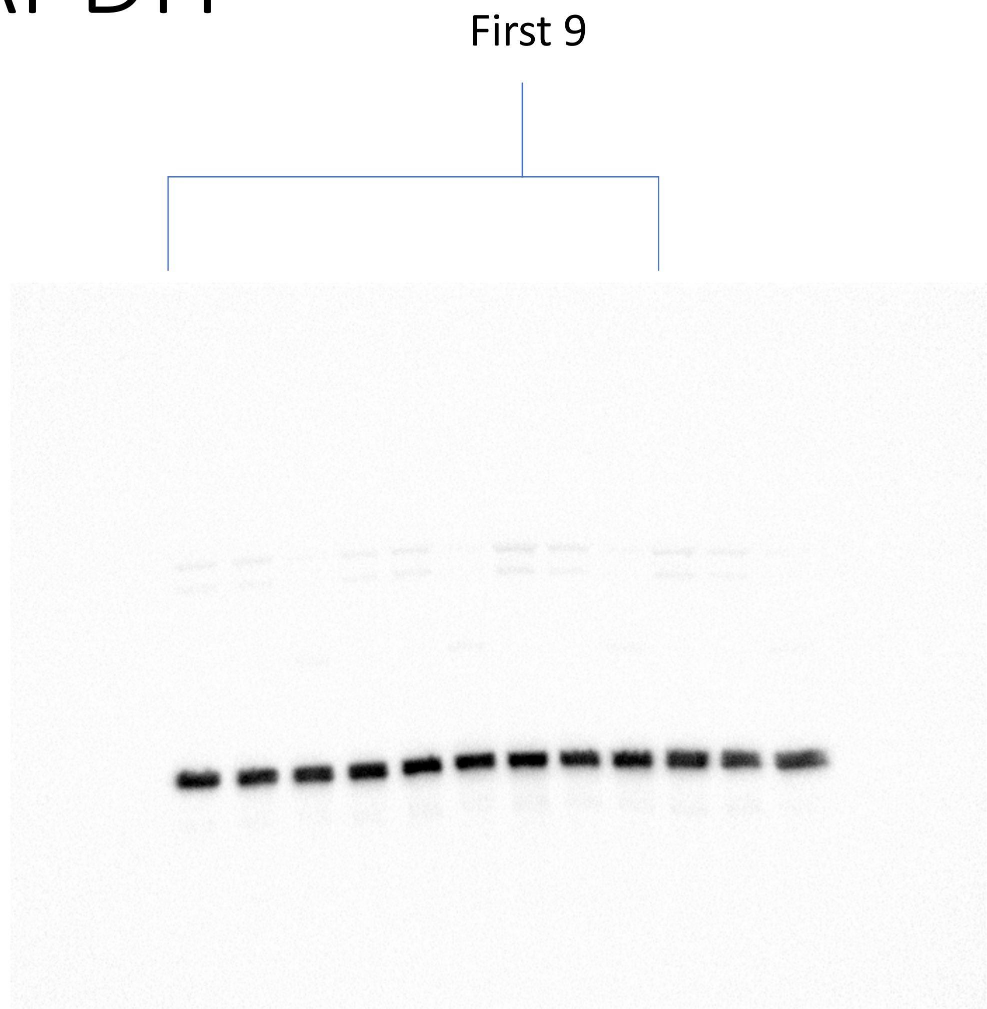

Figure 3D aV5

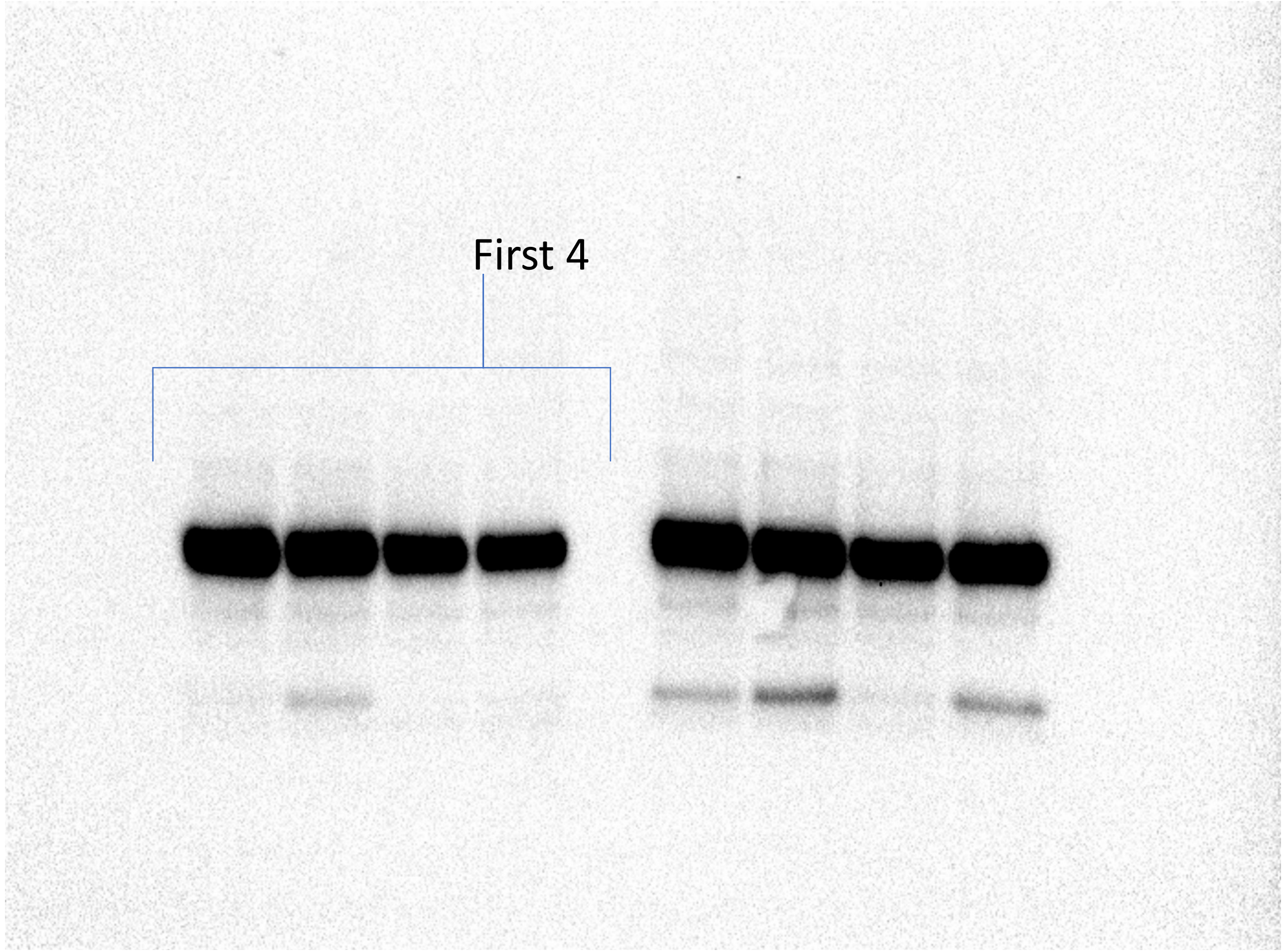

Figure 3D aMyc

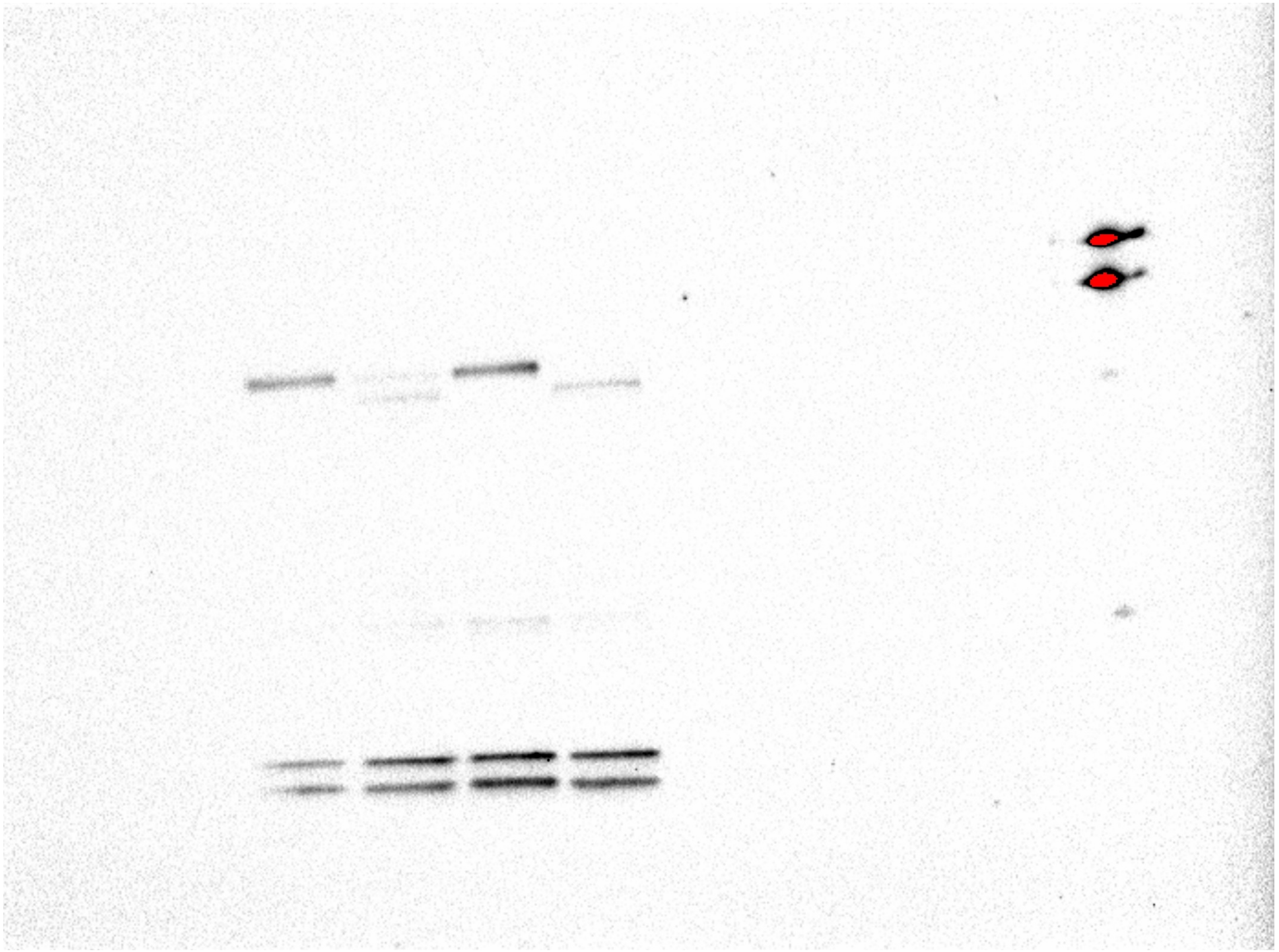

Figure 3D aHA

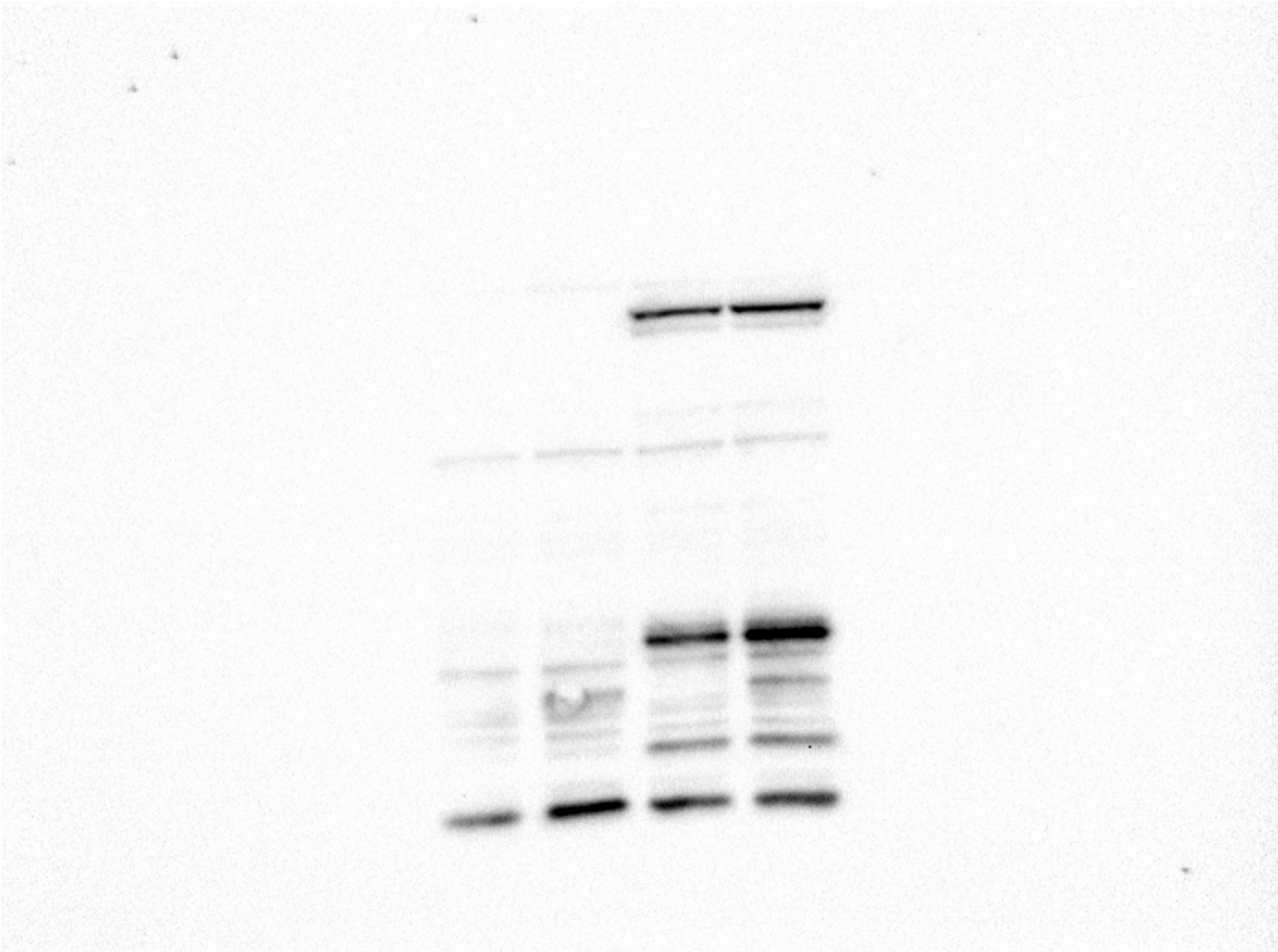

Figure 3D aGAPDH

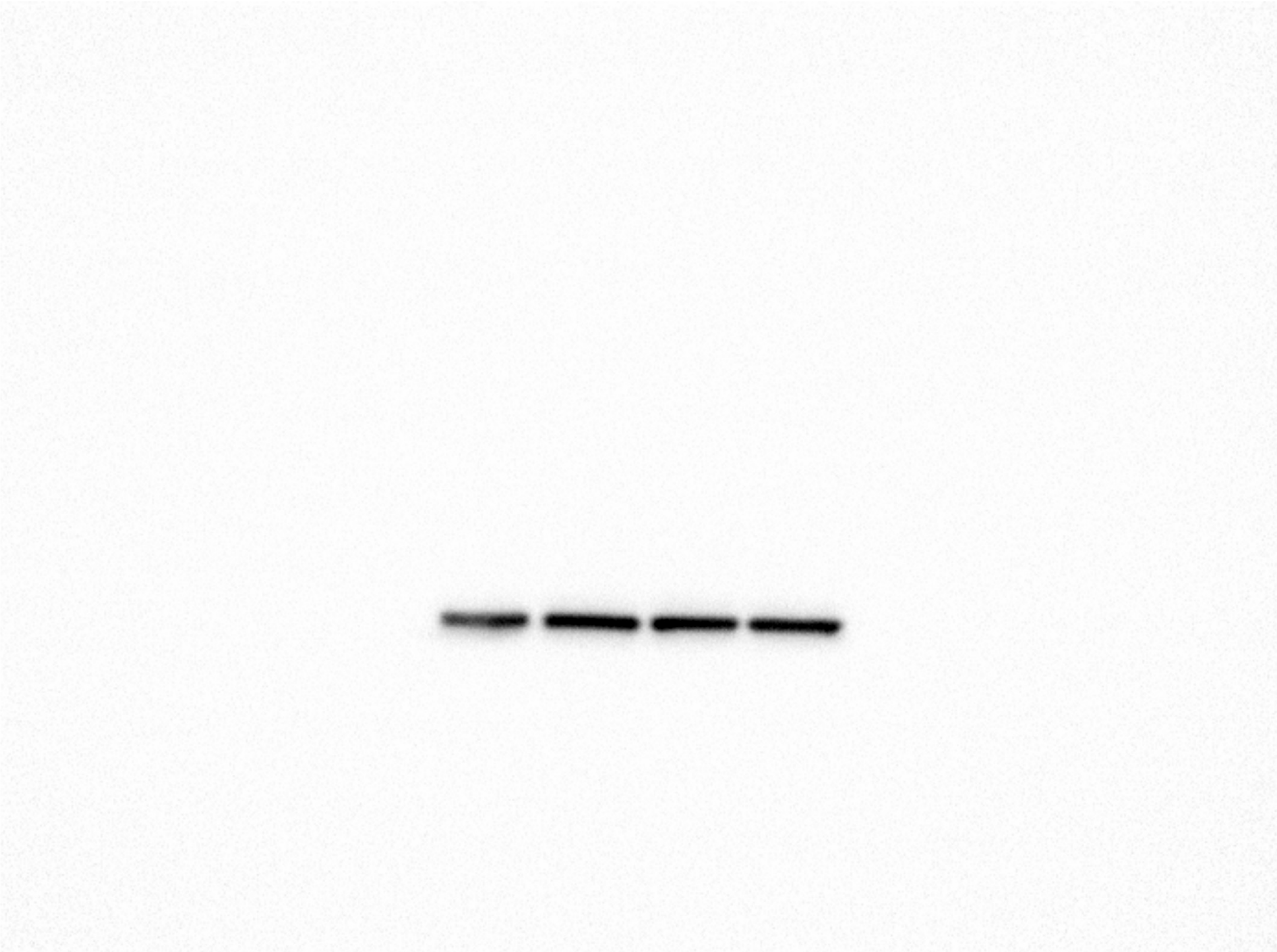

Figure 3E aV5

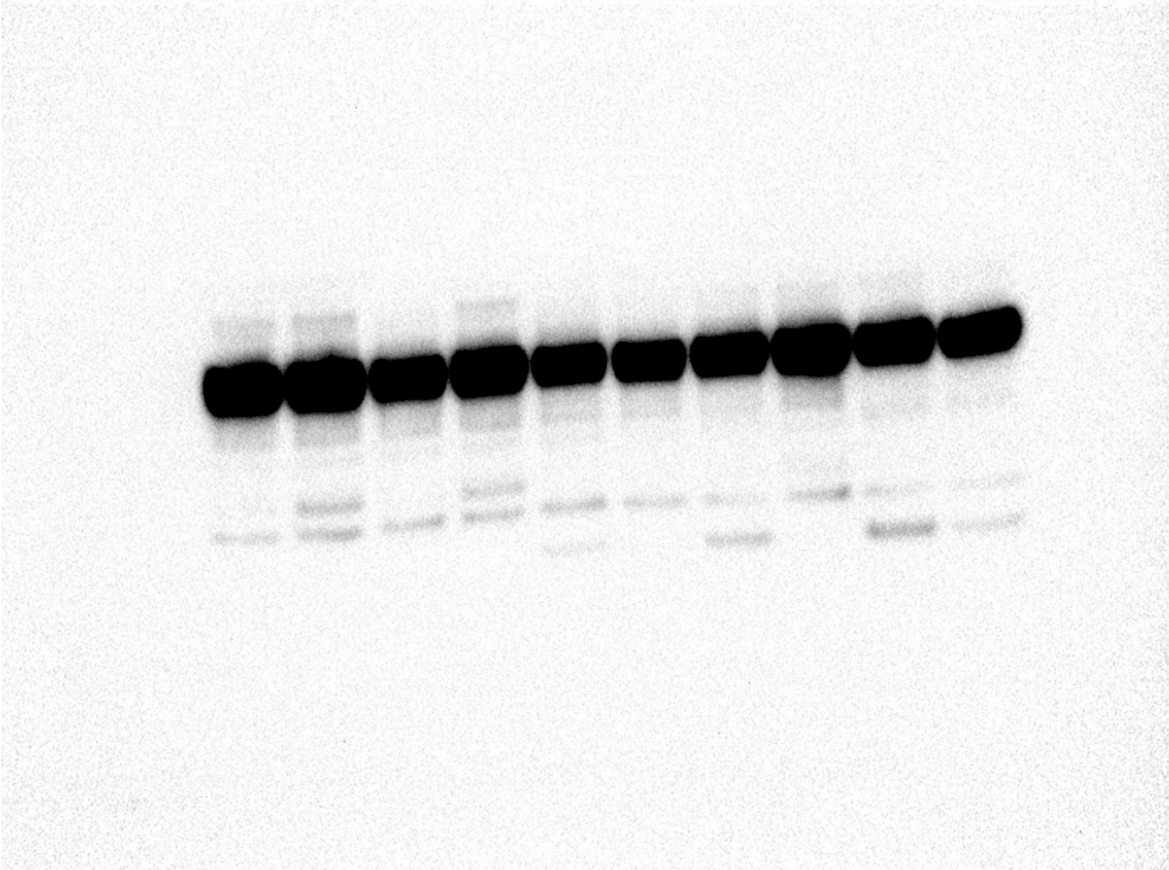

Figure 3E aMyc

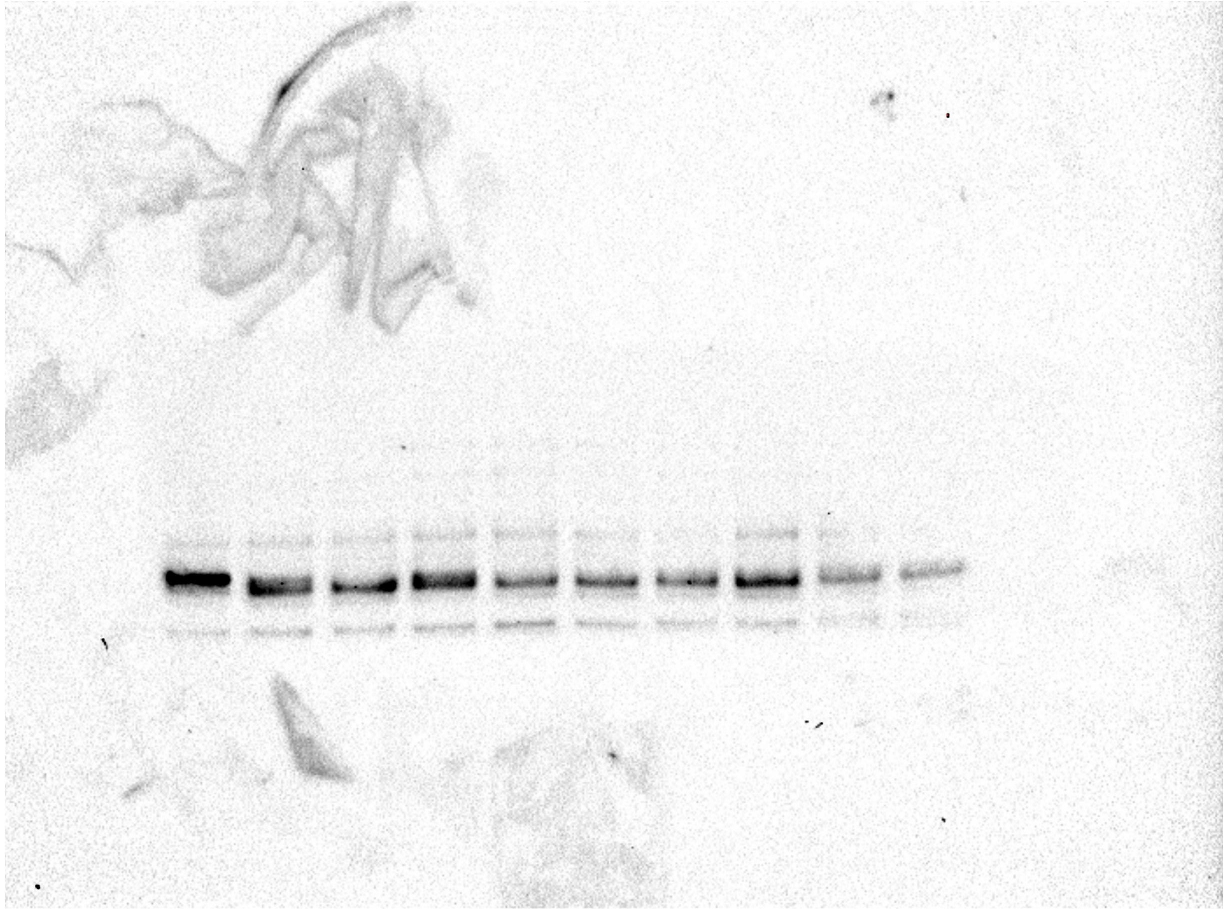

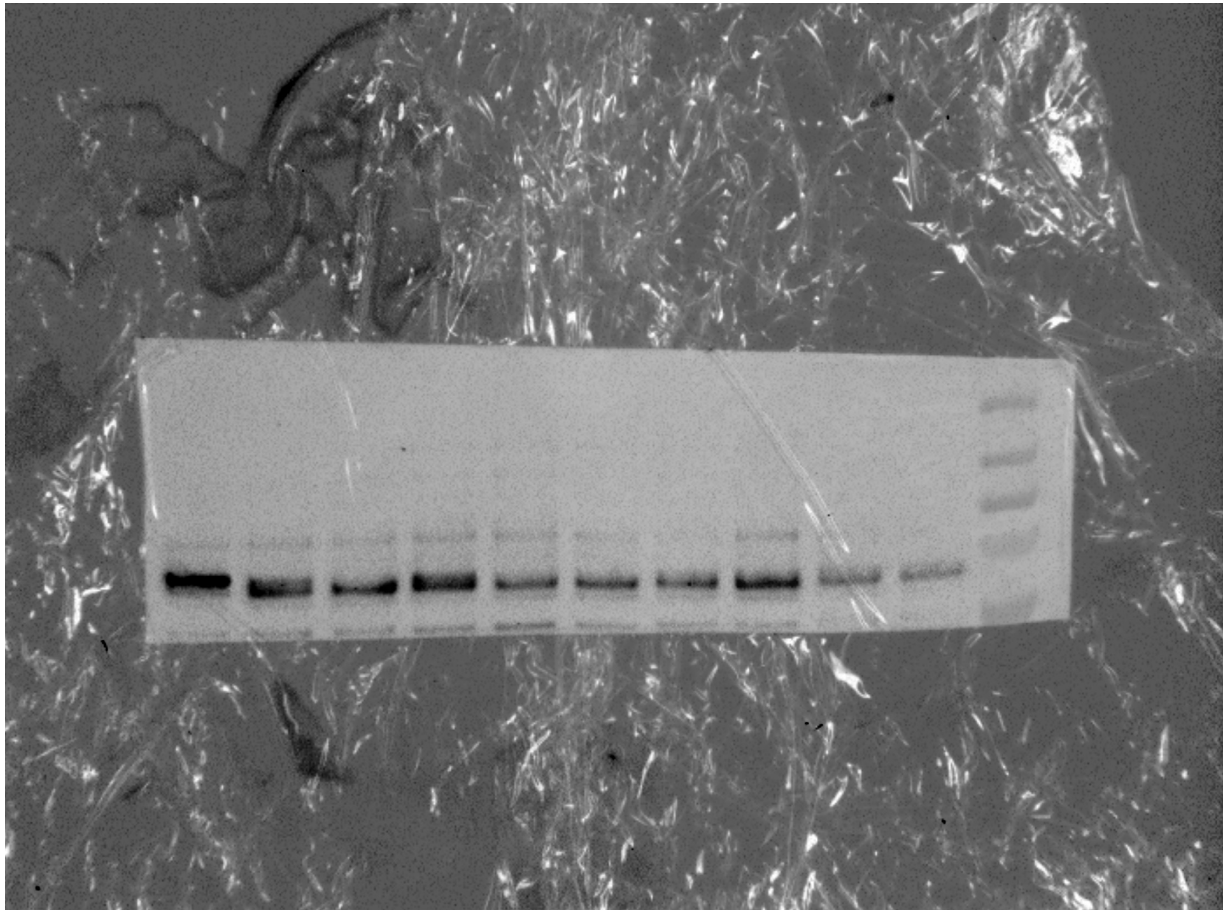

Figure 3E aHA

low

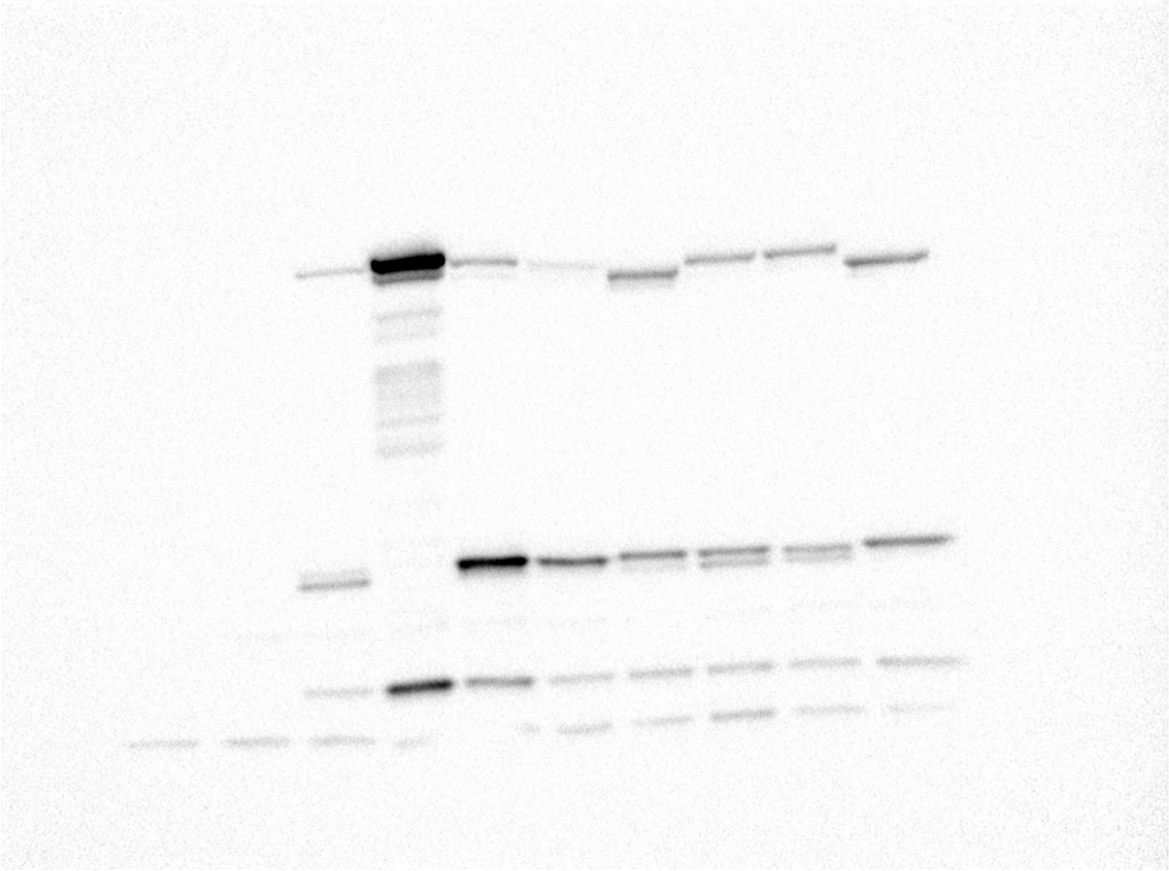

high

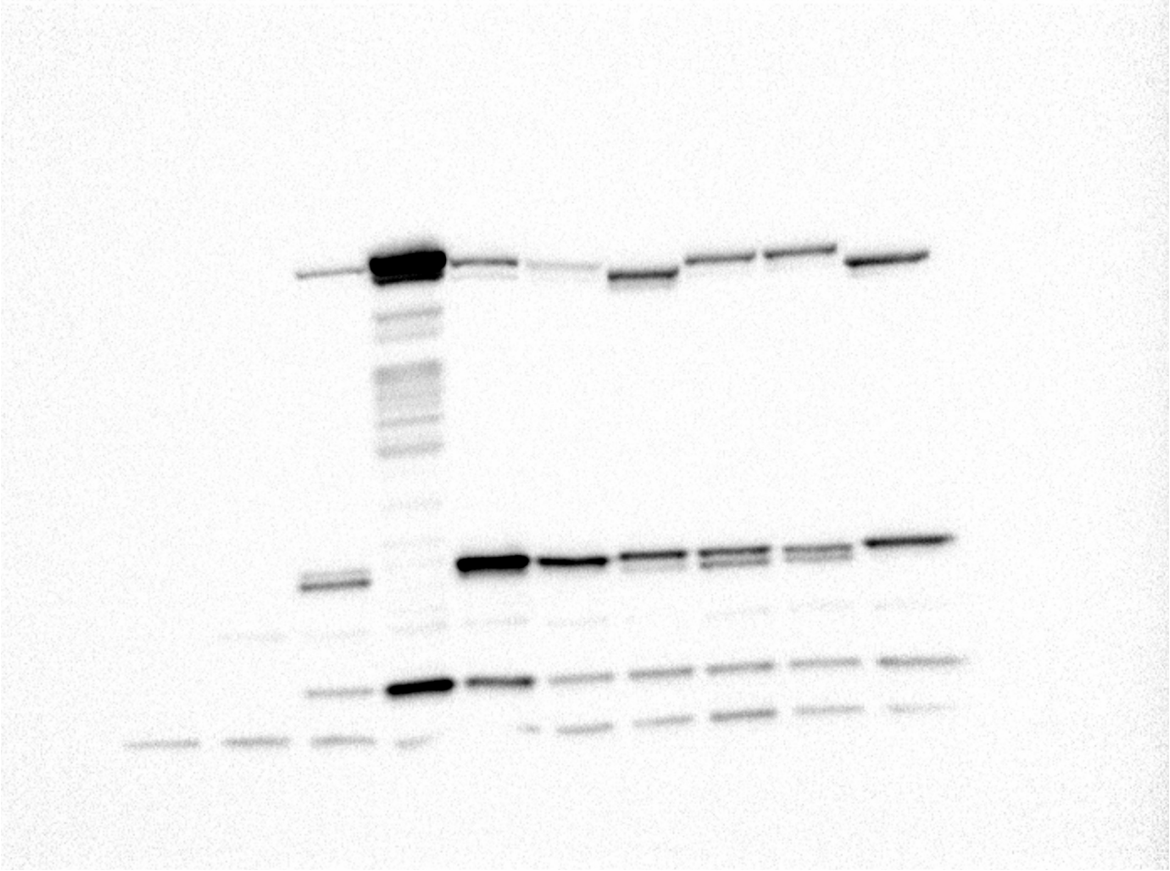

Figure 3E aGAPDH

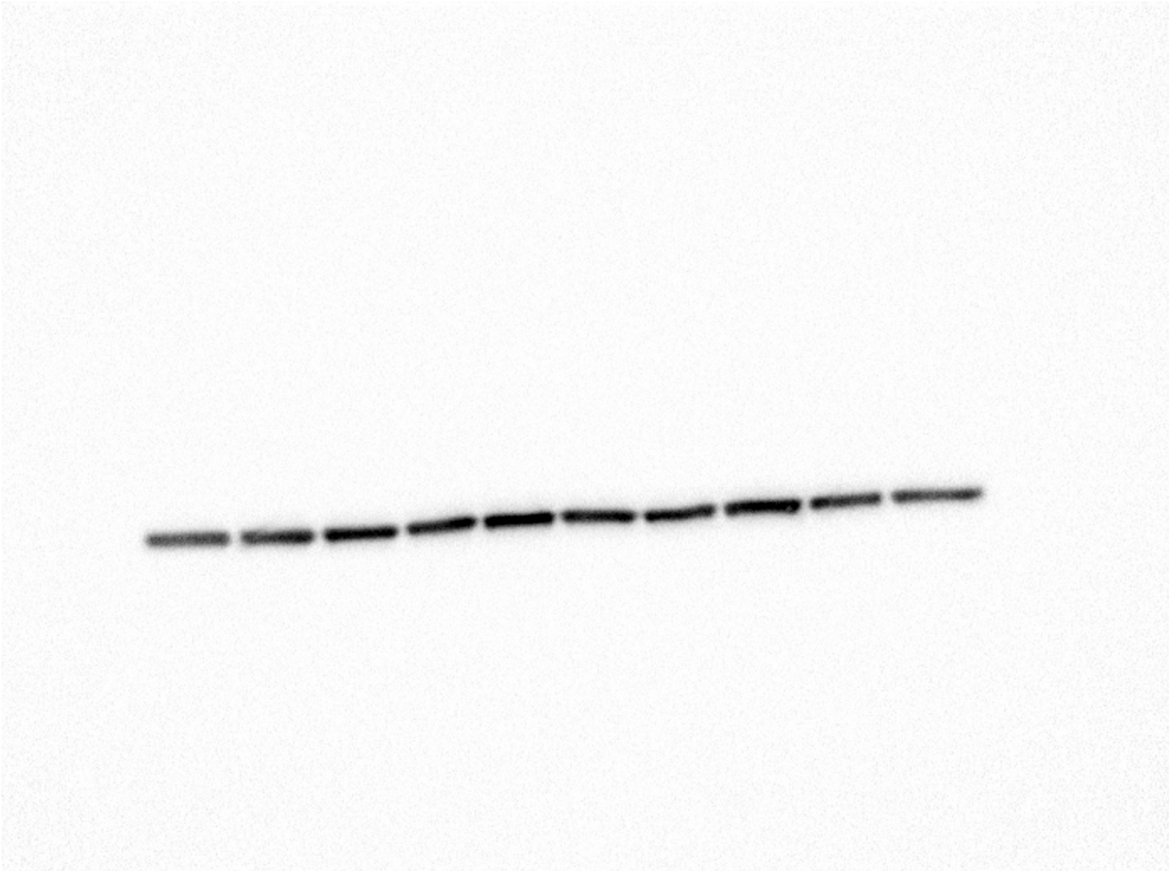

Figure 4C aFLAG

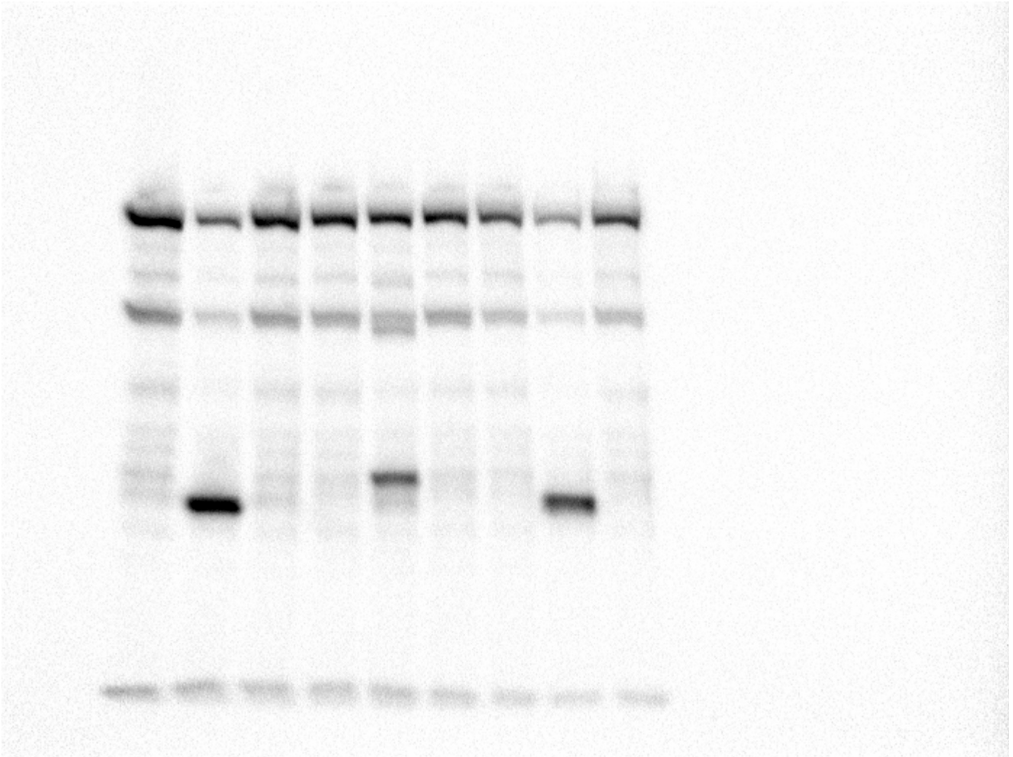

Figure 4C aHA

low

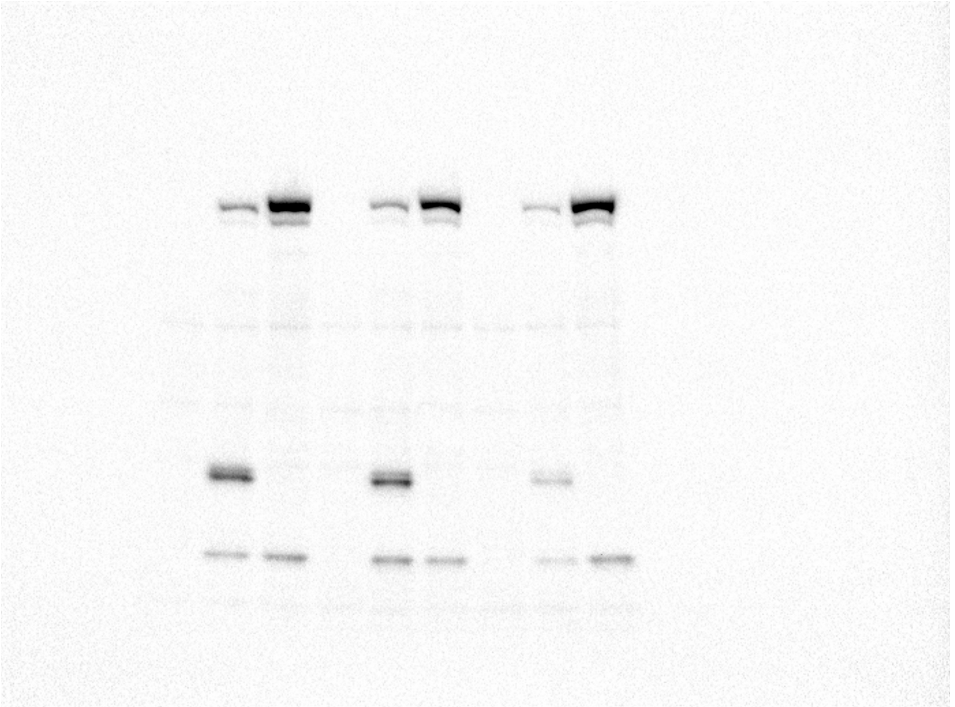

high

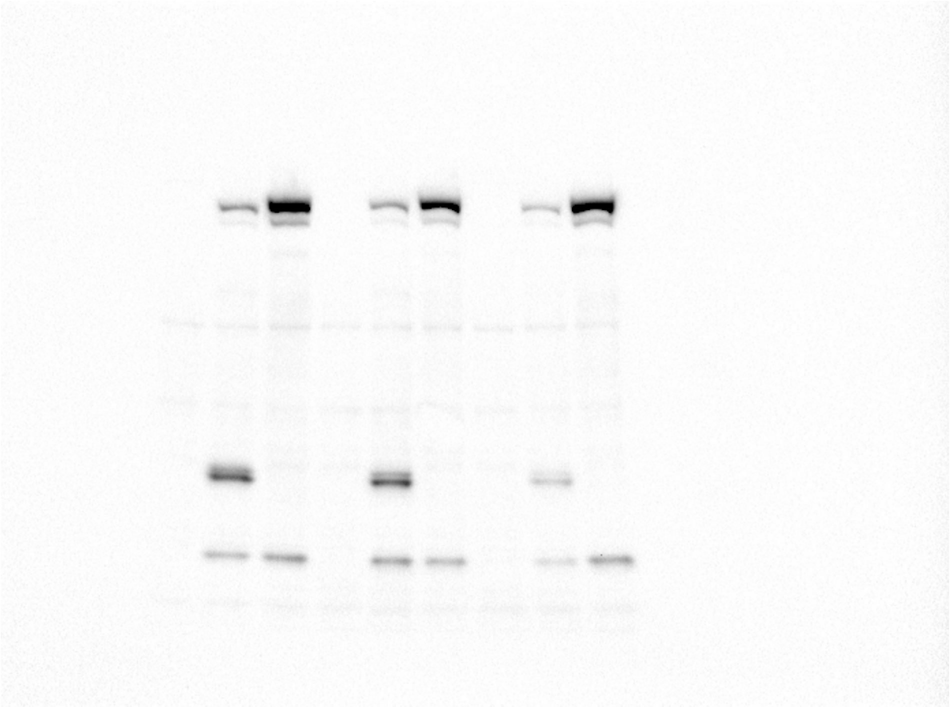

Figure 4C aGAPDH

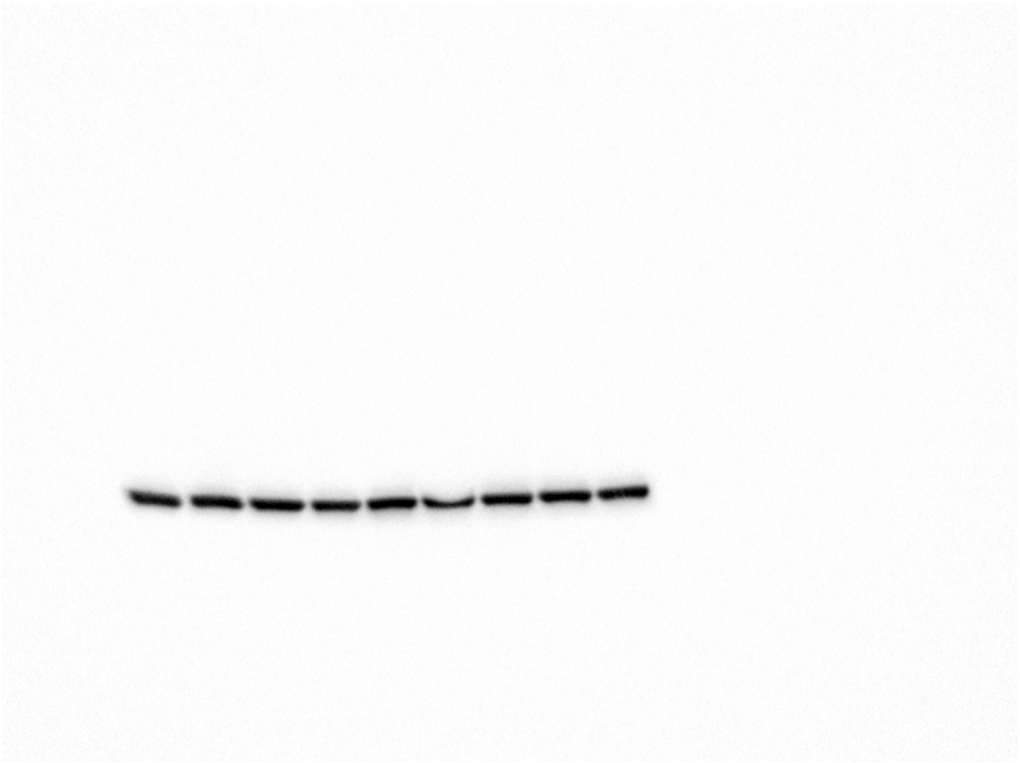

Figure 5B aFLAG

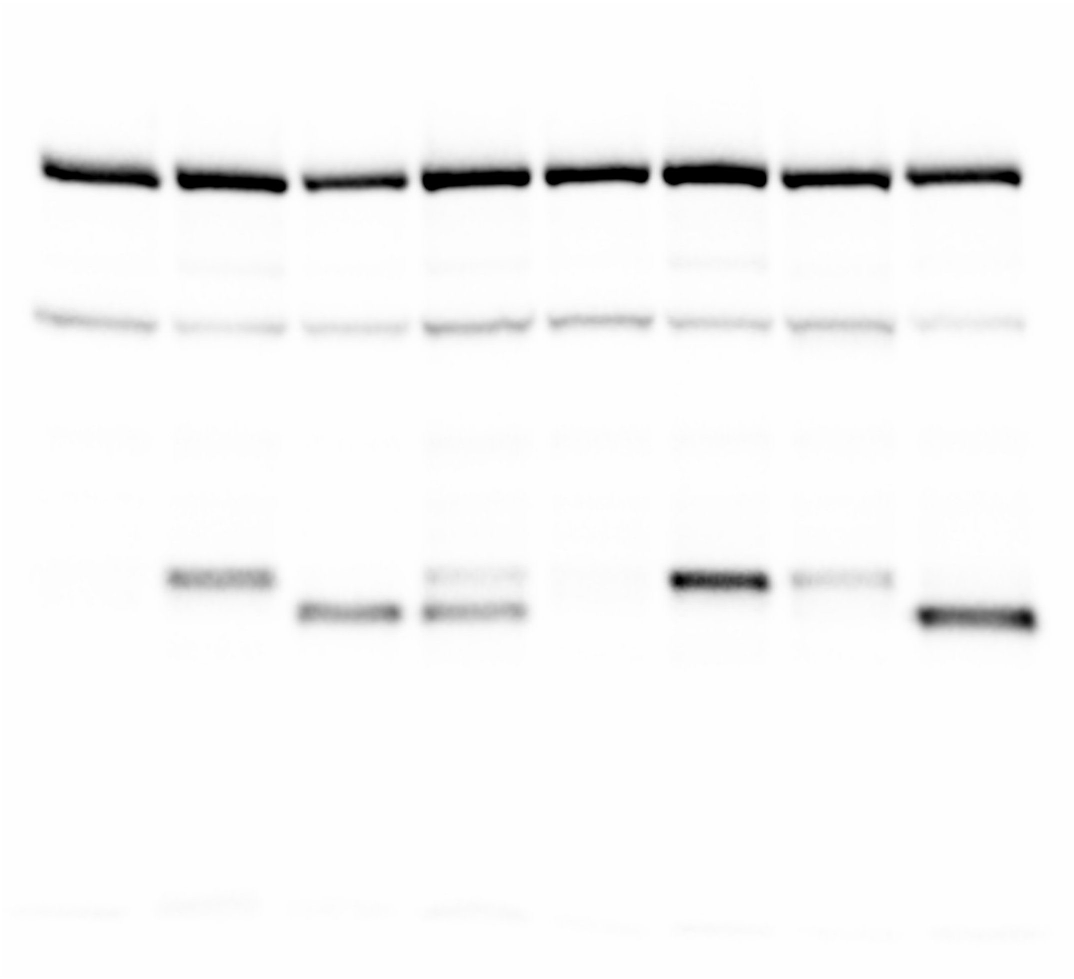

Figure 5B aHA

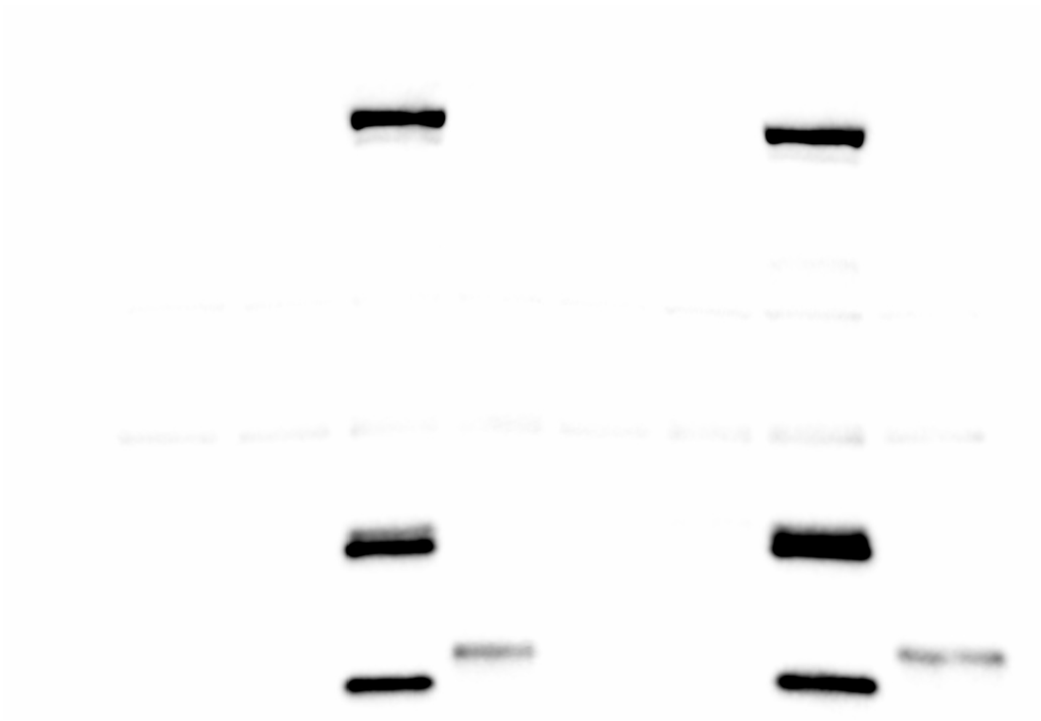

Figure 5B aGAPDH

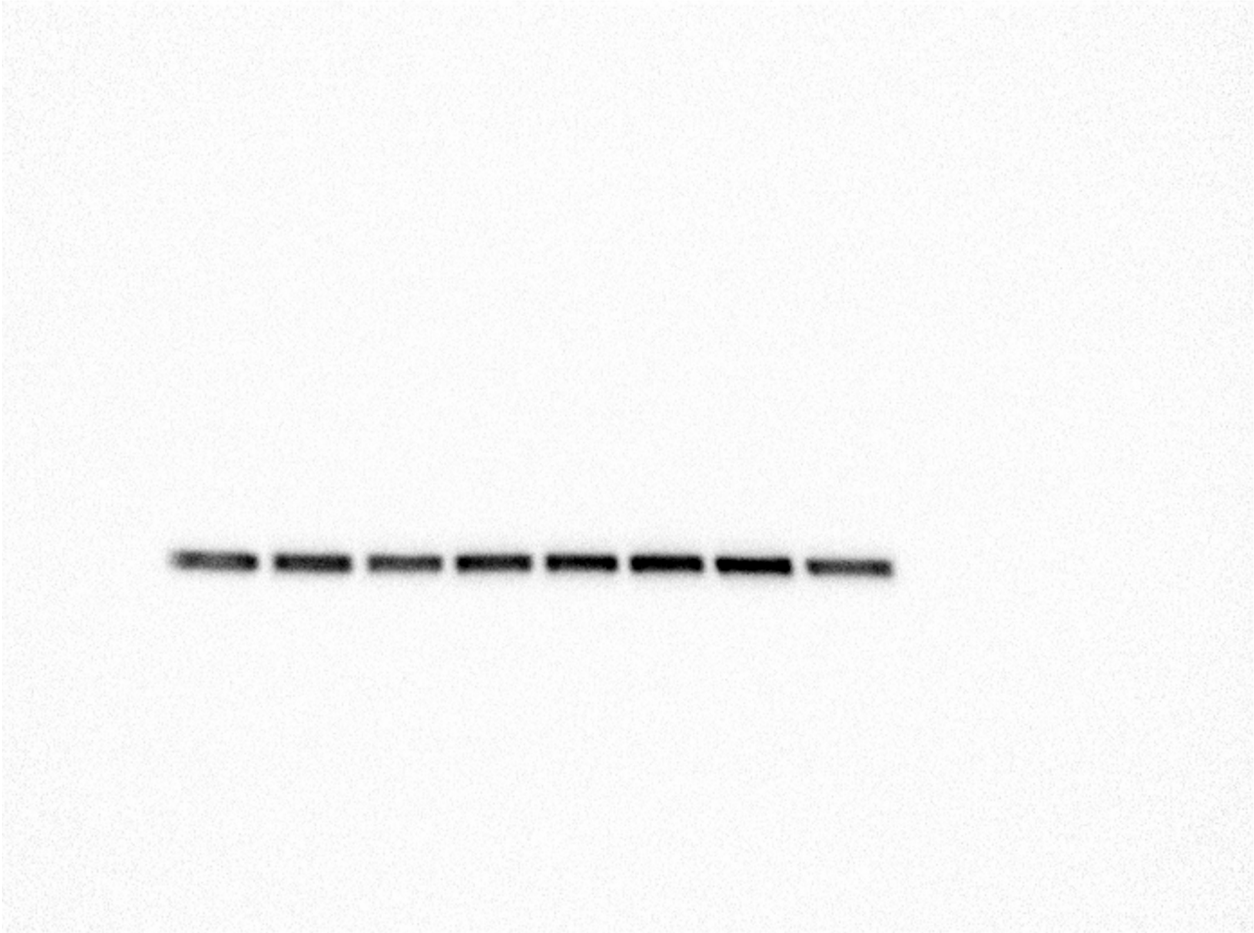

Figure 5C aV5

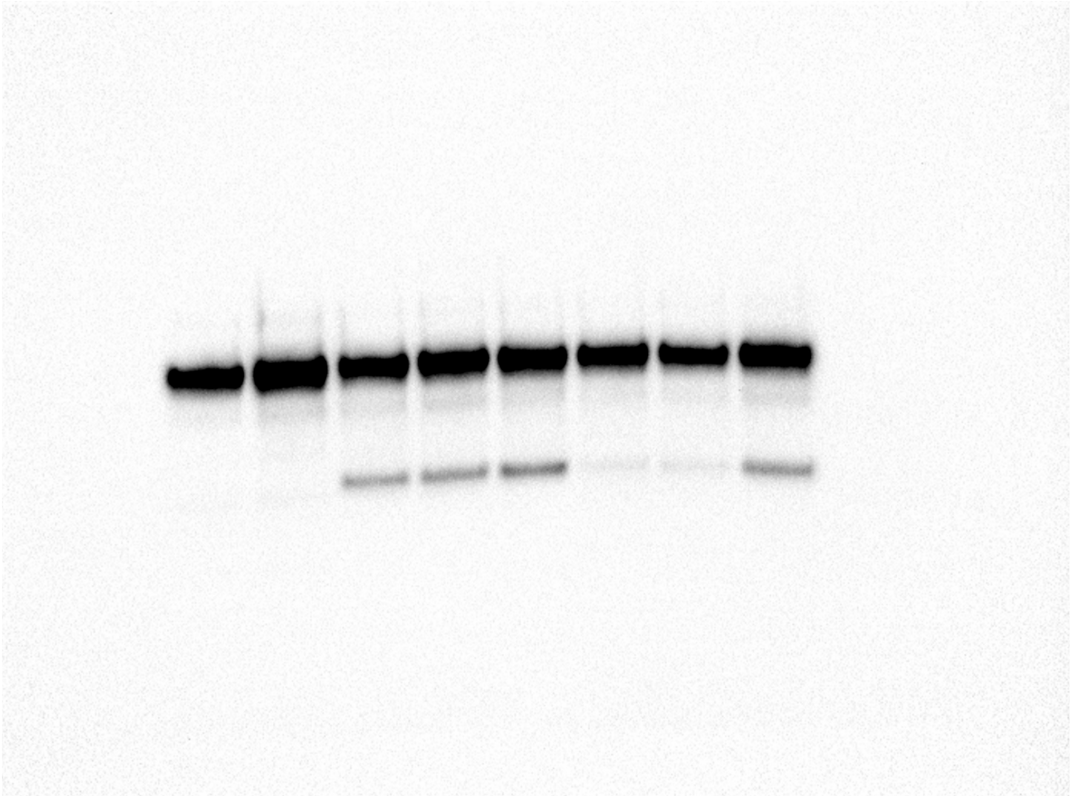

Figure 5C aMyc

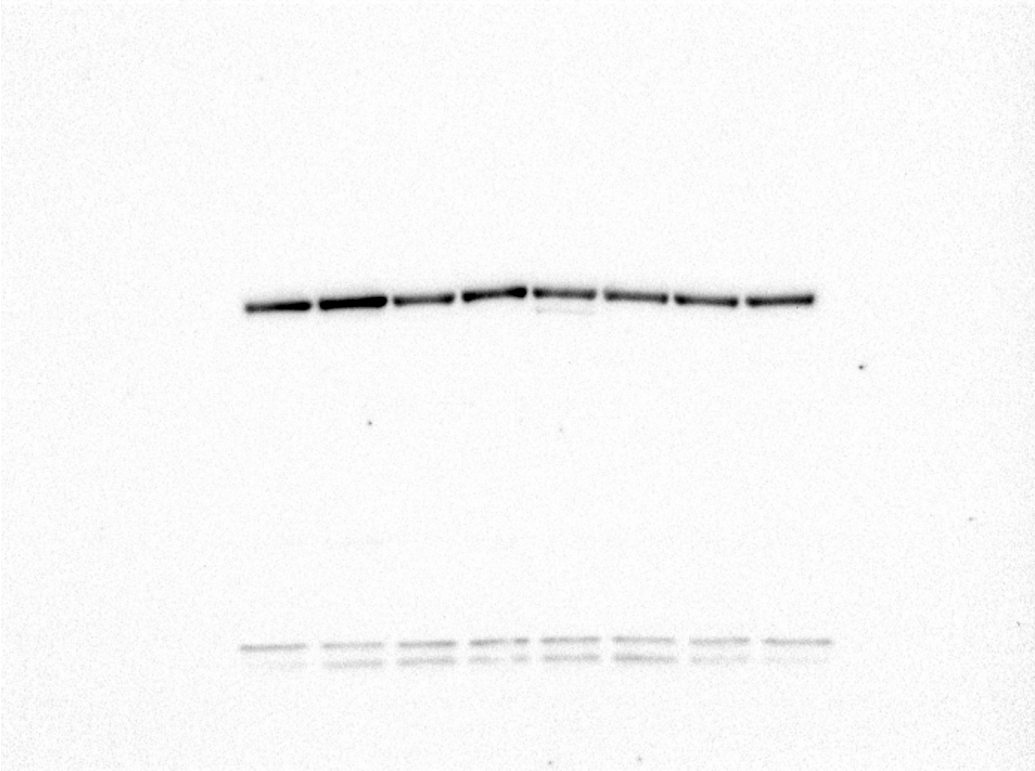

Figure 5C aHA

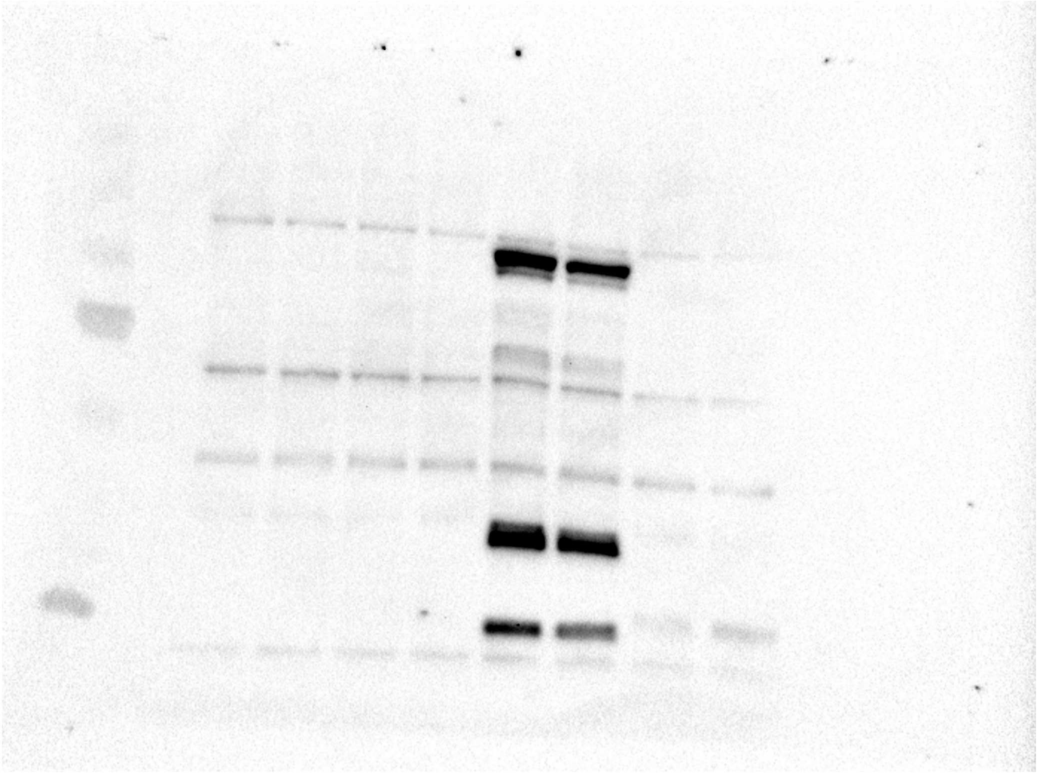

Figure 5C aGAPDH

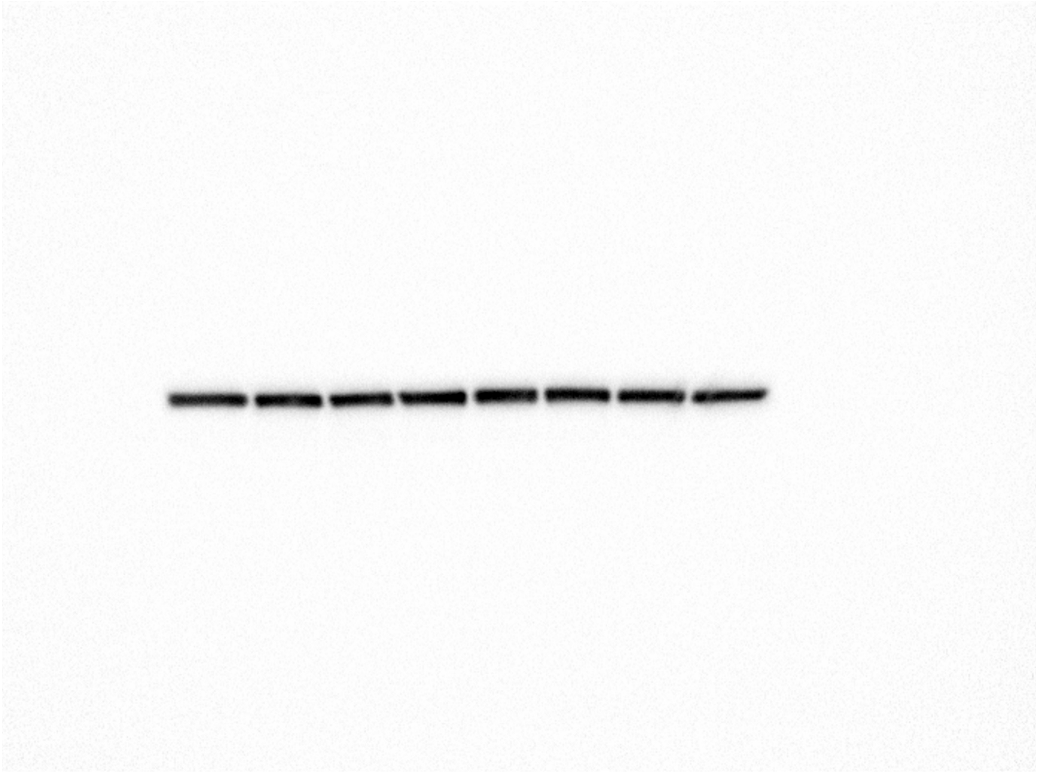

Figure S4-WT-Q37A-Q61A-aV5

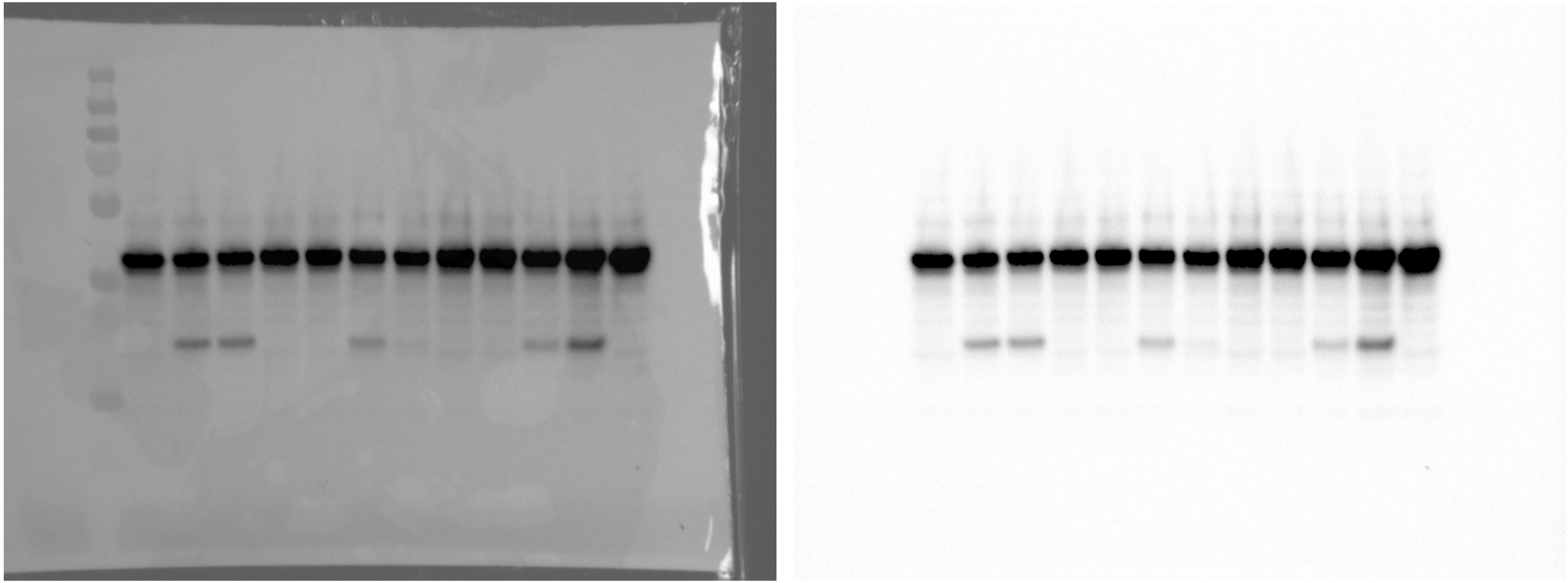

Figure S4-WT-Q37A-Q61A-aGAPDH

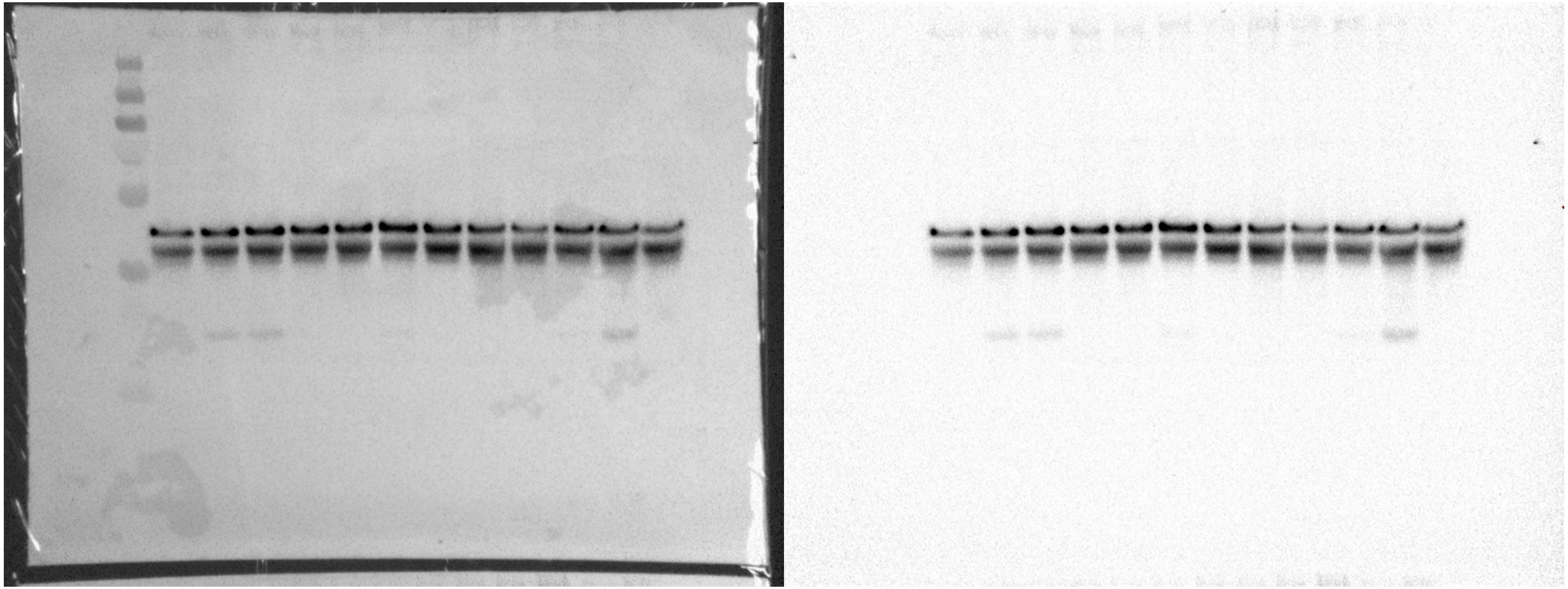

Figure S4-WT-Q61A-Q37A+Q61A-aV5

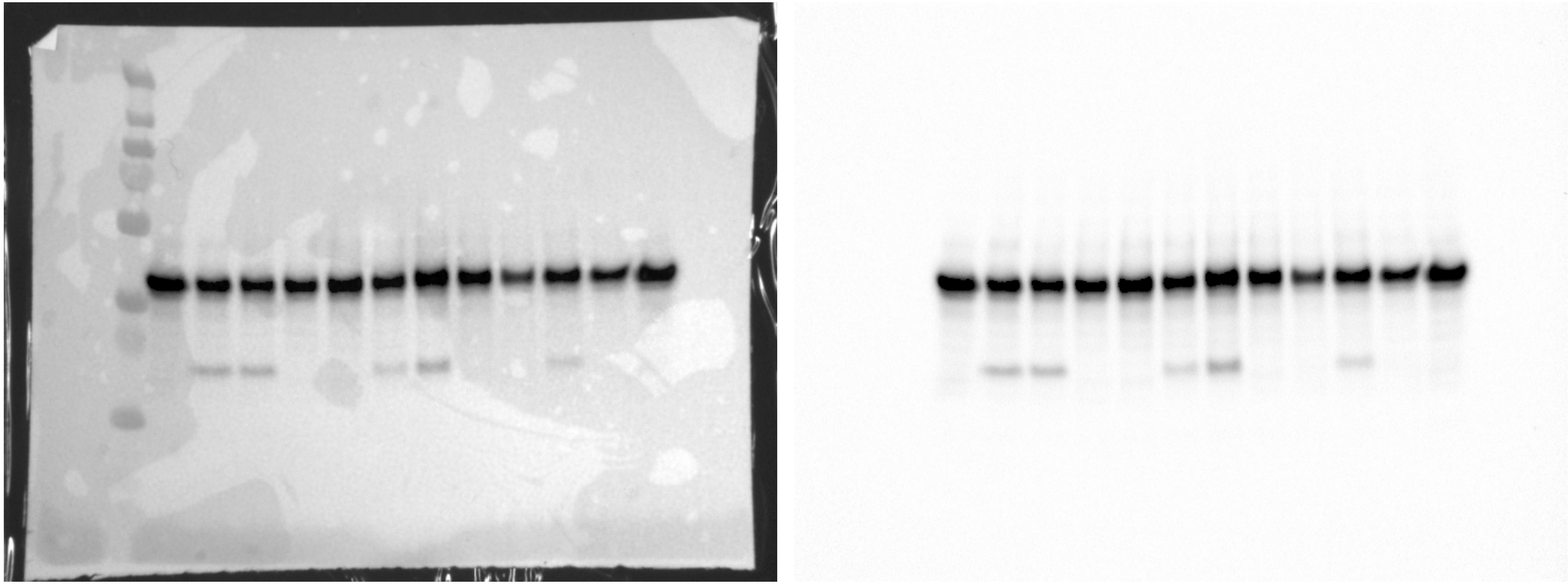

Figure S4-WT-Q61A-Q37A+Q61A-aGAPDH

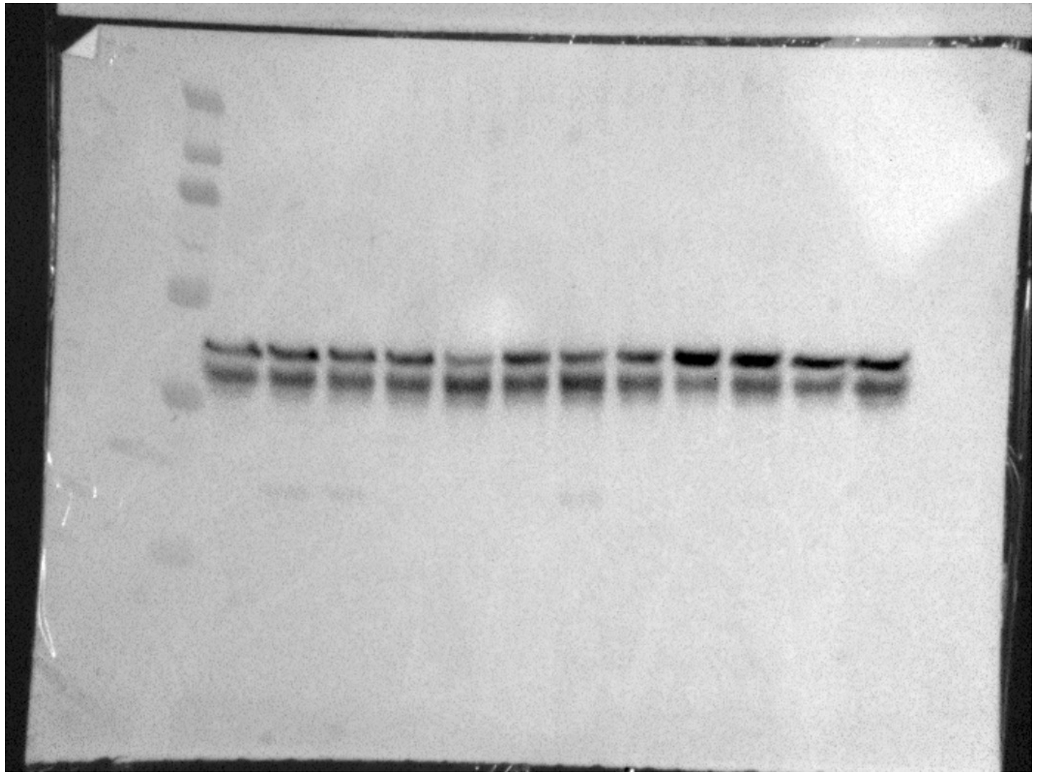

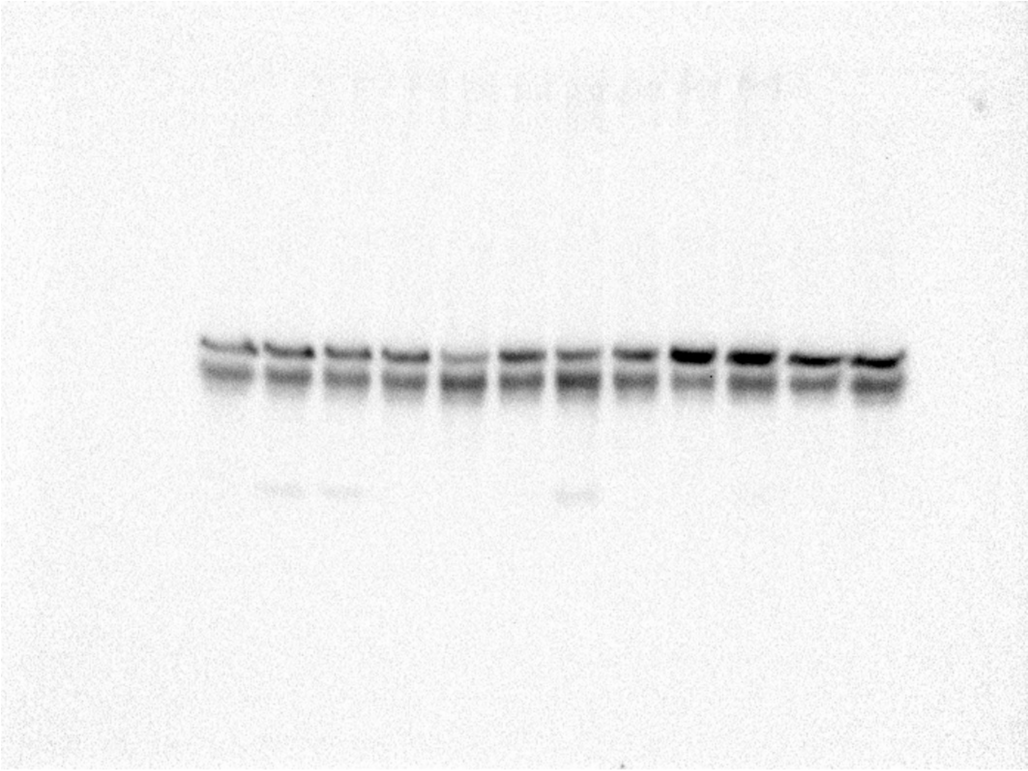

Figure S4-Q37A-Q61A-Q37A+Q61A-aV5

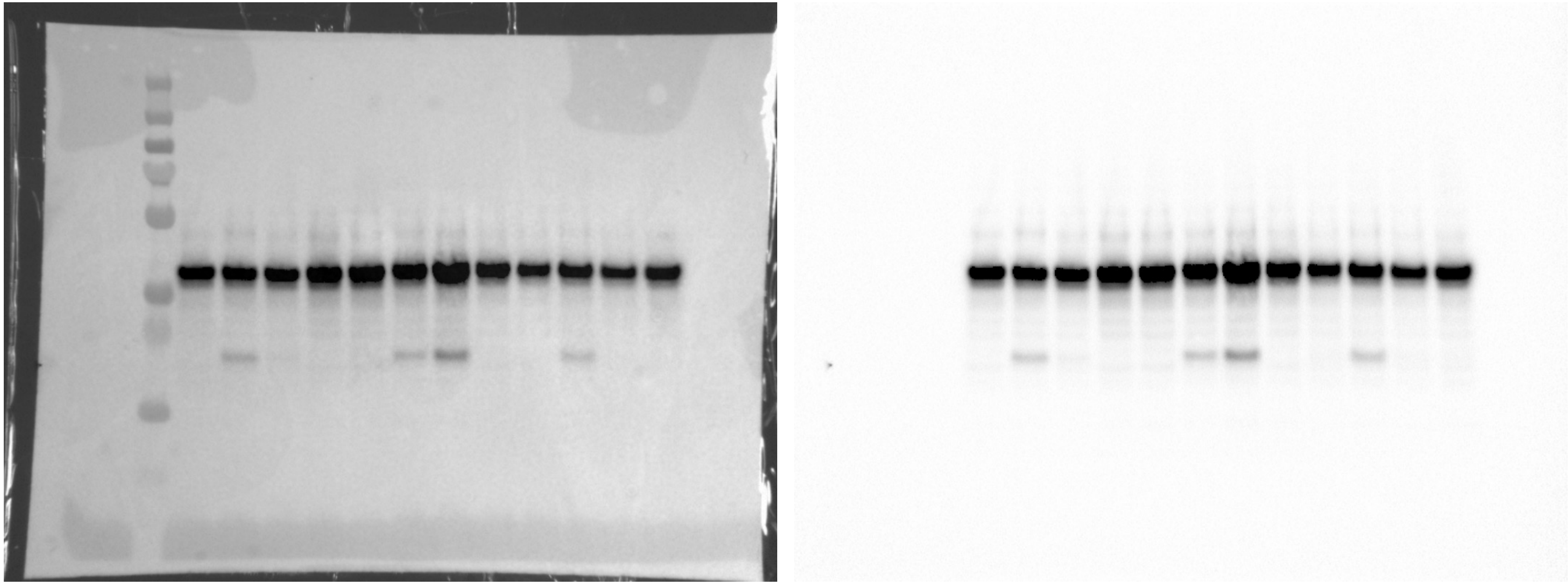

Figure S4-Q37A-Q61A-Q37A+Q61A-aGAPDH

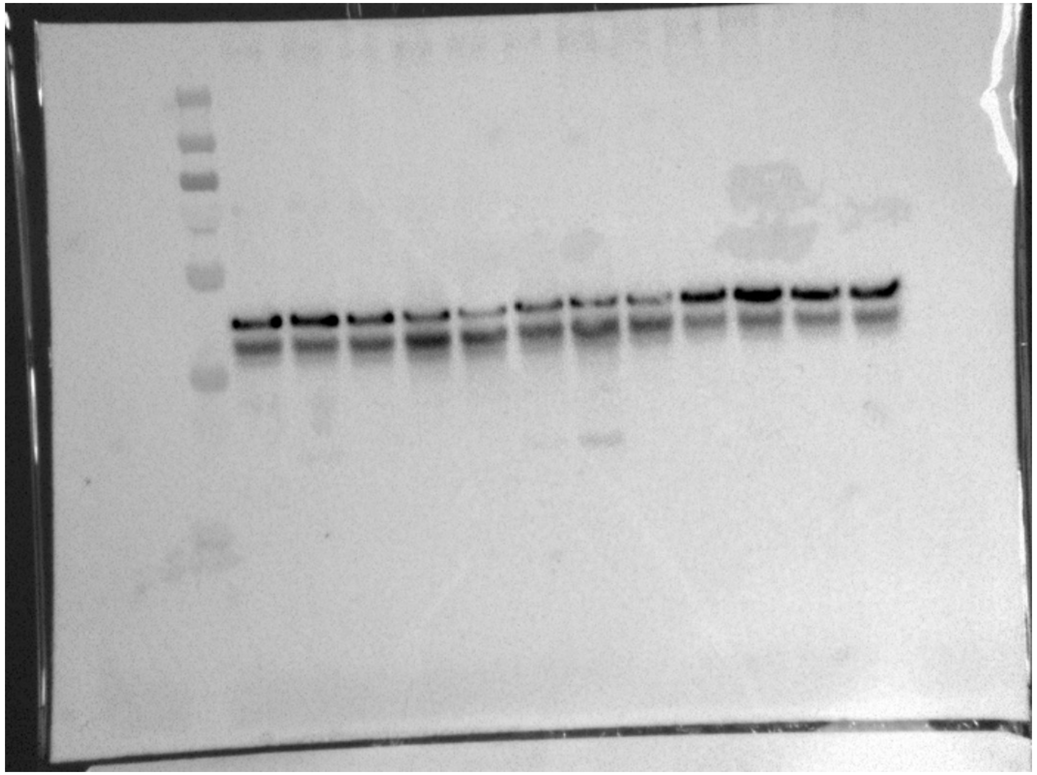

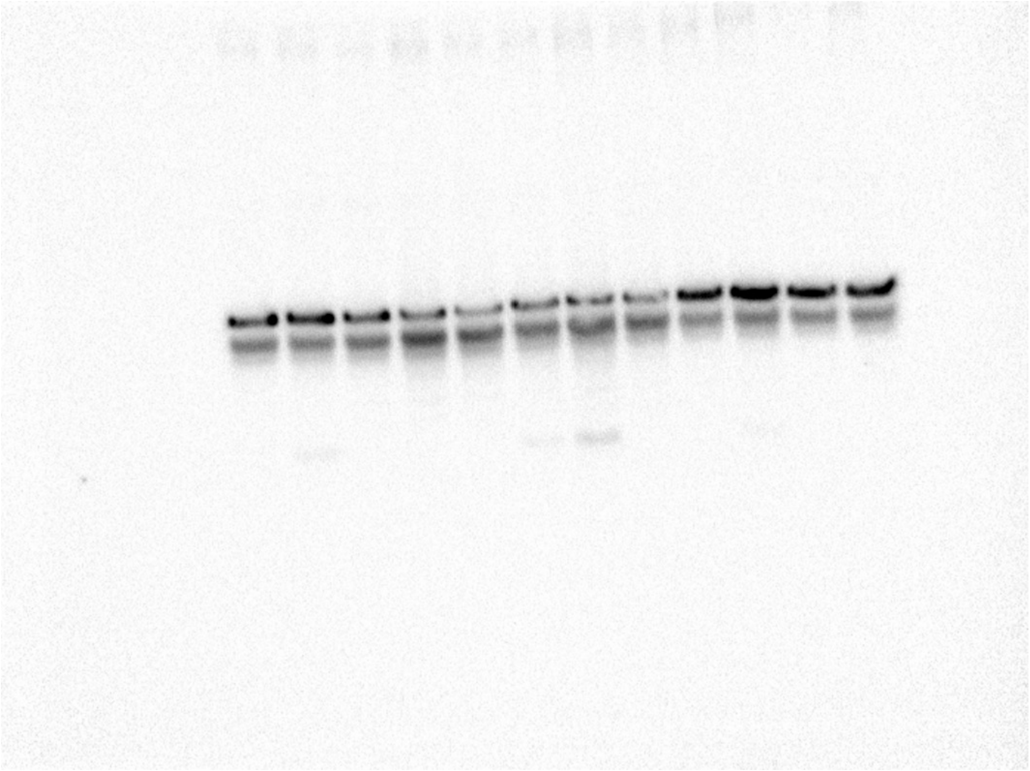

Figure S3 aV5

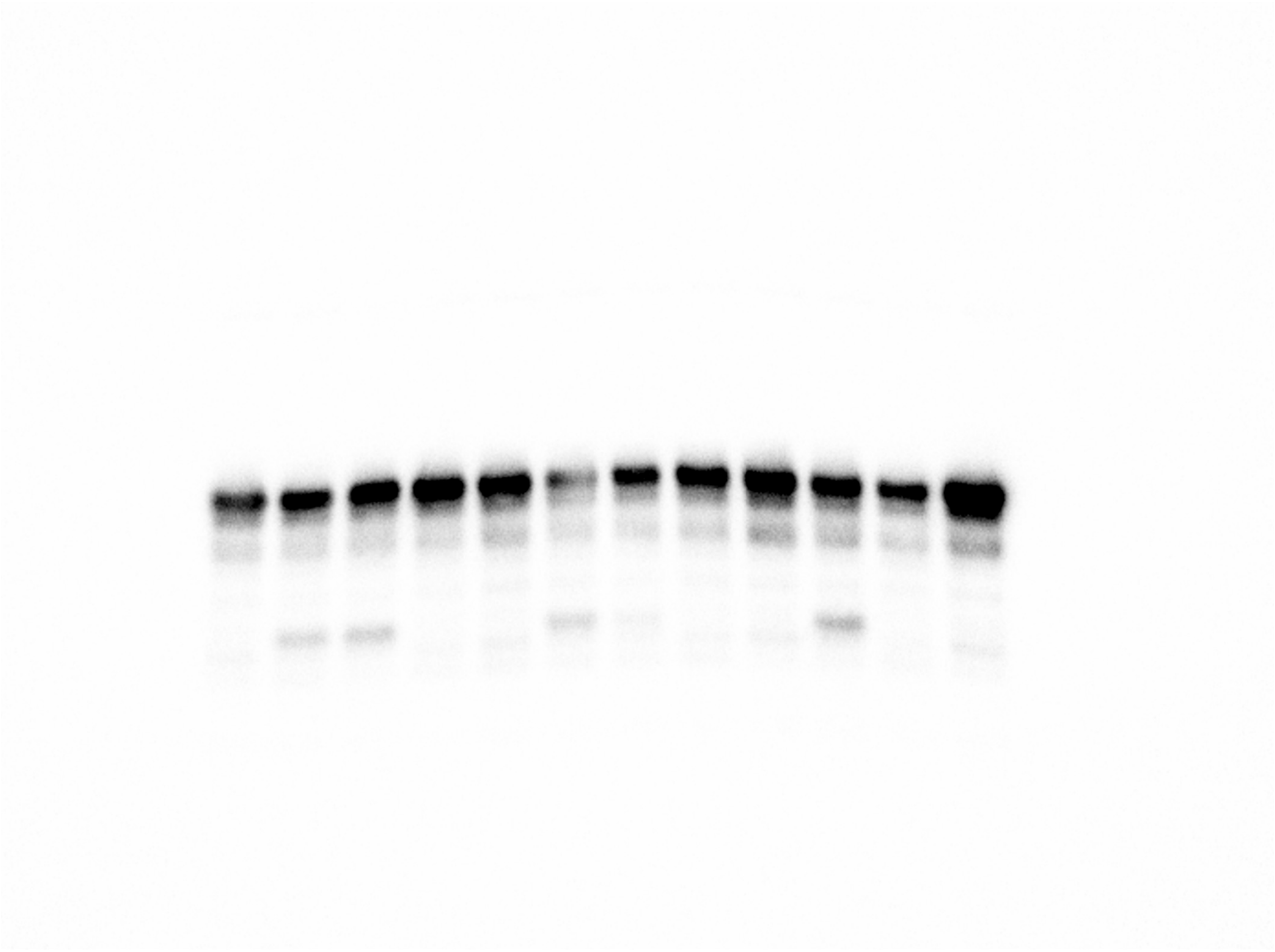

Figure S3 aMyc

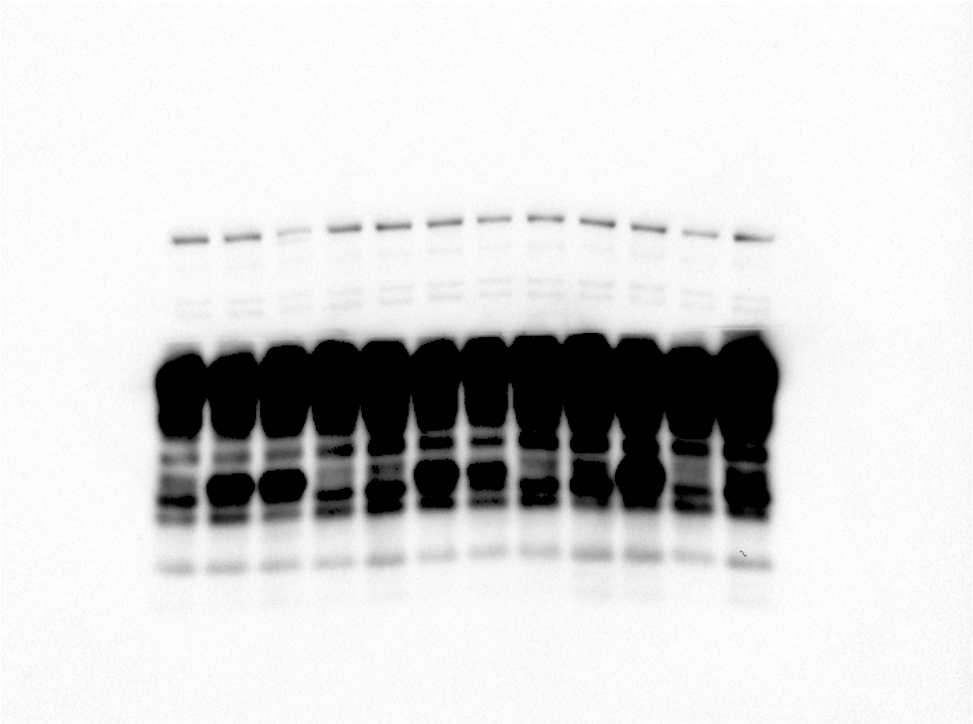

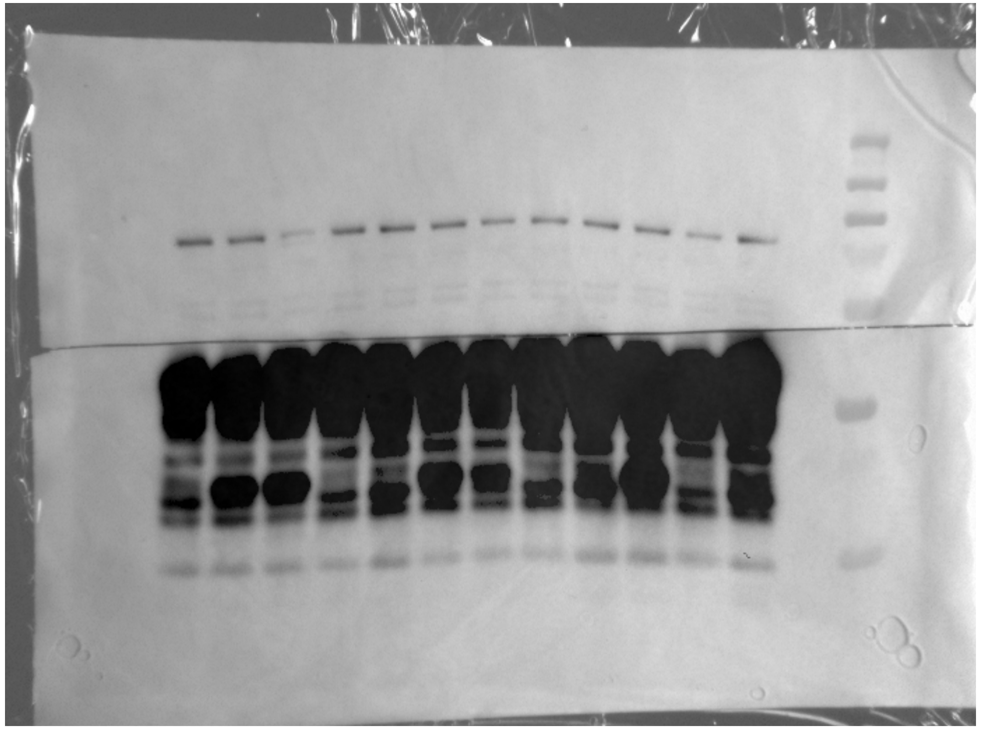

Figure S3 aHA

low

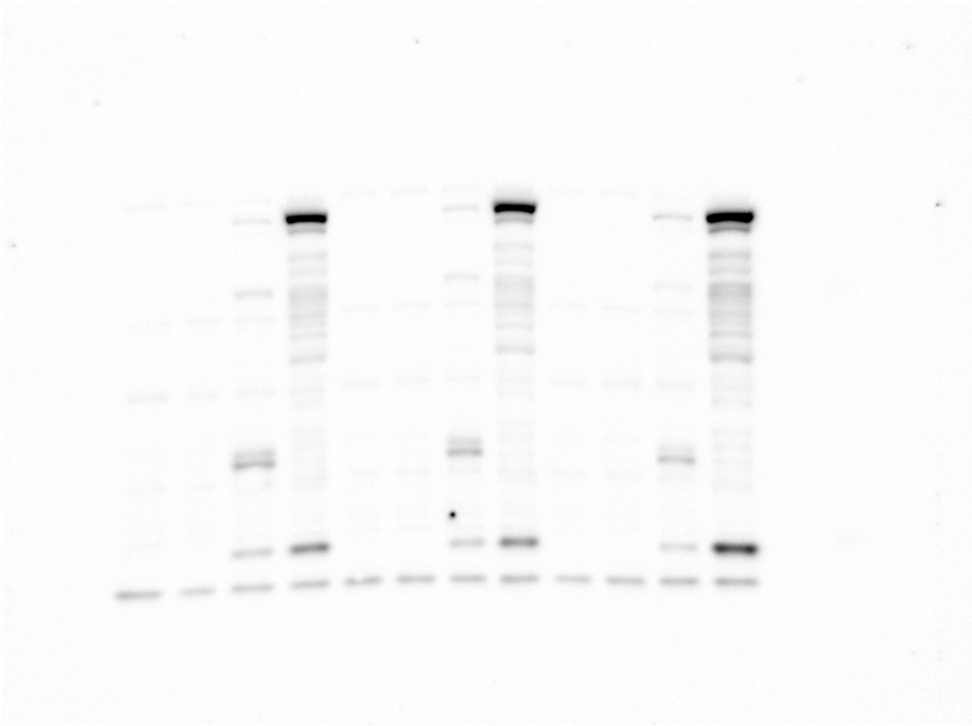

high

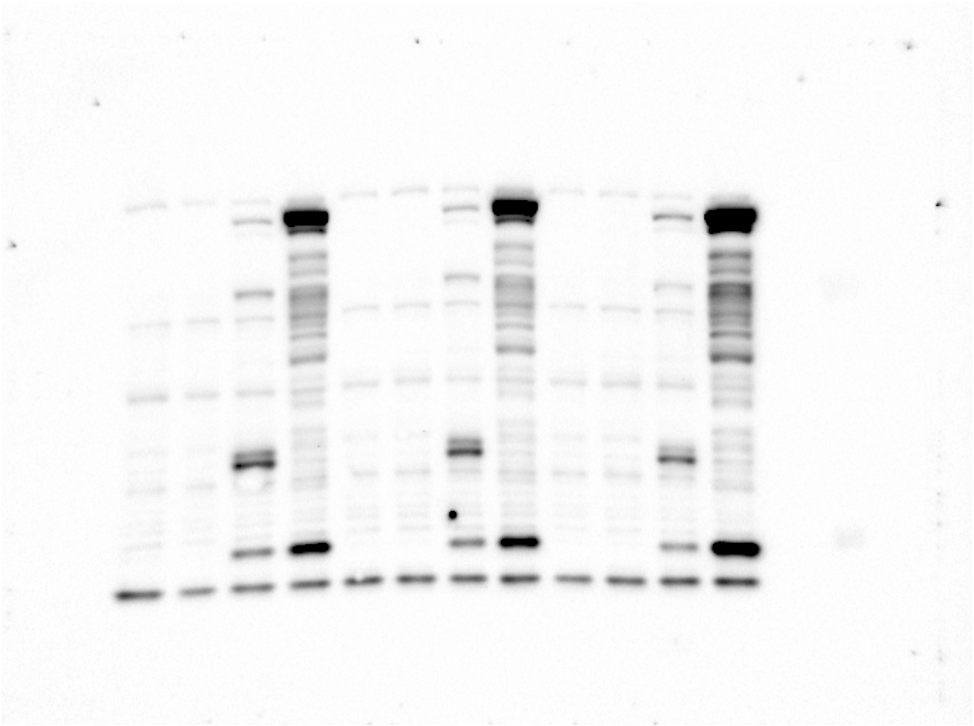

Figure S3 aGAPDH

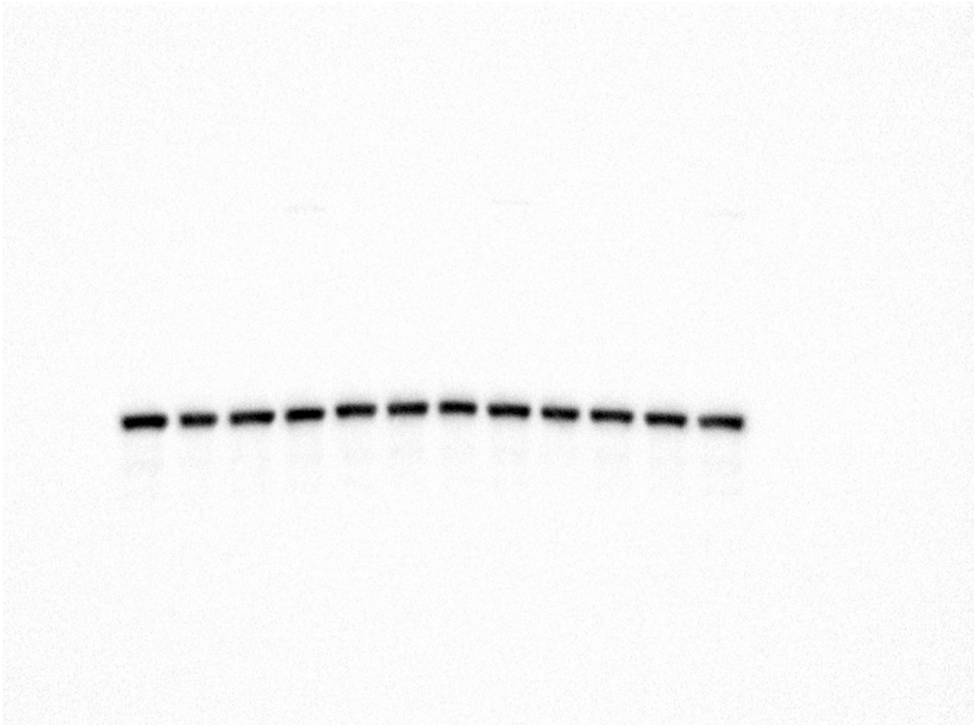

Figure S5 aV5

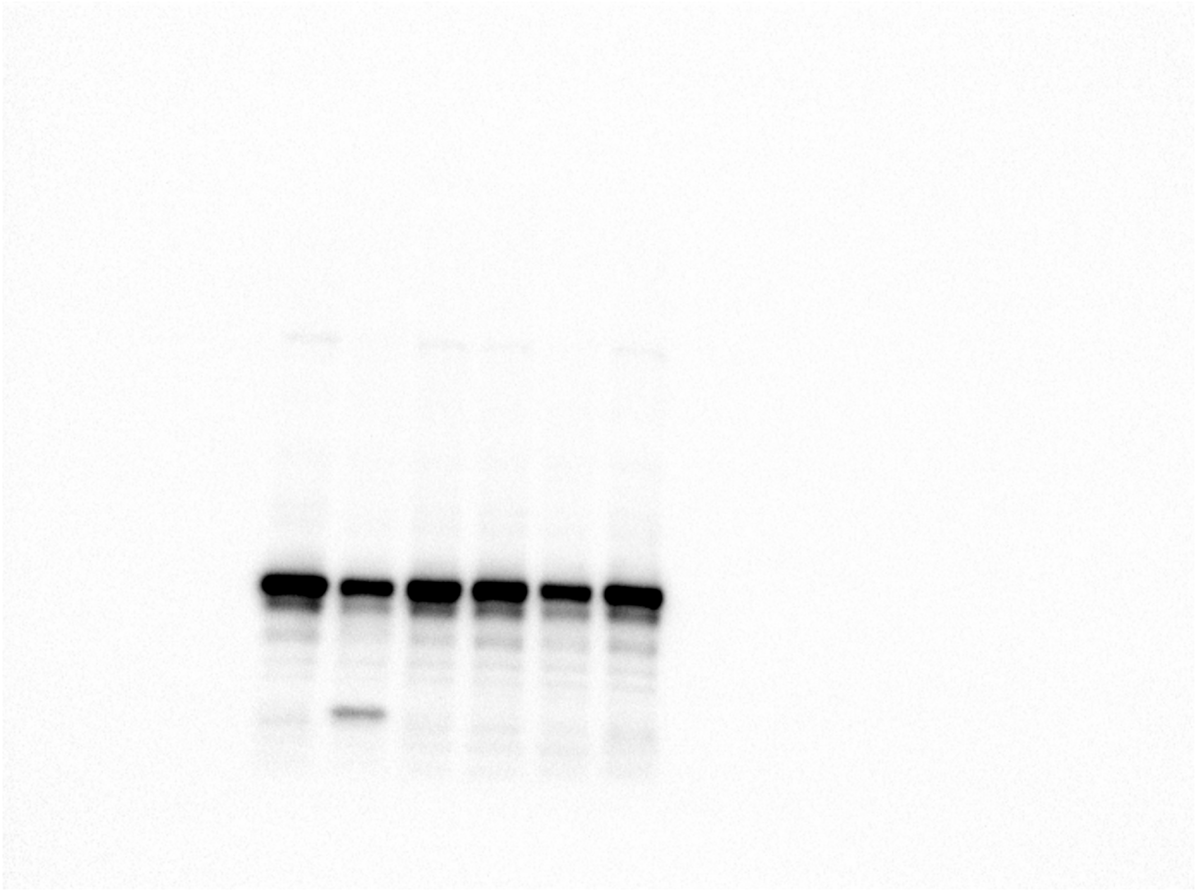

Figure S5 aMyc

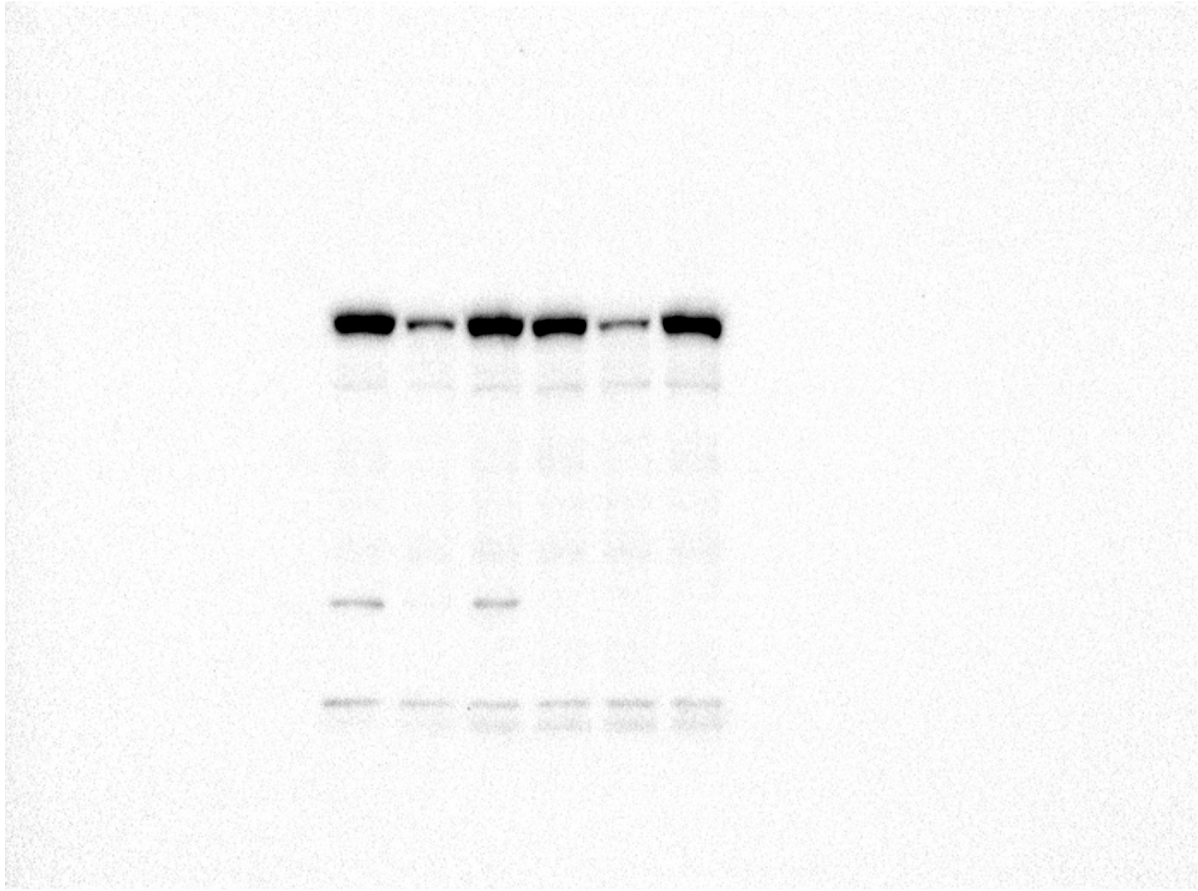

Figure S5 aHA

low

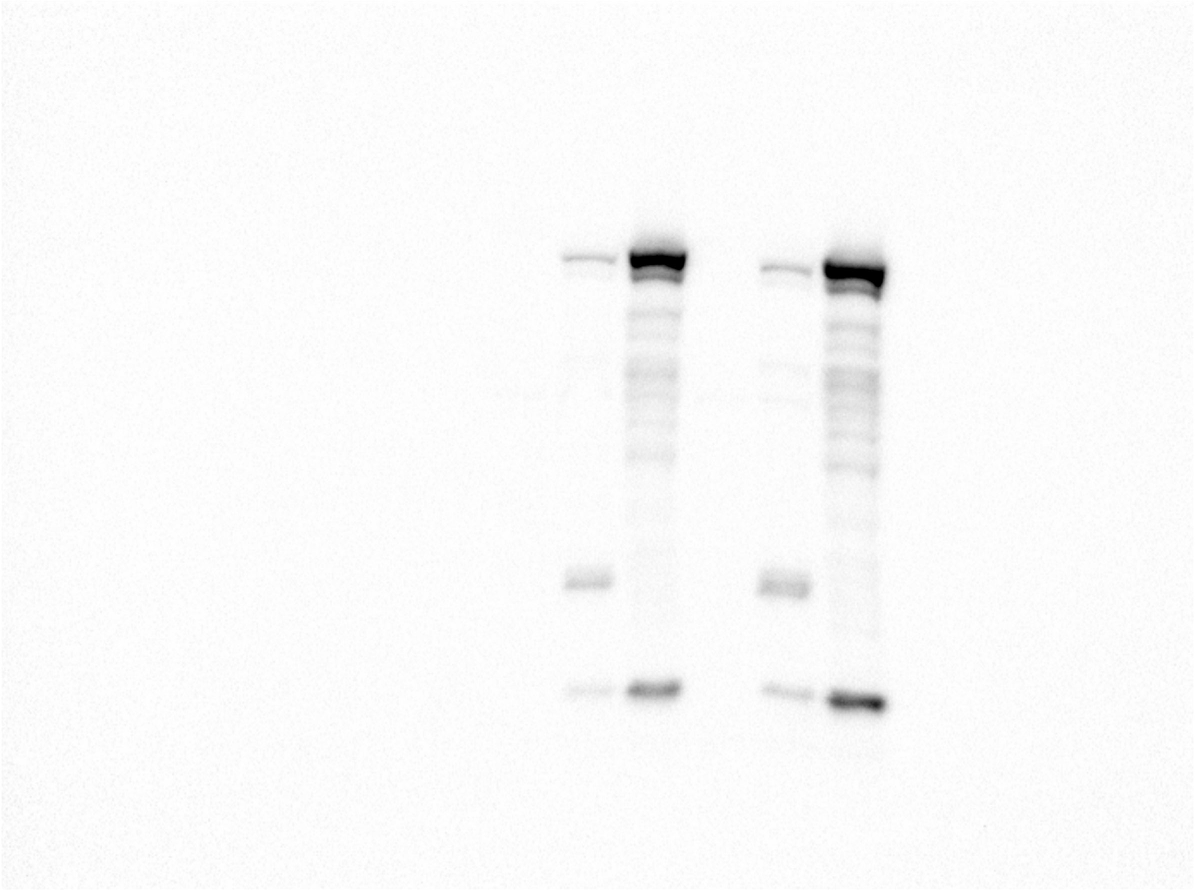

high

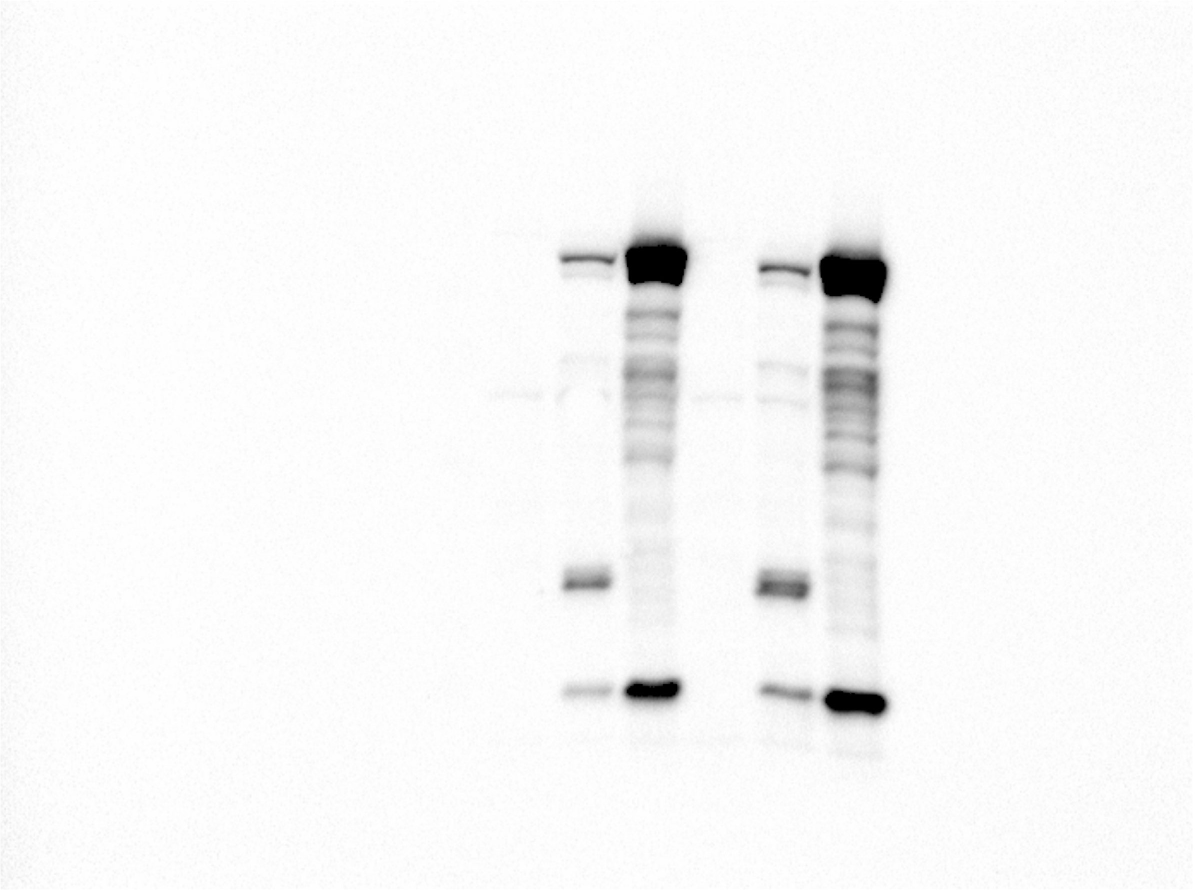

Figure S5 aGAPDH

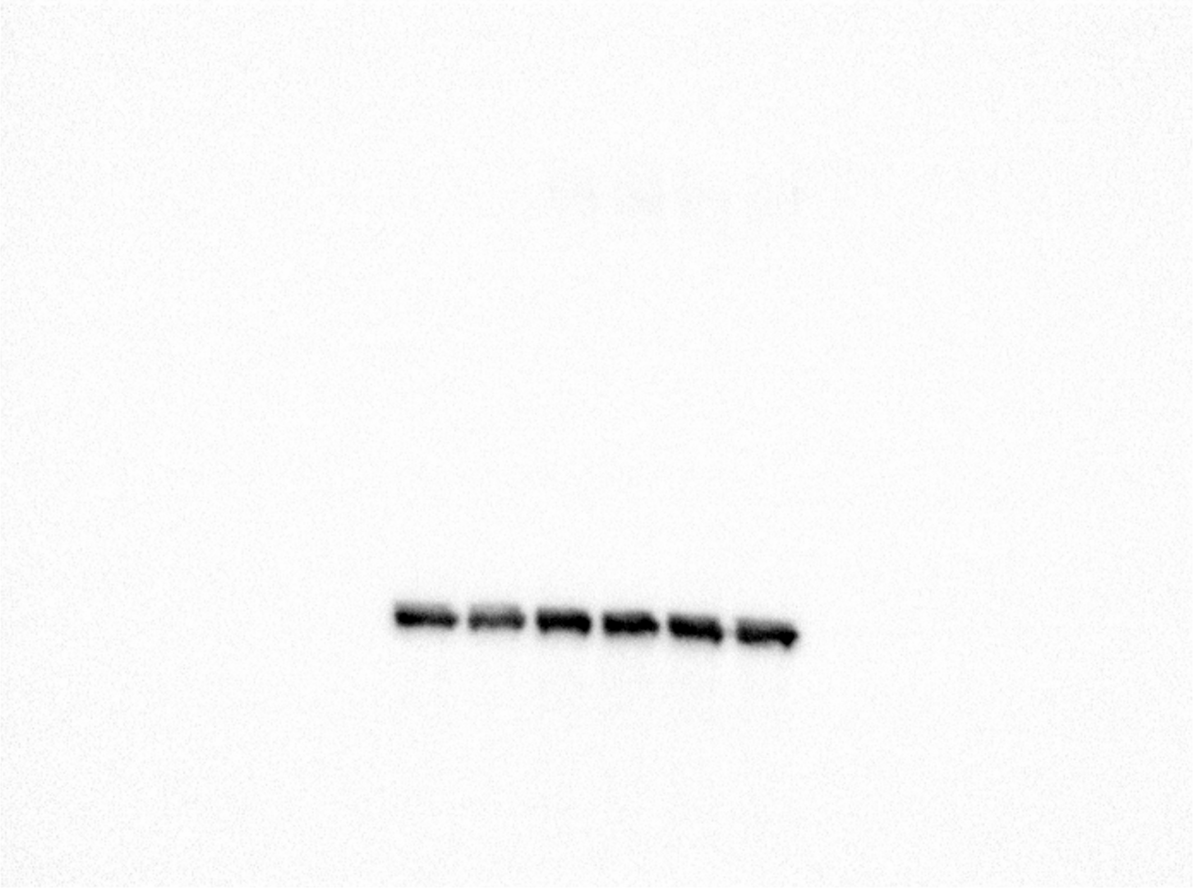

Figure S7a aFLAG

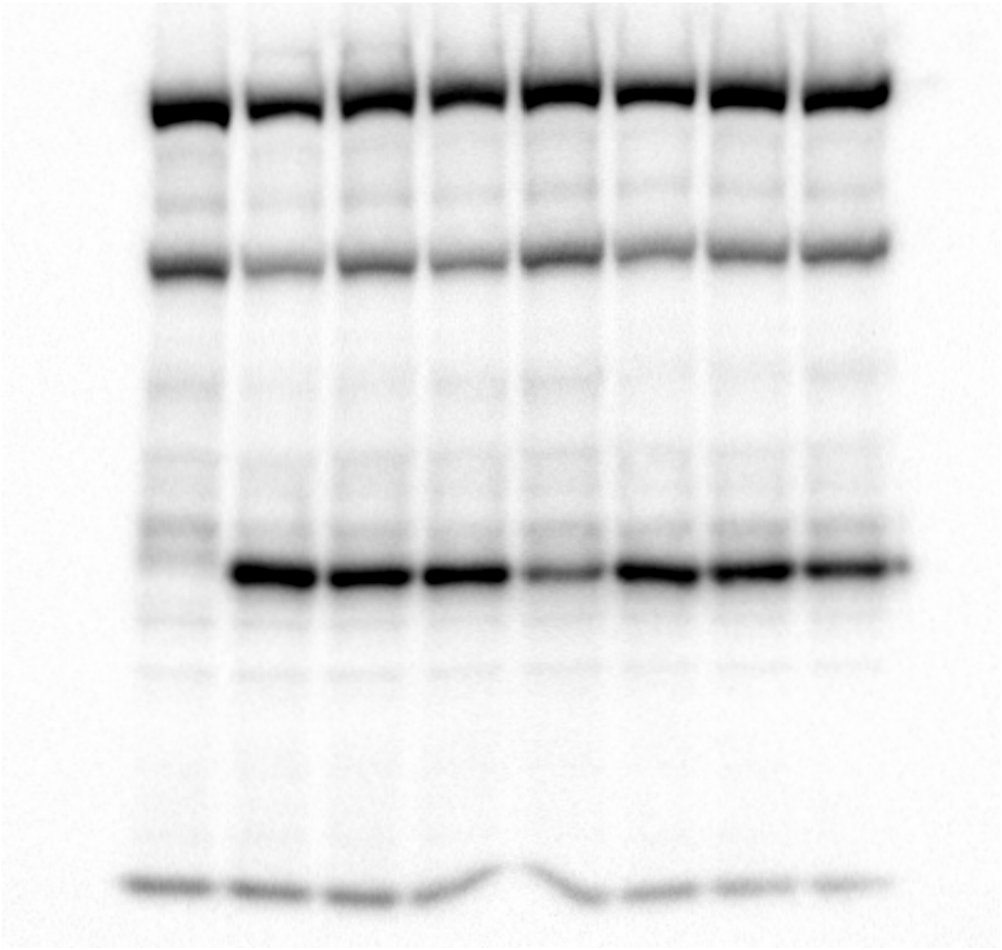

Figure S7a aHA

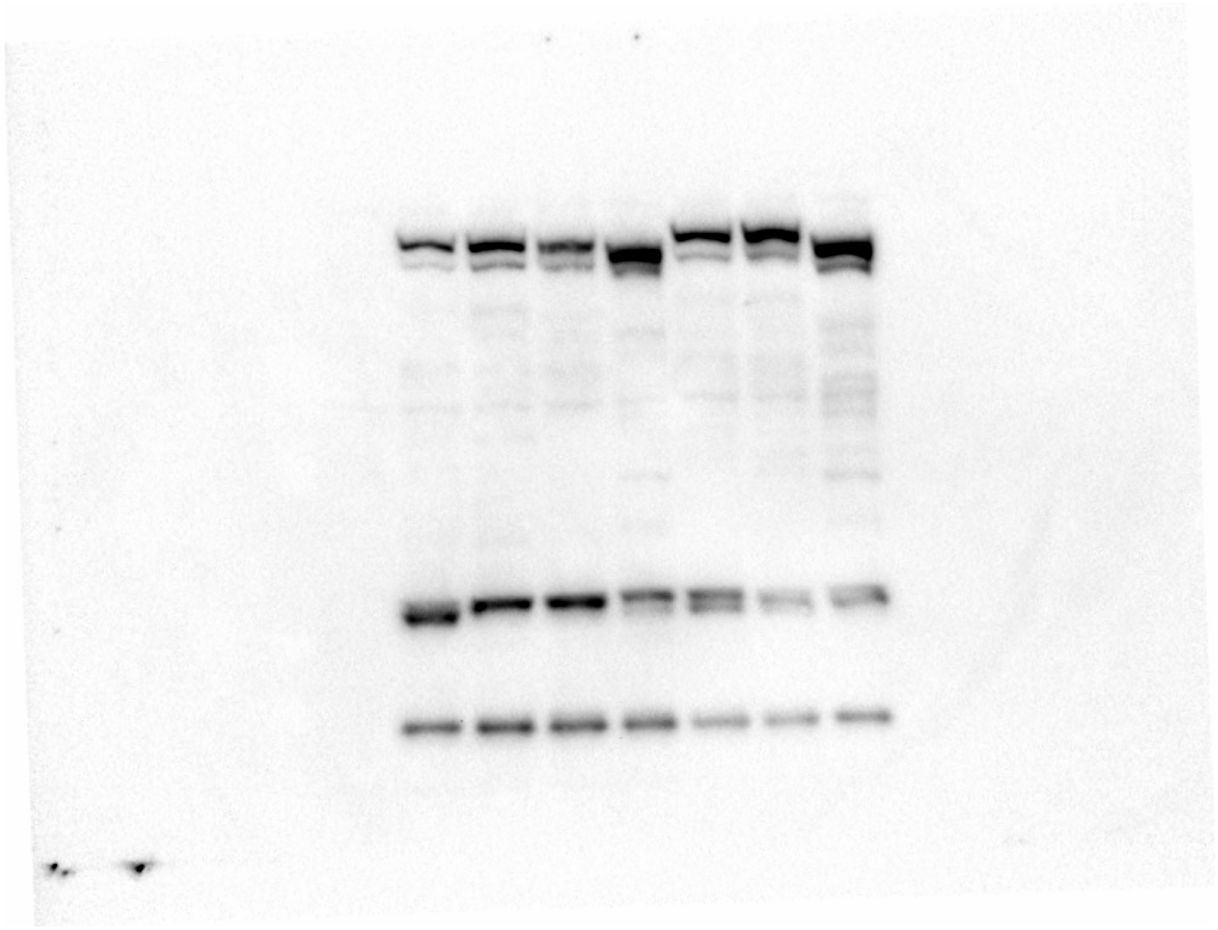

Figure S7a aGAPDH

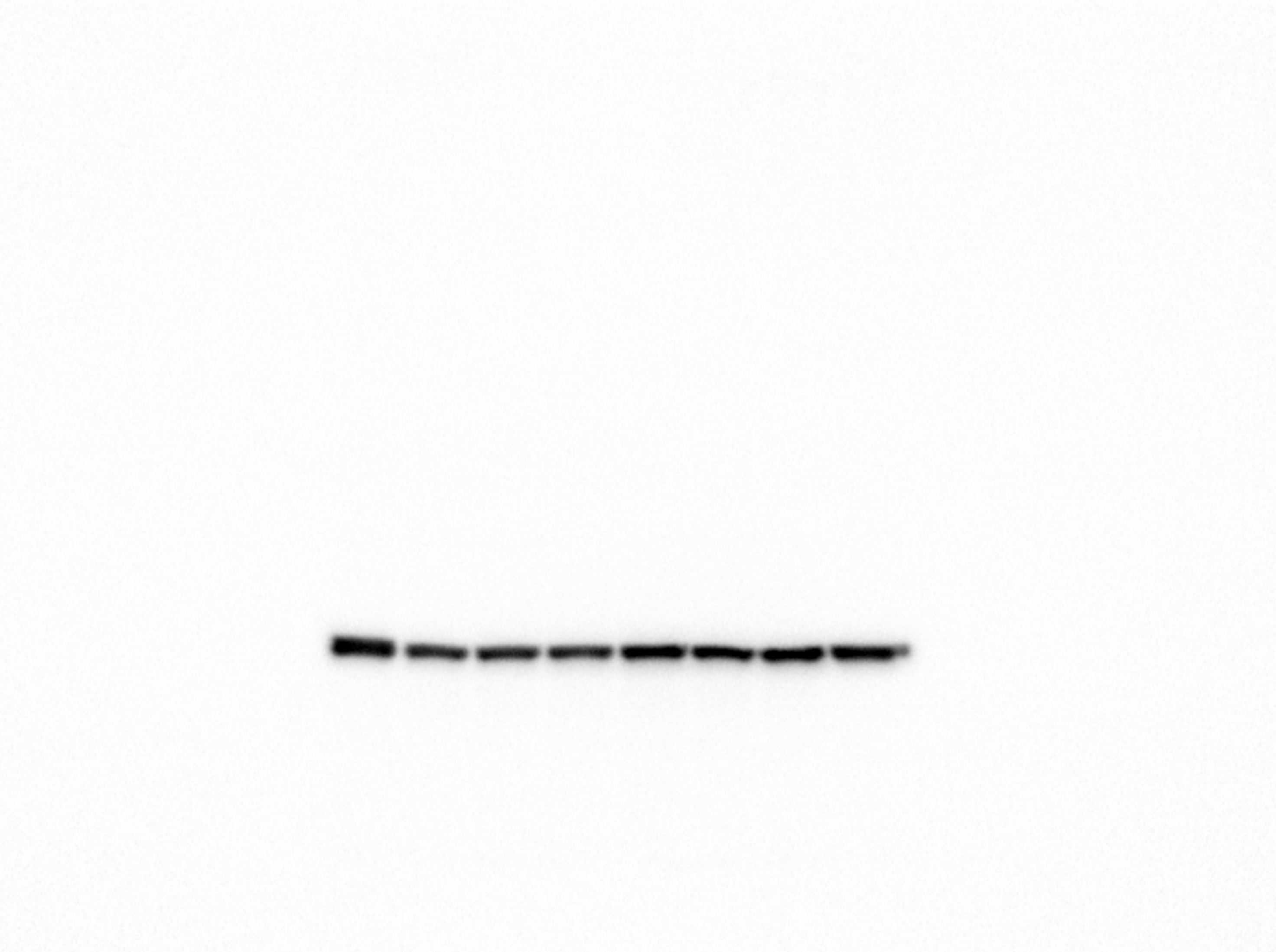

Figure S7b aV5

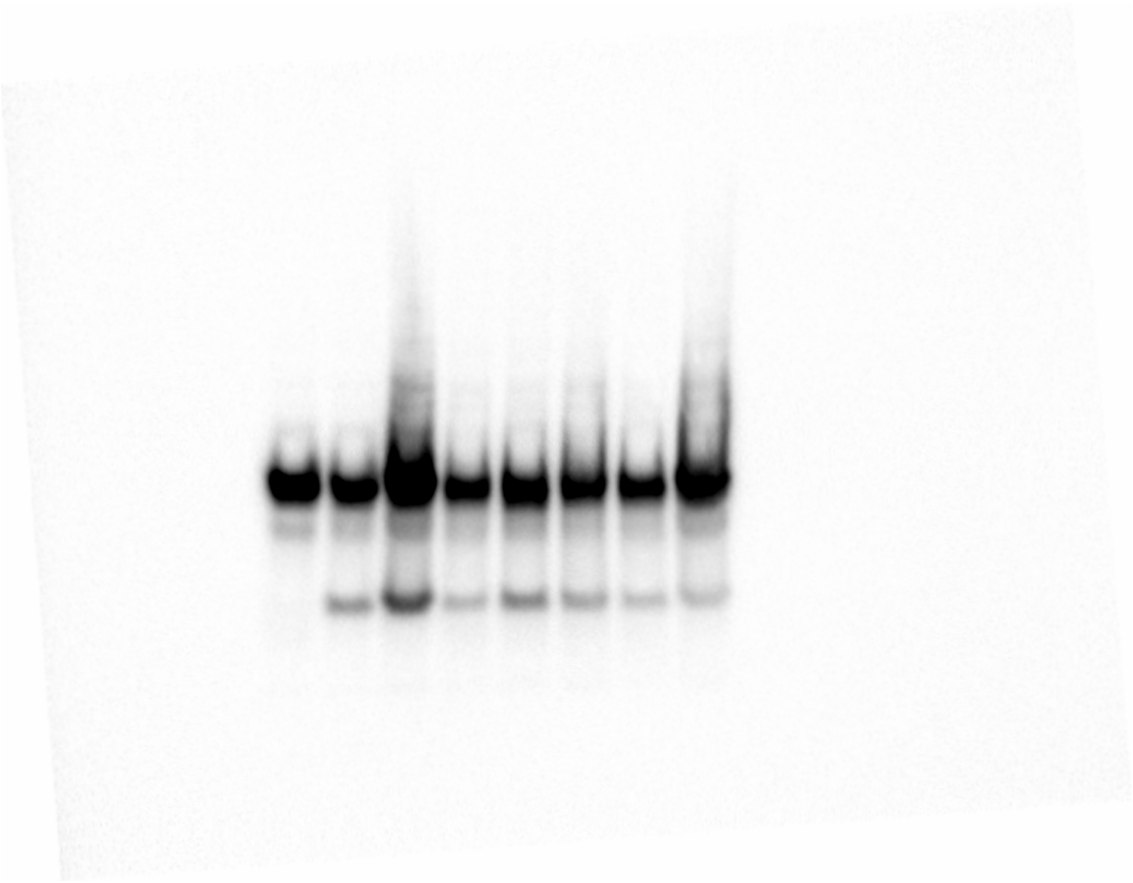

Figure S7b aMyc

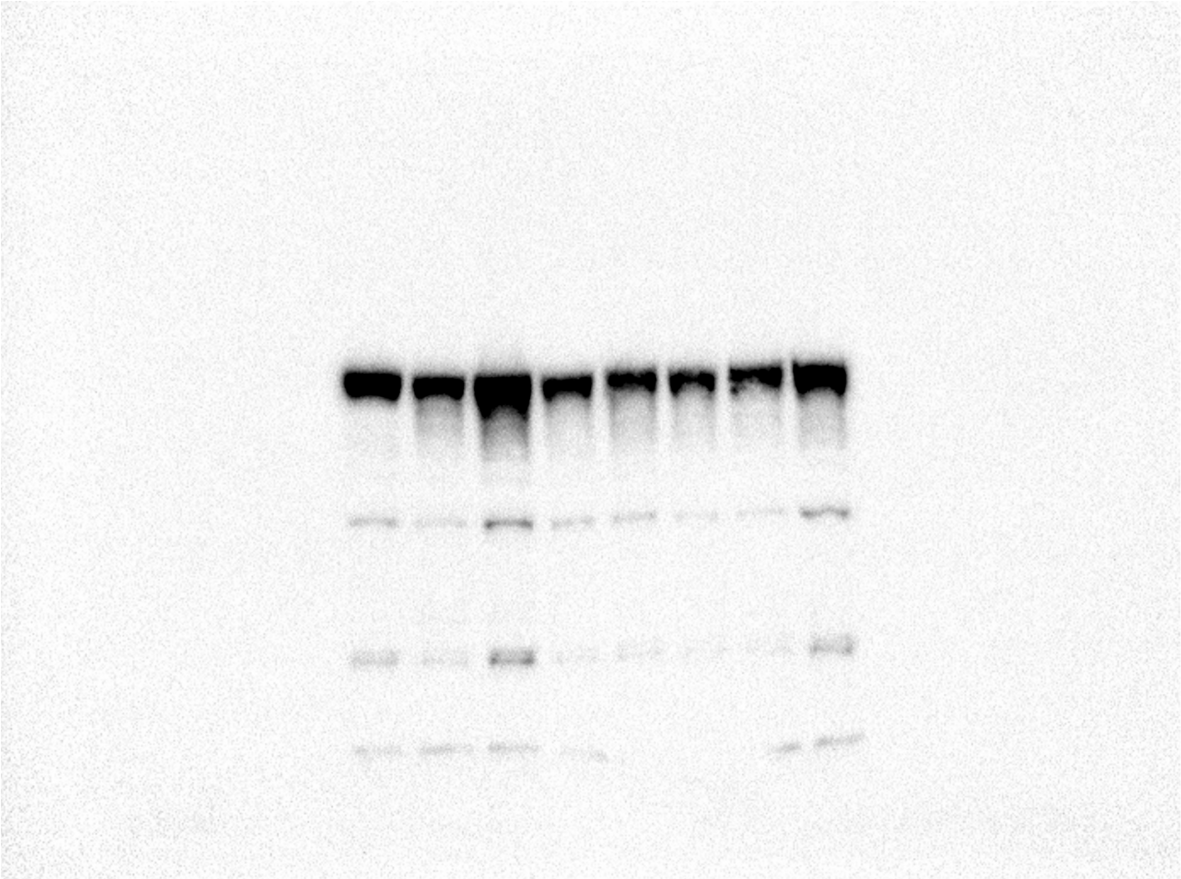

Figure S7b aHA

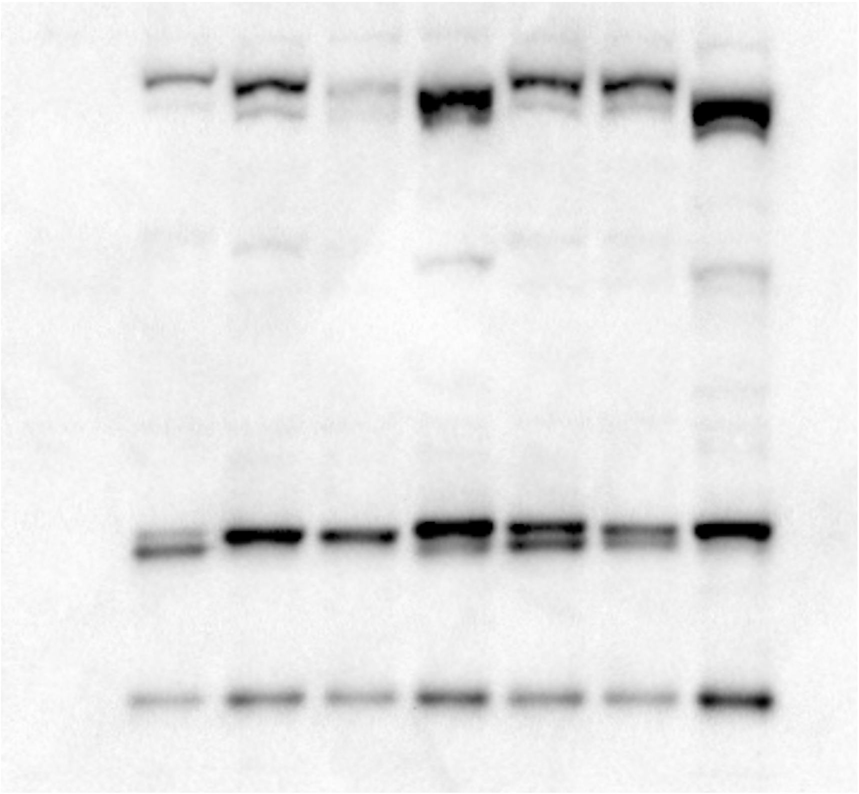

Figure S7b aGAPDH

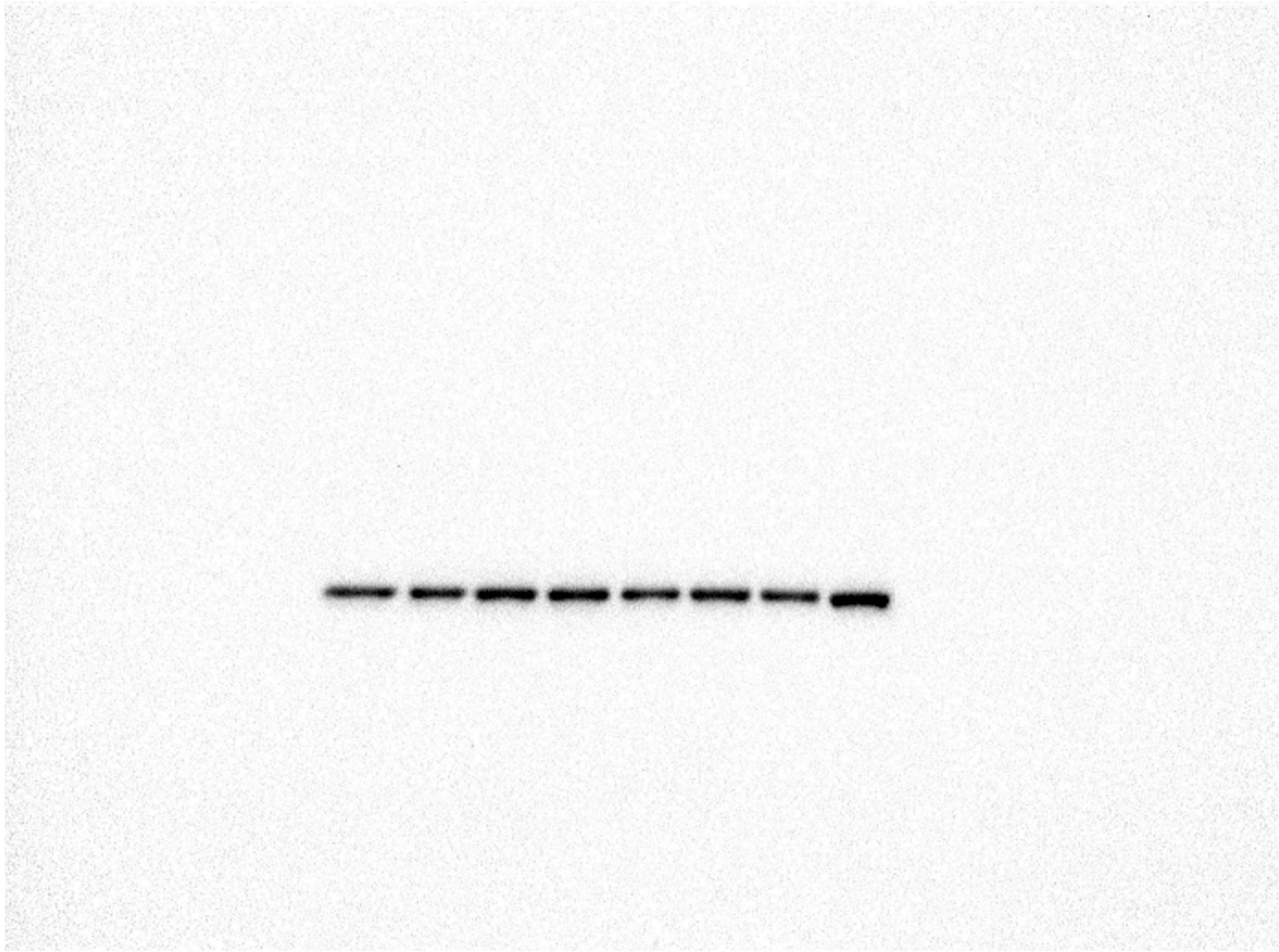

Figure S7c aFLAG

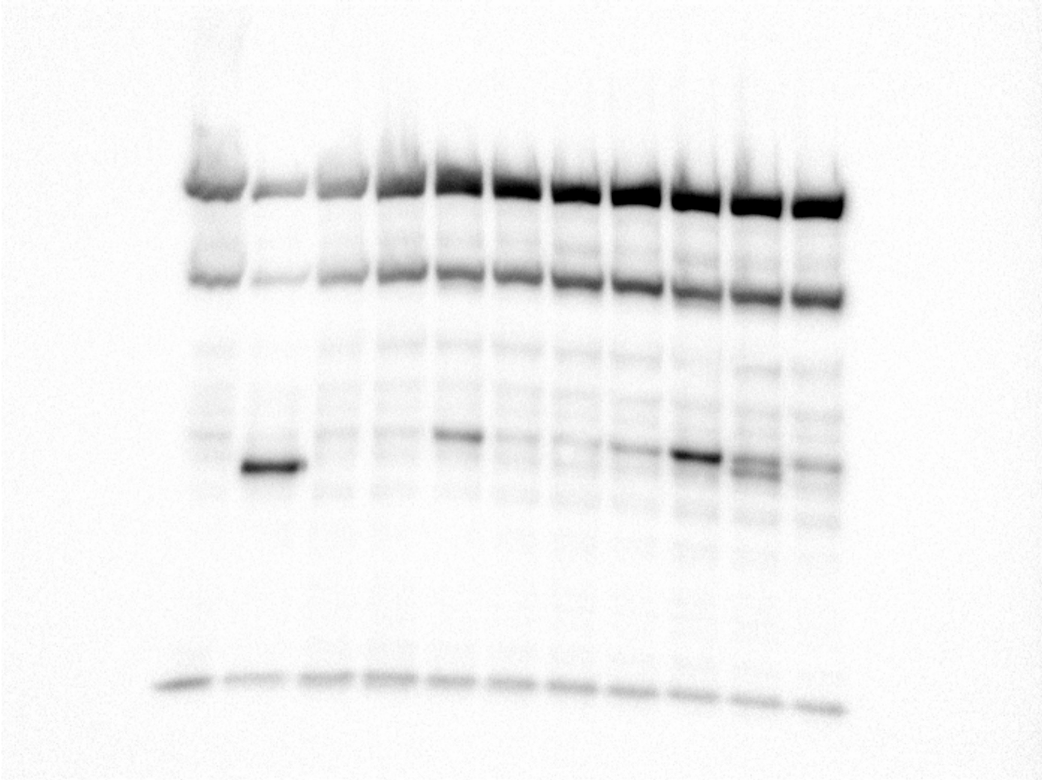

Figure S7c aHA

low

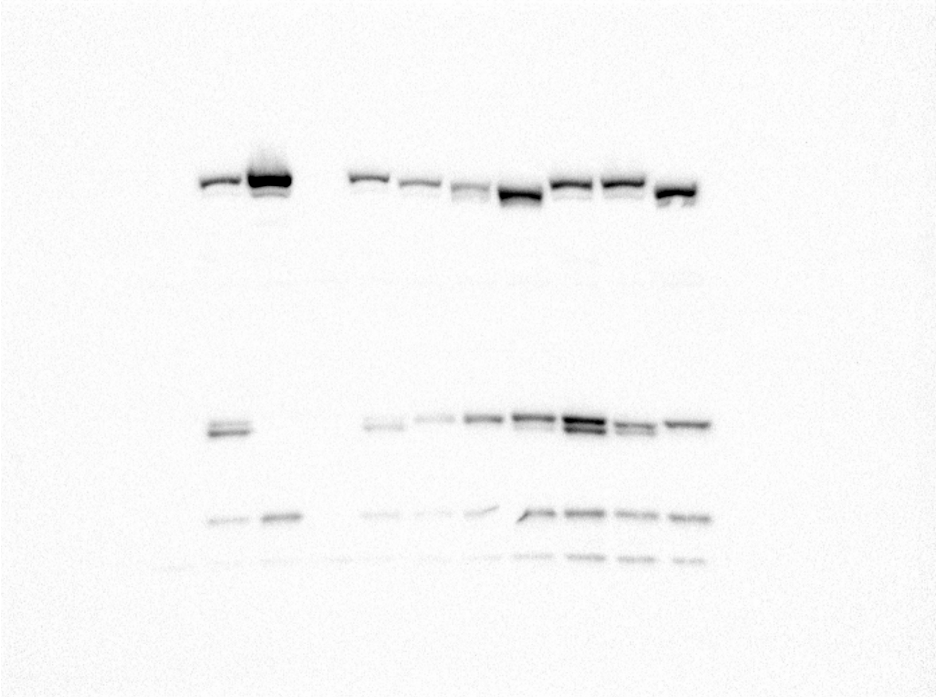

high

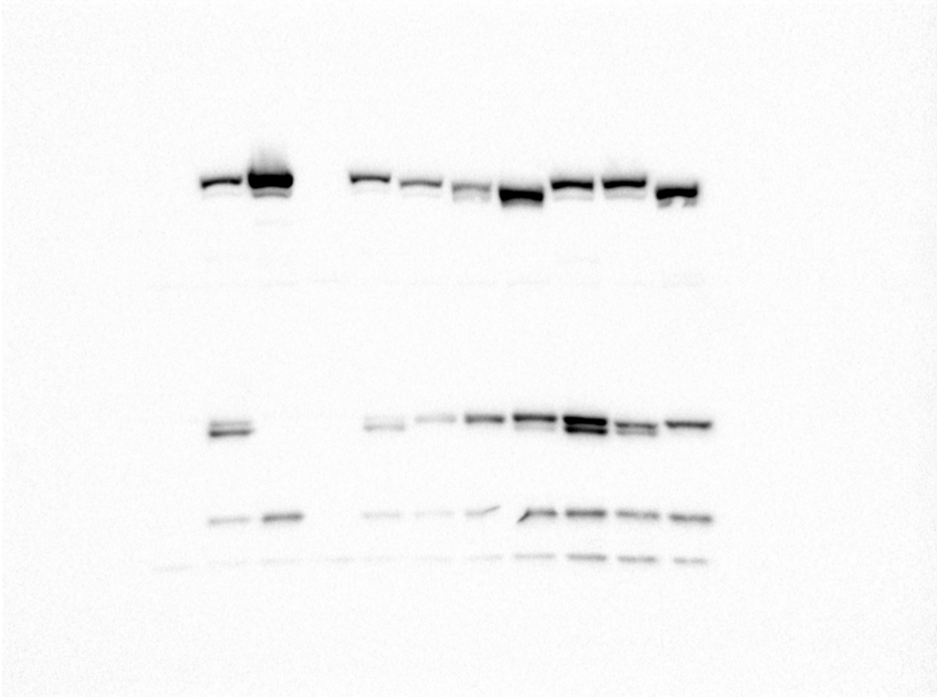

Figure S7c aGAPDH

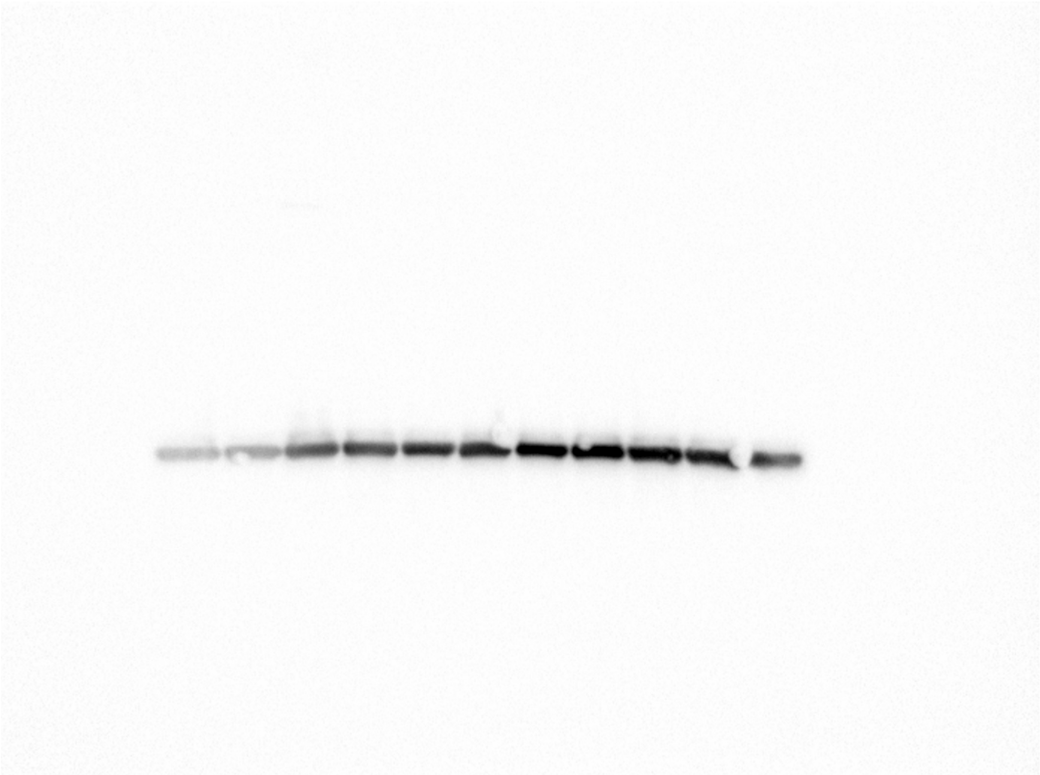

Figure S7d aFLAG

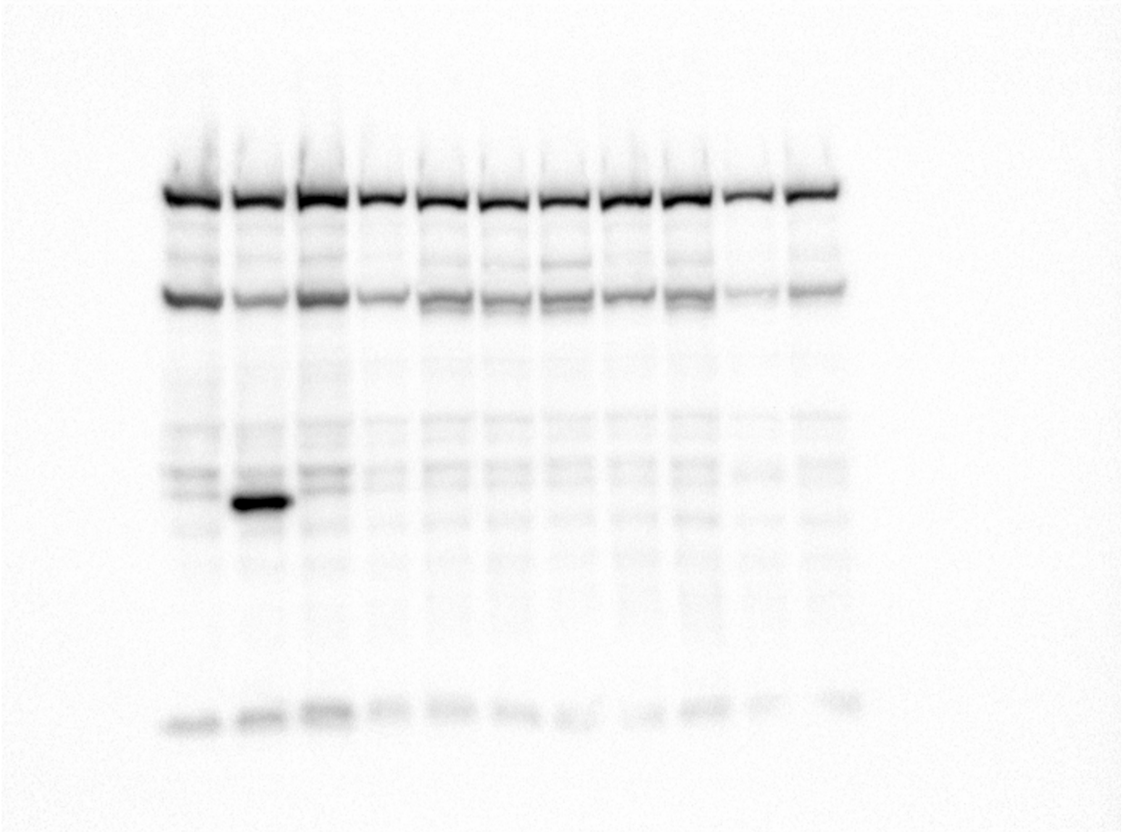

Figure S7d aHA

low

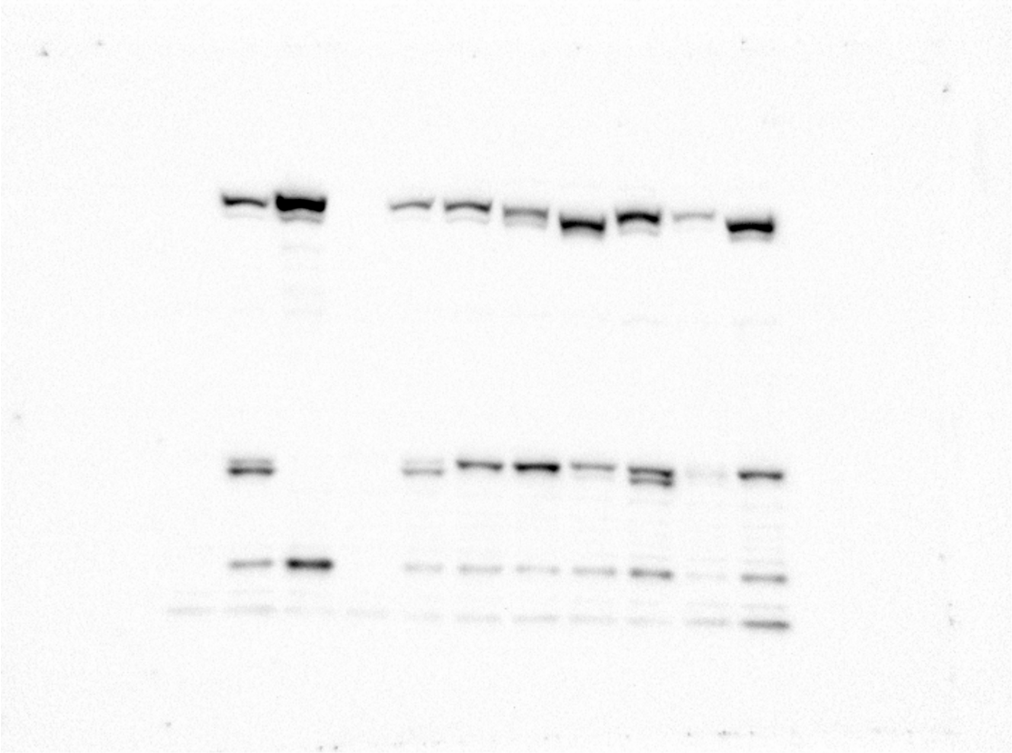

high

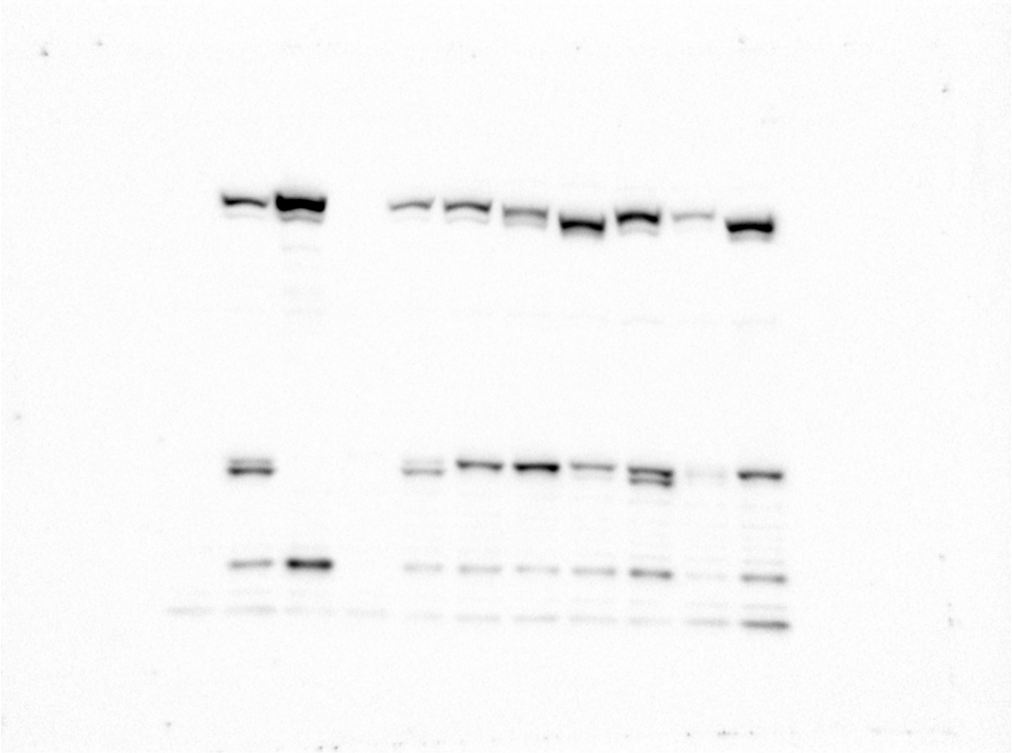

Figure S7d aGAPDH

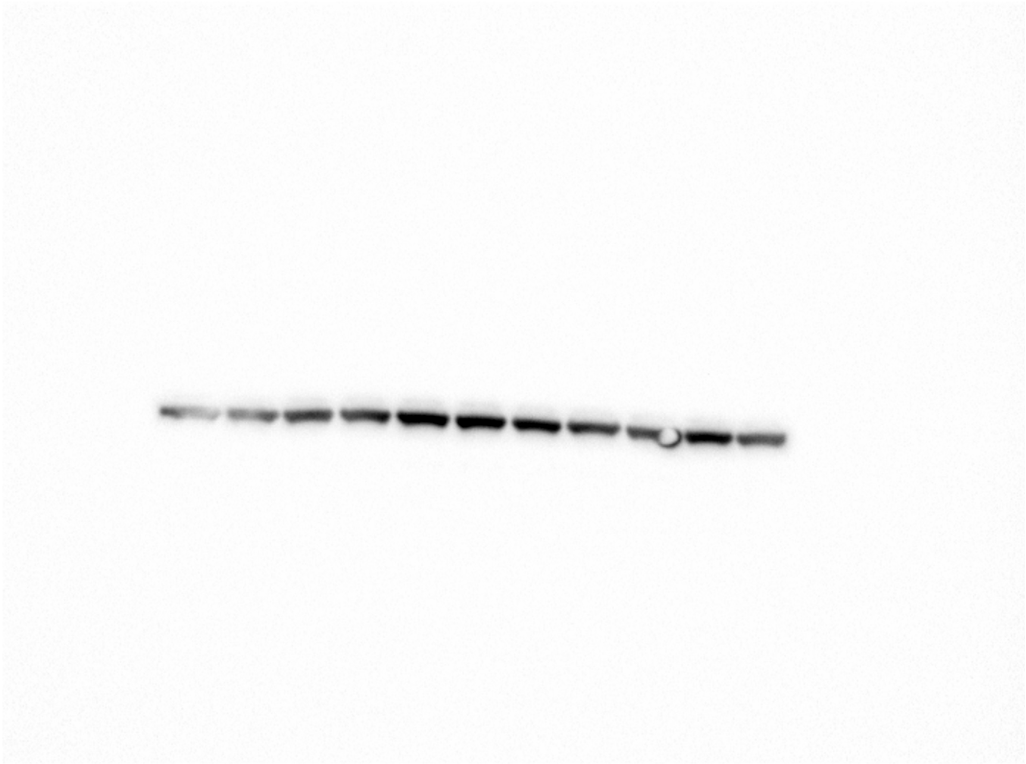

Figure S8

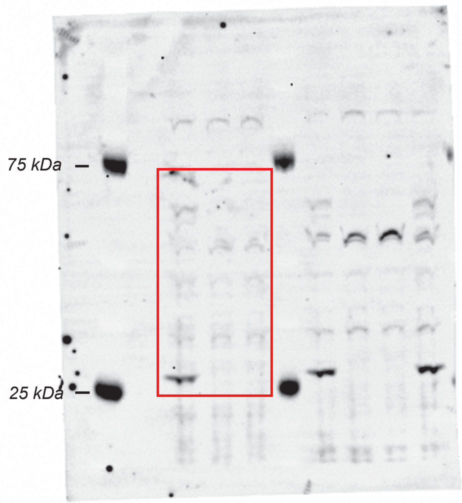

α-CARD8 CTD

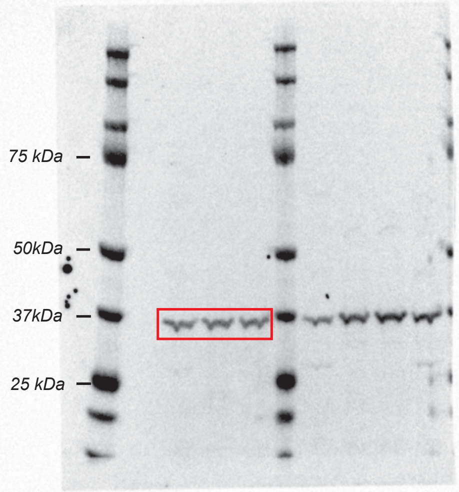

α-TUBULIN

Figure S14a aV5 (top)

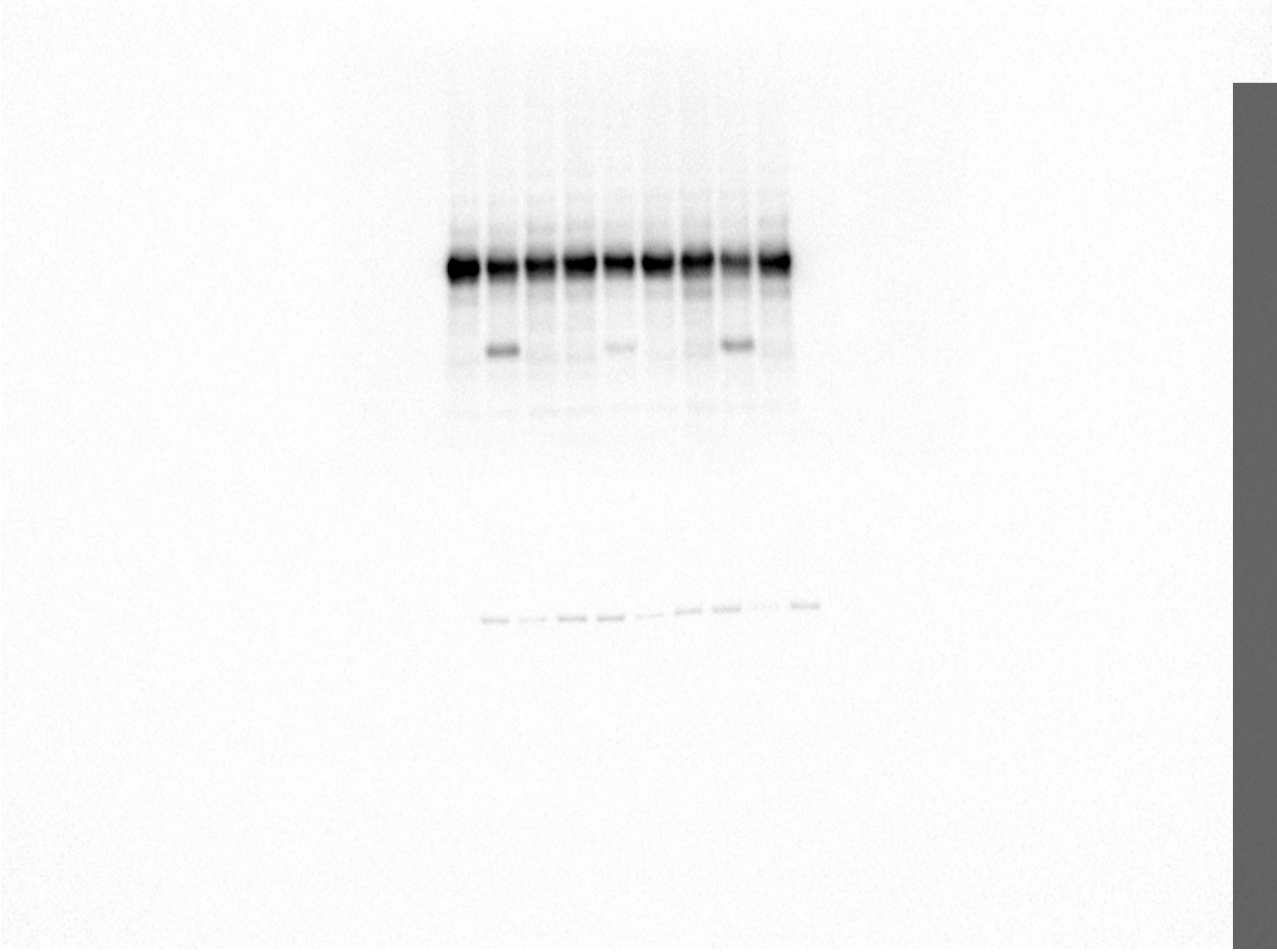

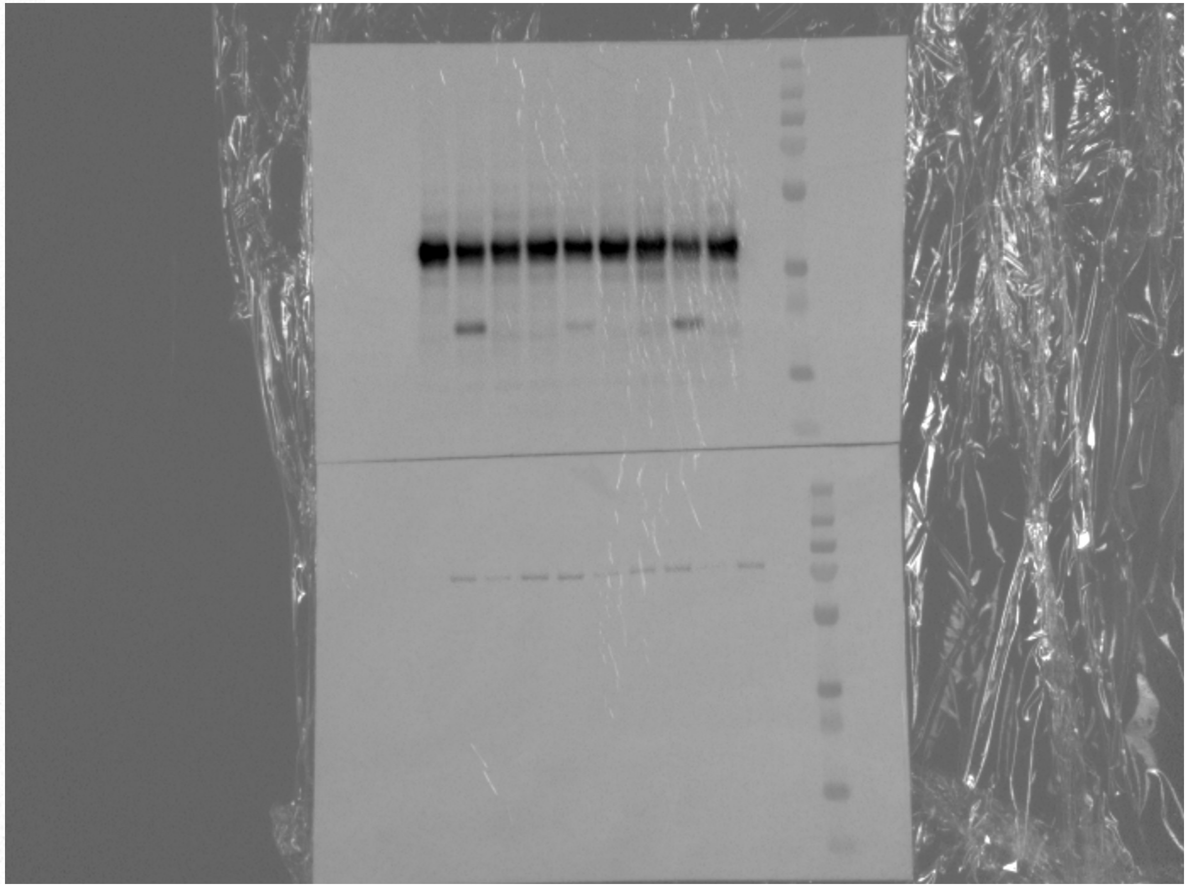

Figure S14a aMyc (bottom half)

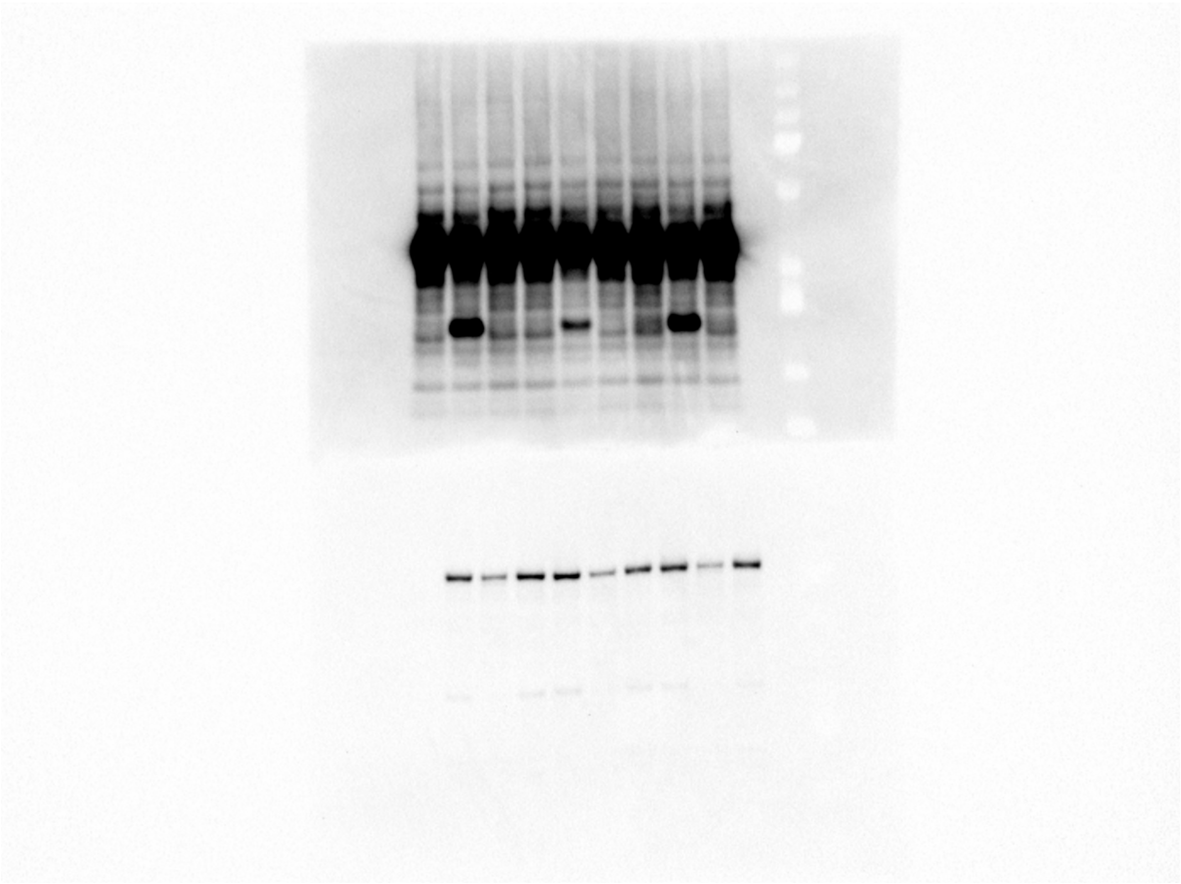

Figure S14a aHA

low

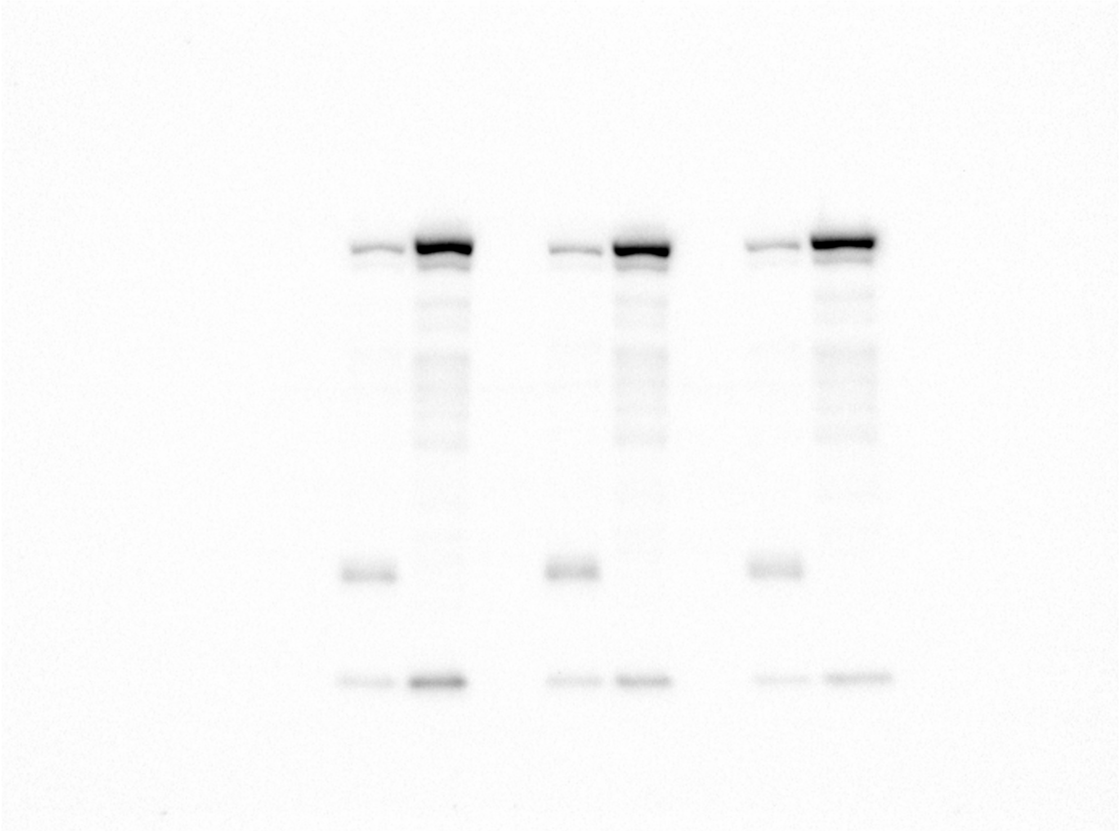

high

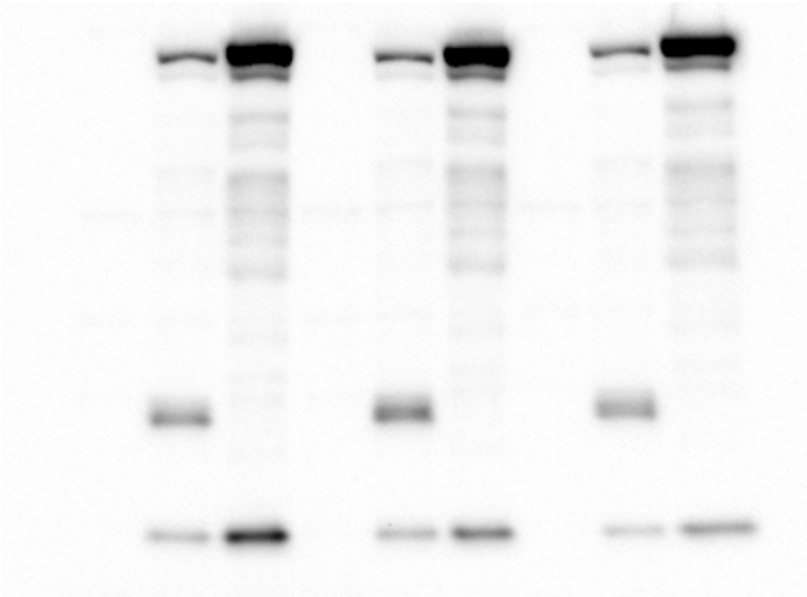

Figure S14a aGAPDH

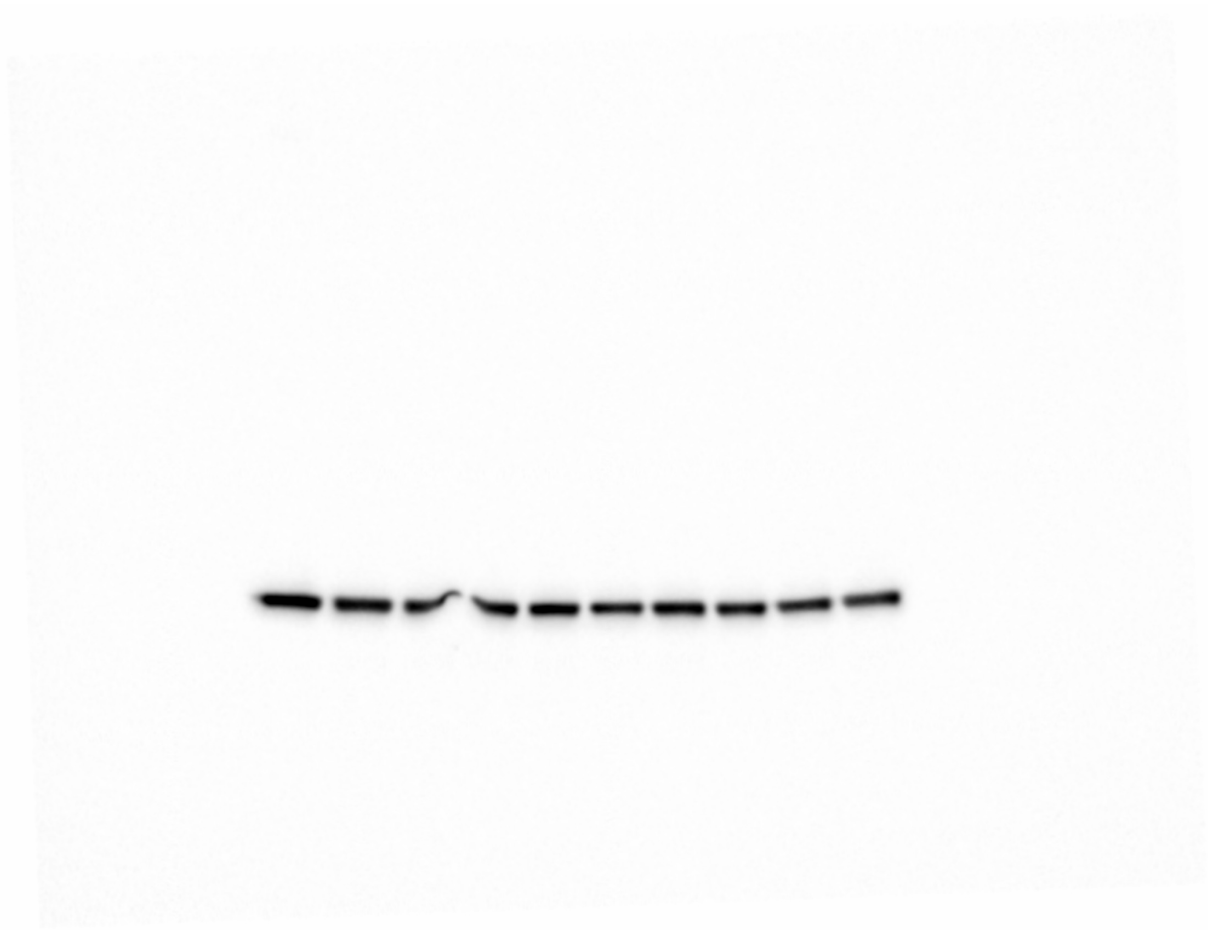

Figure S14b aFLAG

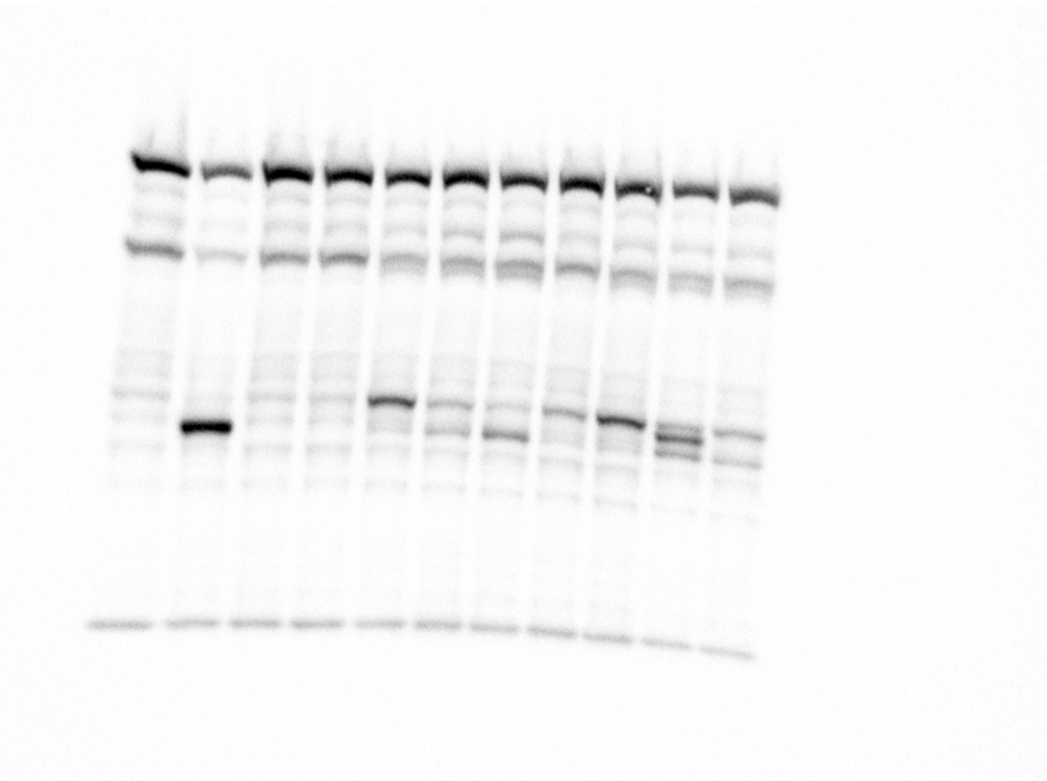

Figure S14b aHA

low

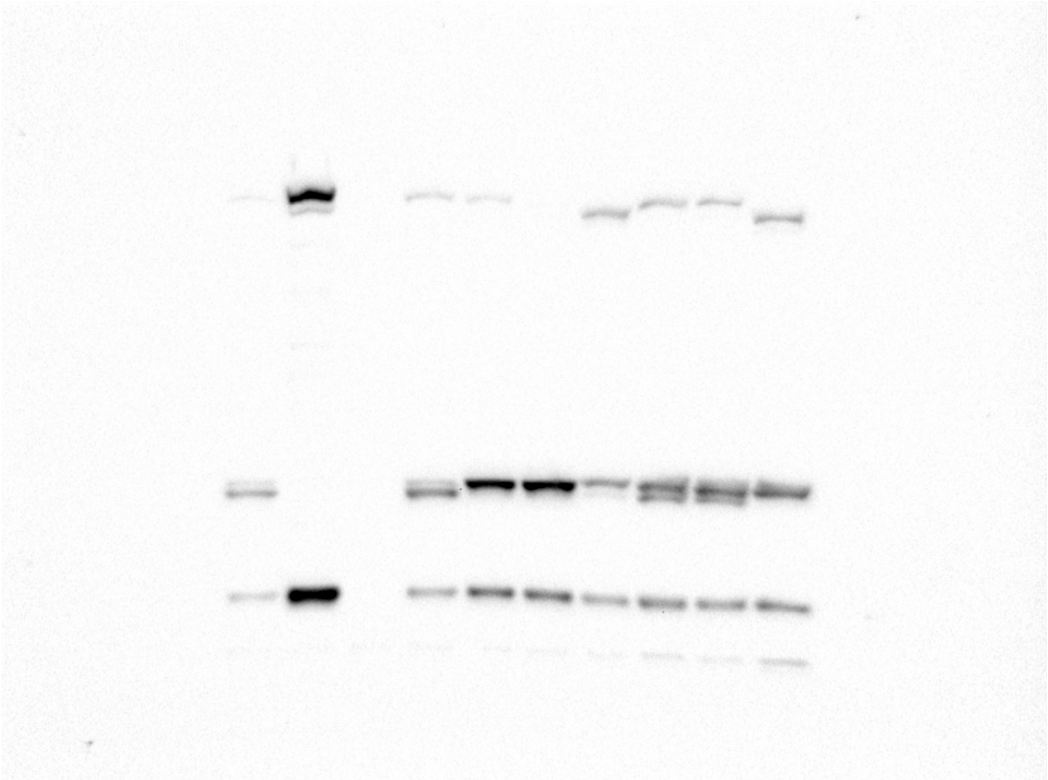

high

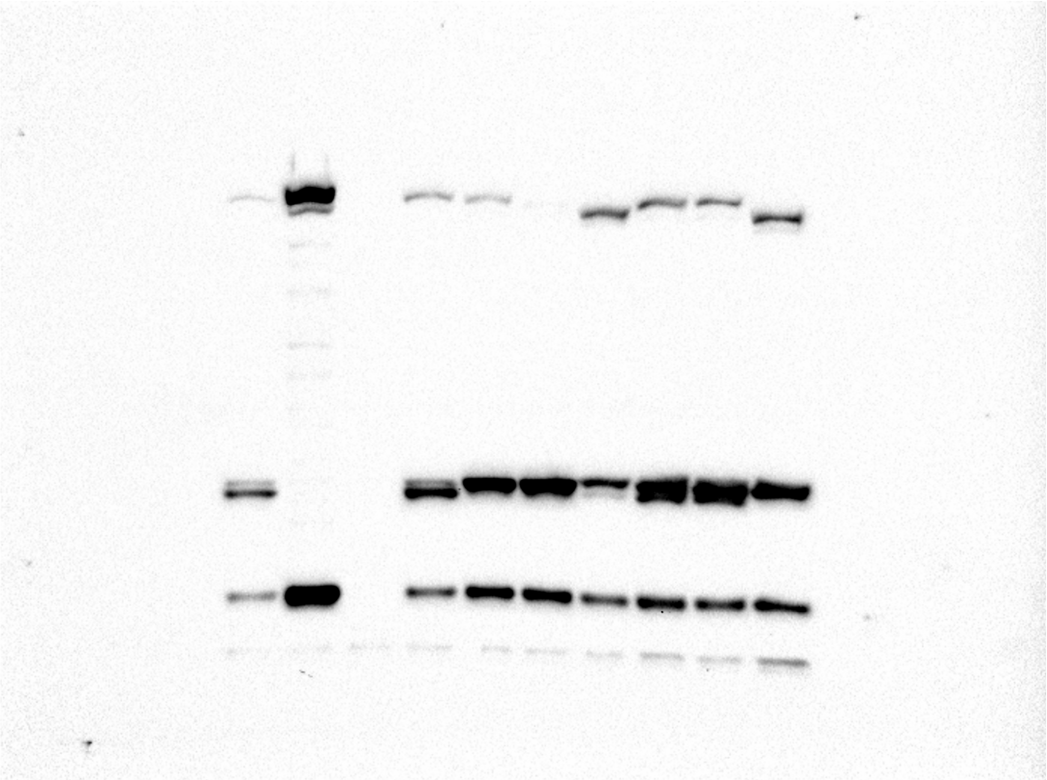

Figure S14b aGAPDH

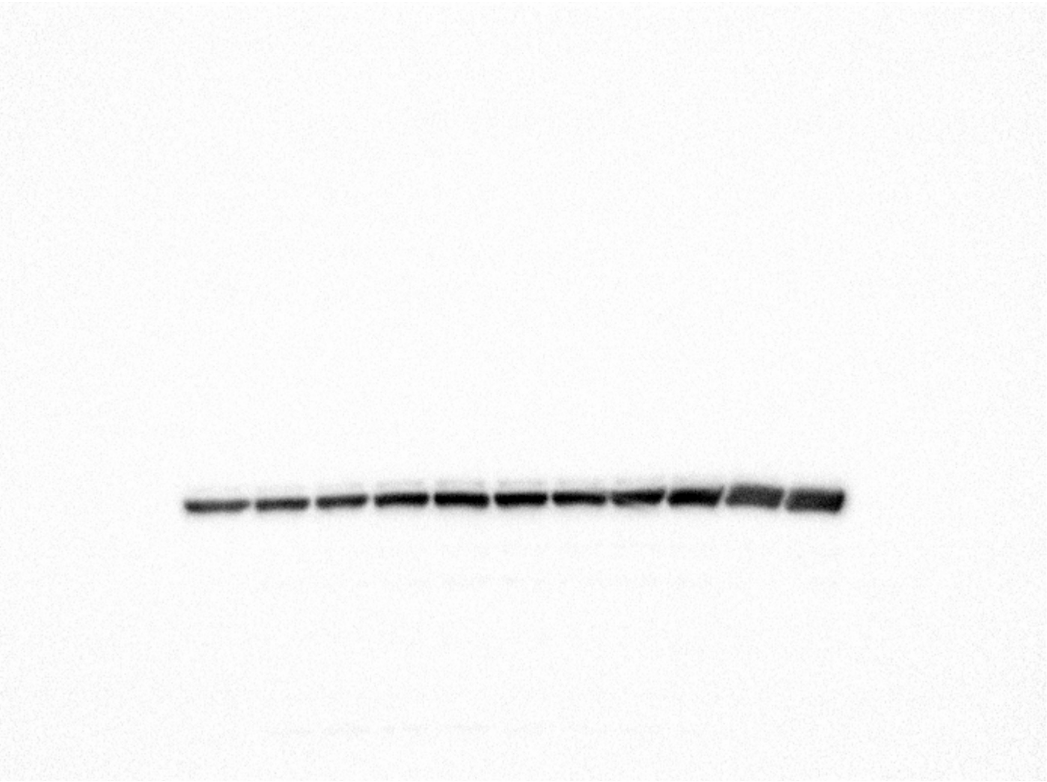

Figure S16a (left) aFLAG

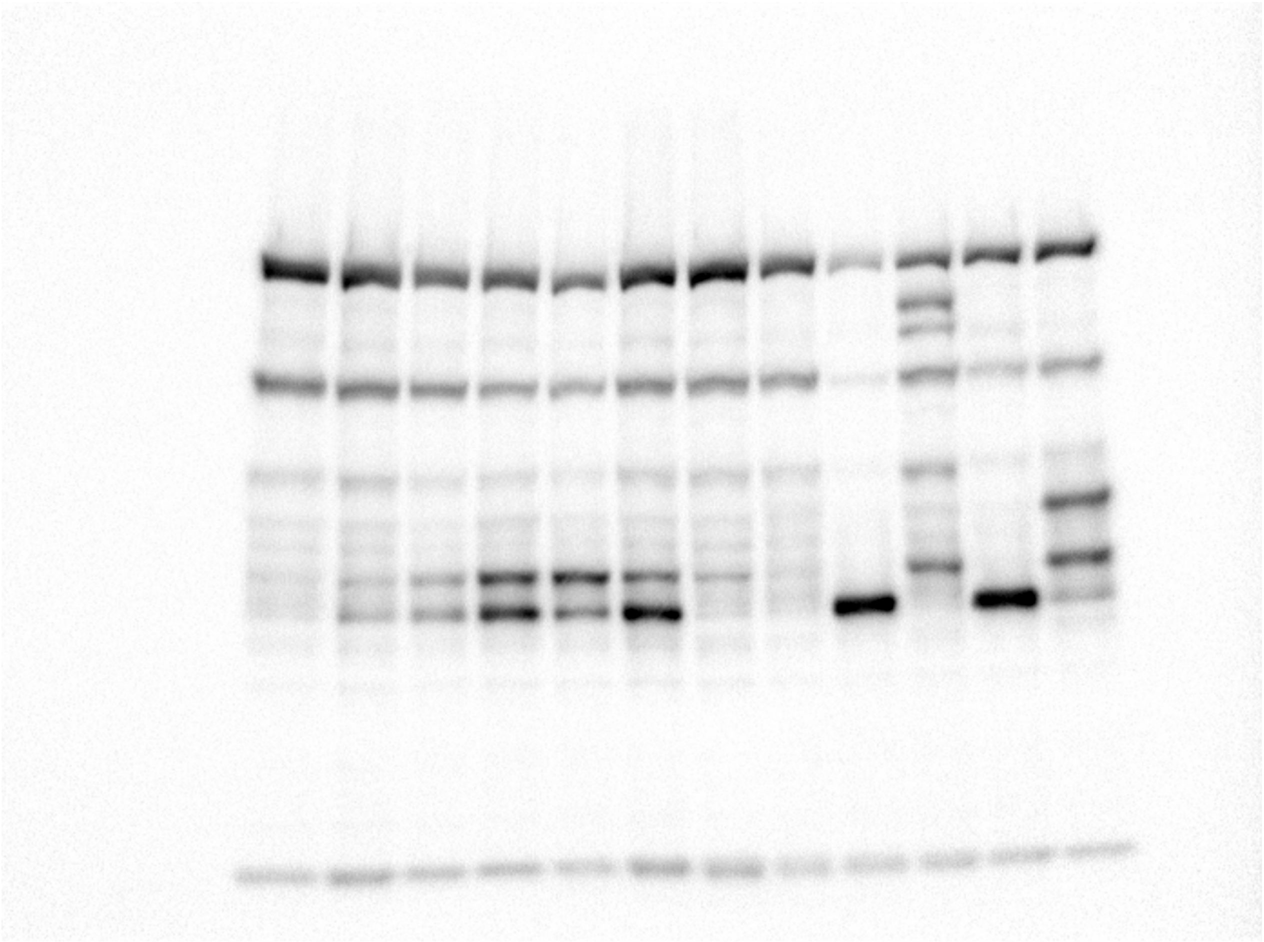

Figure S16a (left) aHA

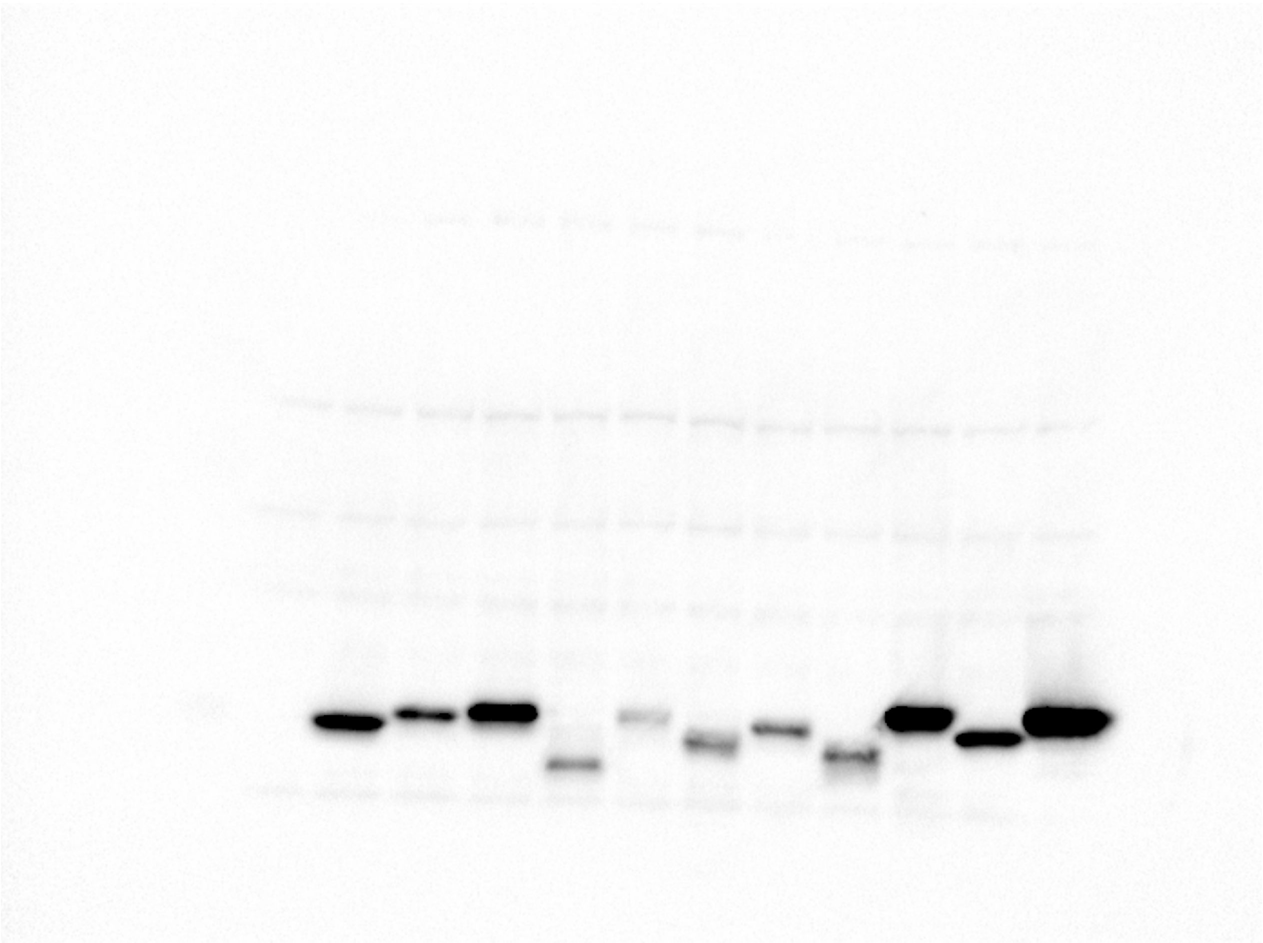

Figure S16a (left) aGAPDH

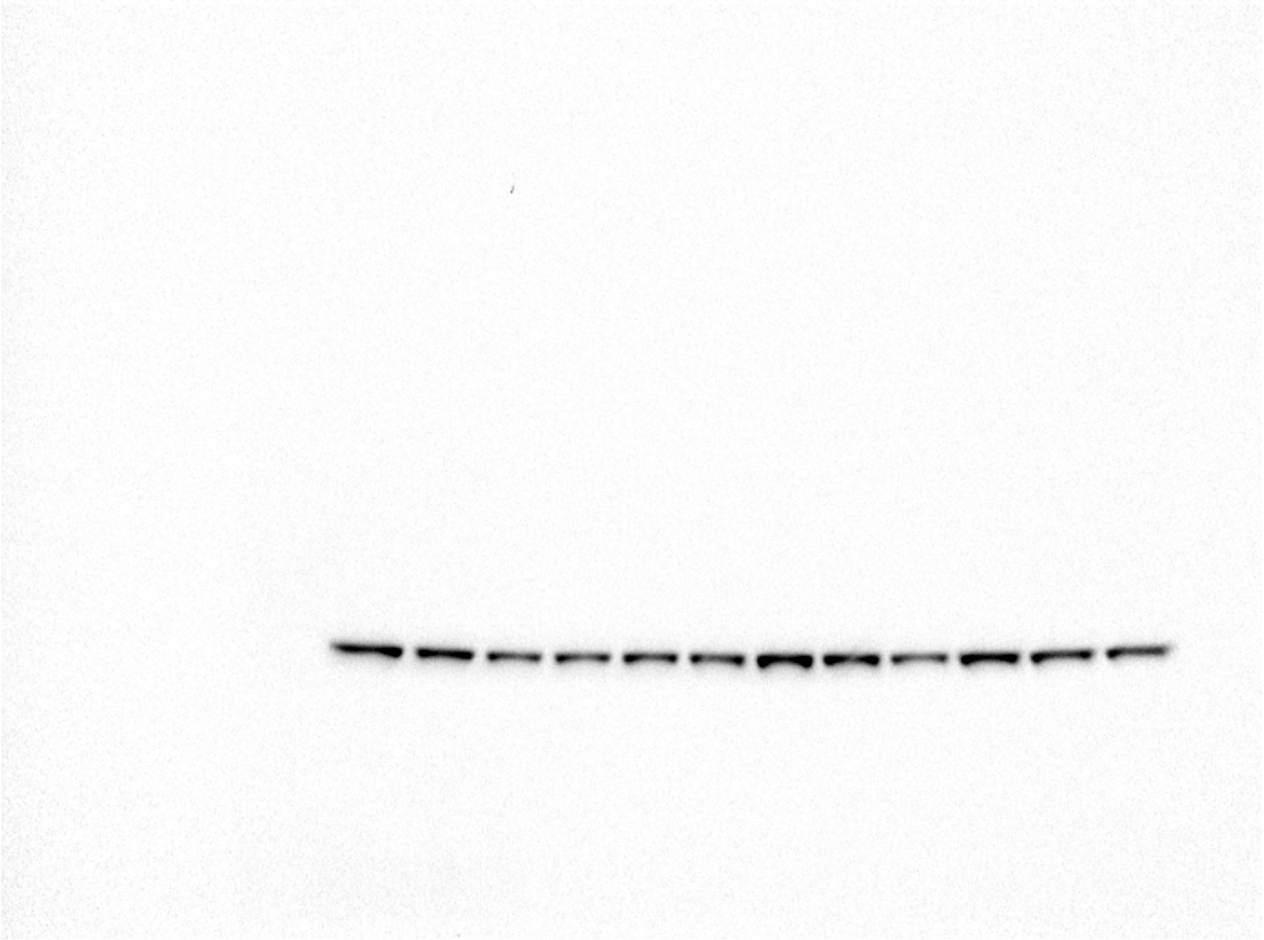

Figure S16a (right) aFLAG

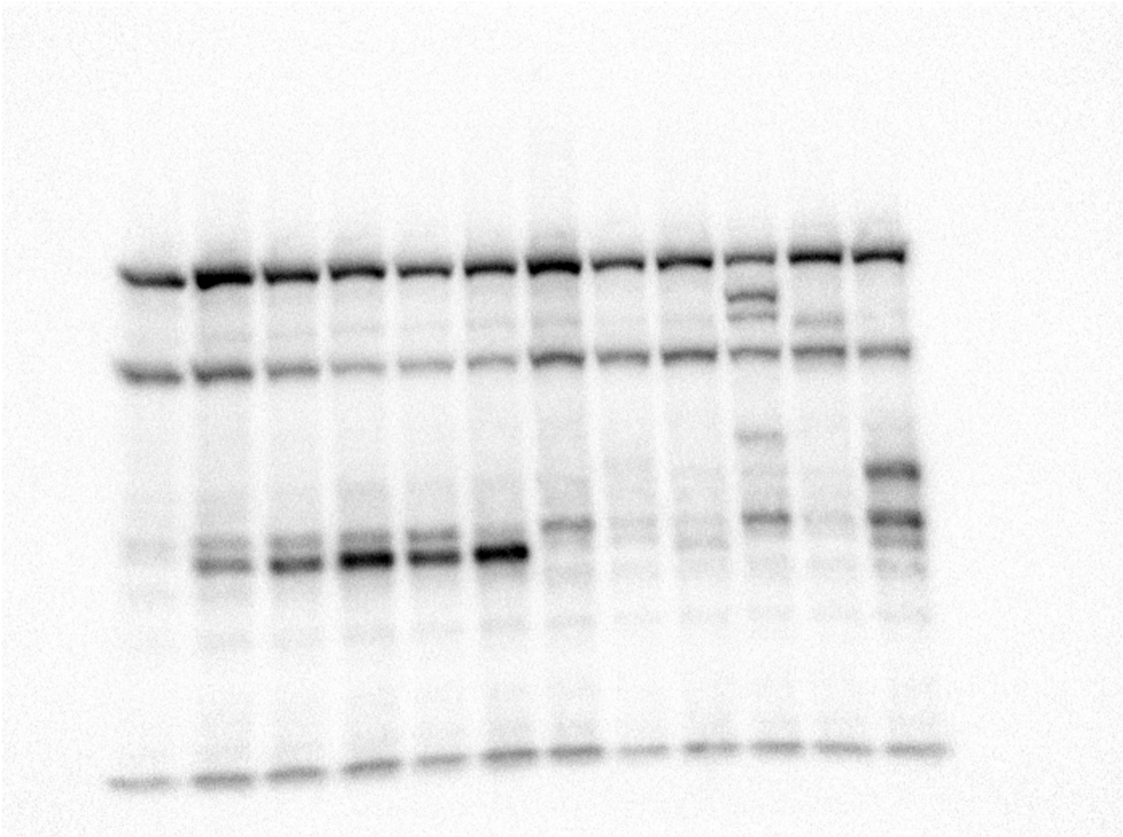

Figure S16a (right) aHA

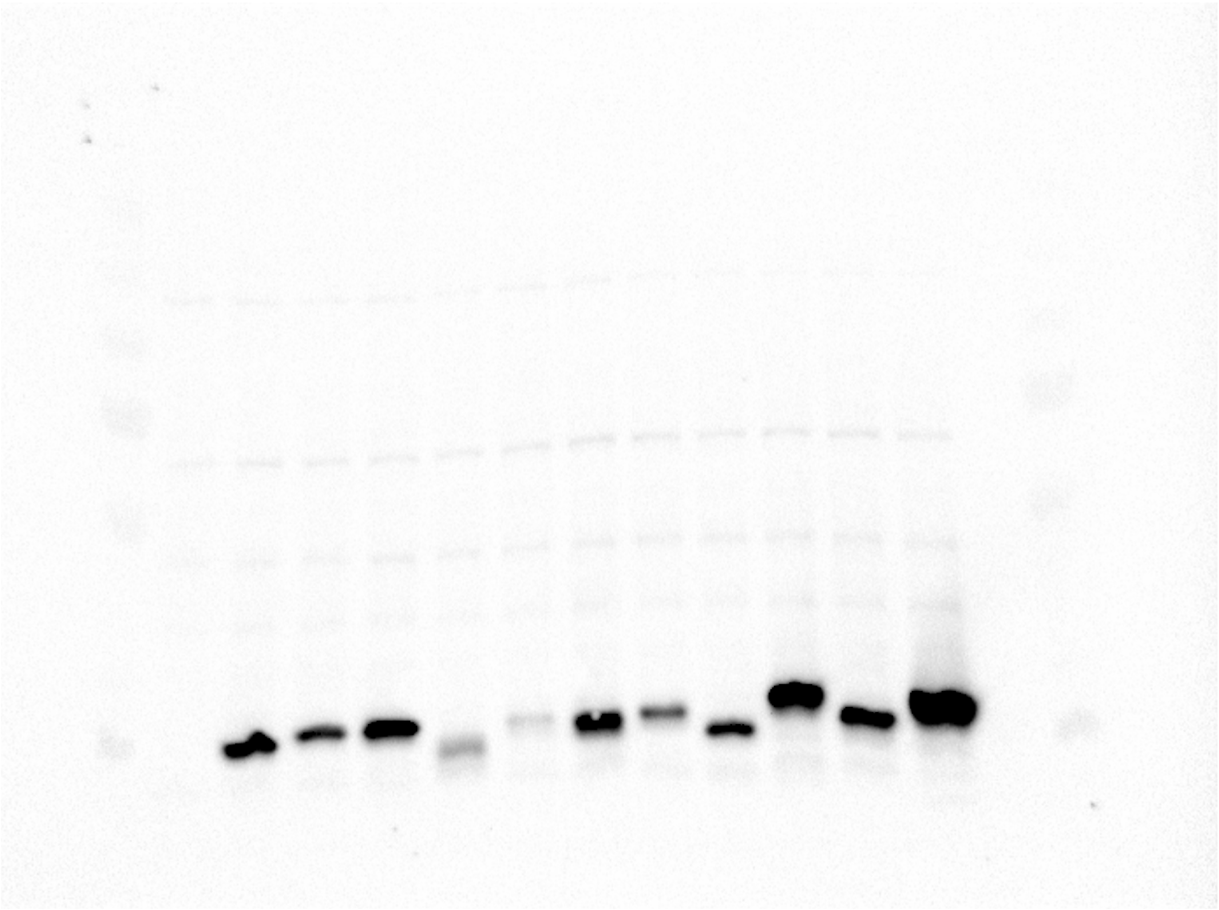

Figure S16a (right) aGAPDH

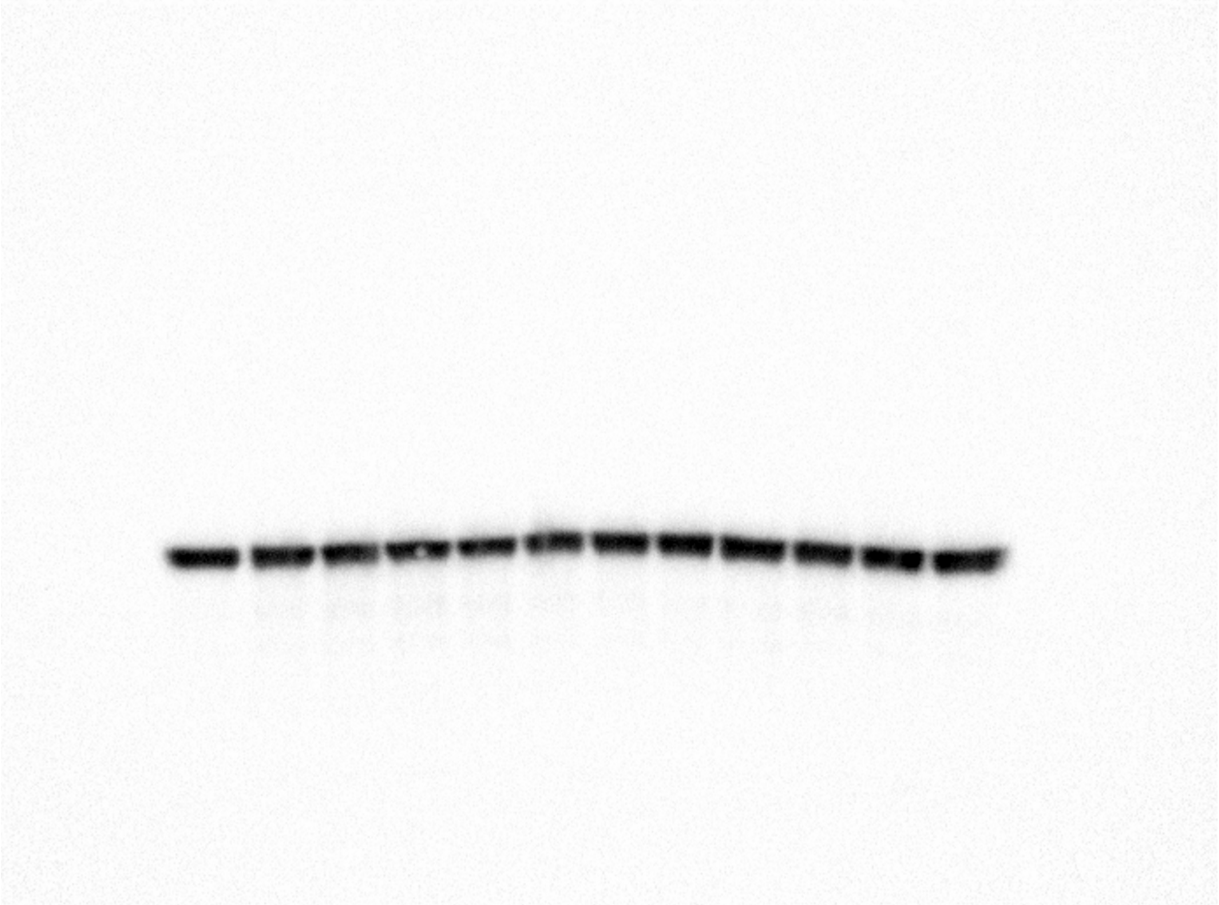

Figure S16b (left) aV5

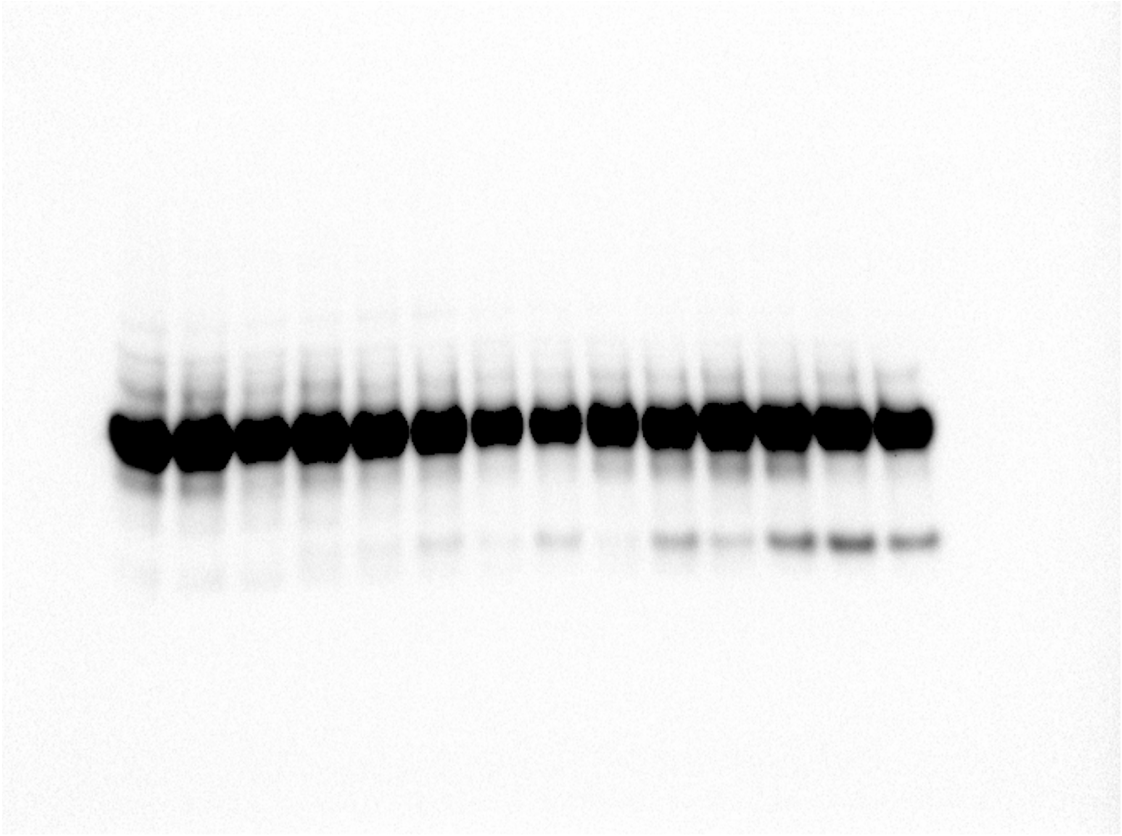

Figure S16b (left) aMyc

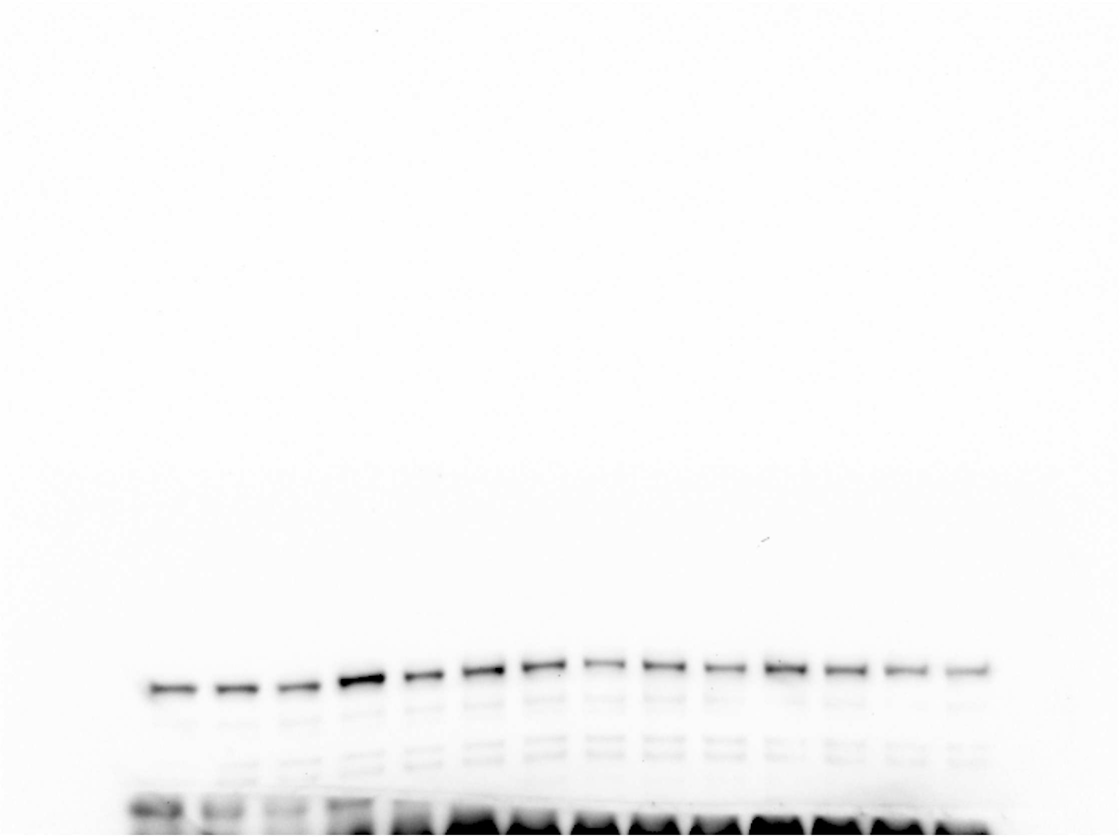

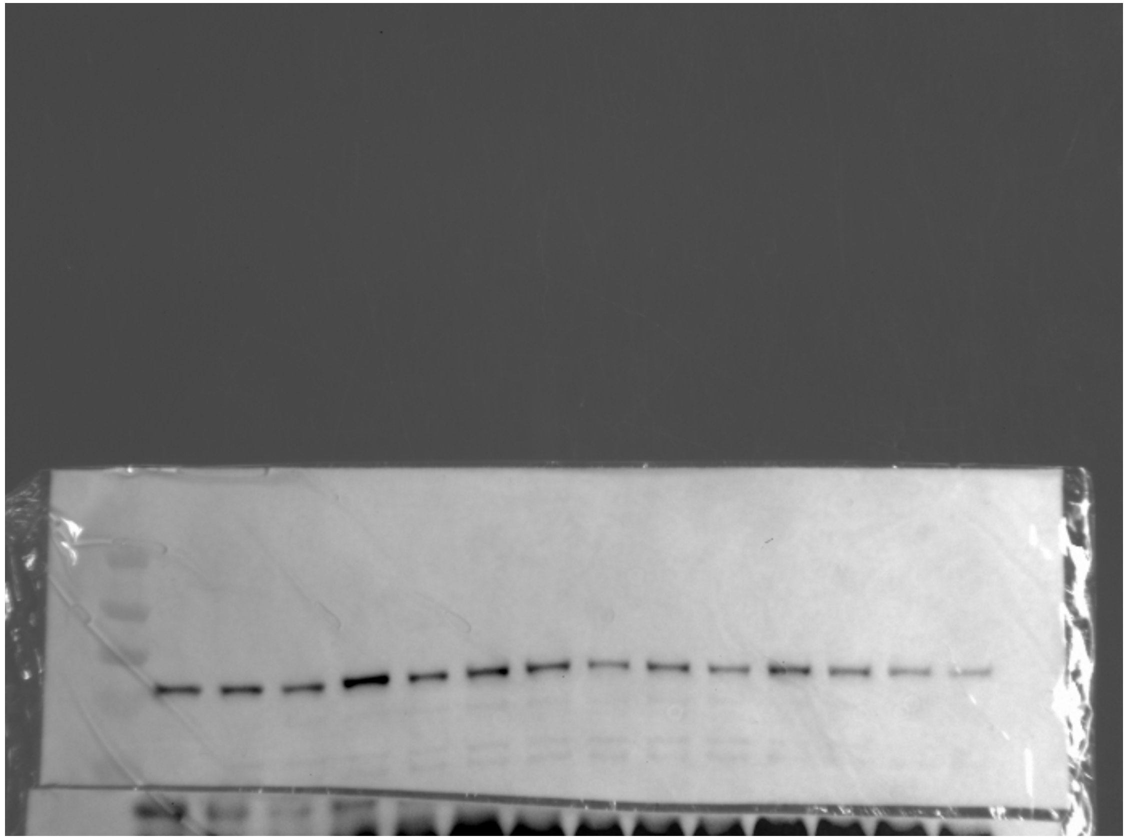

Figure S16b (left) aHA

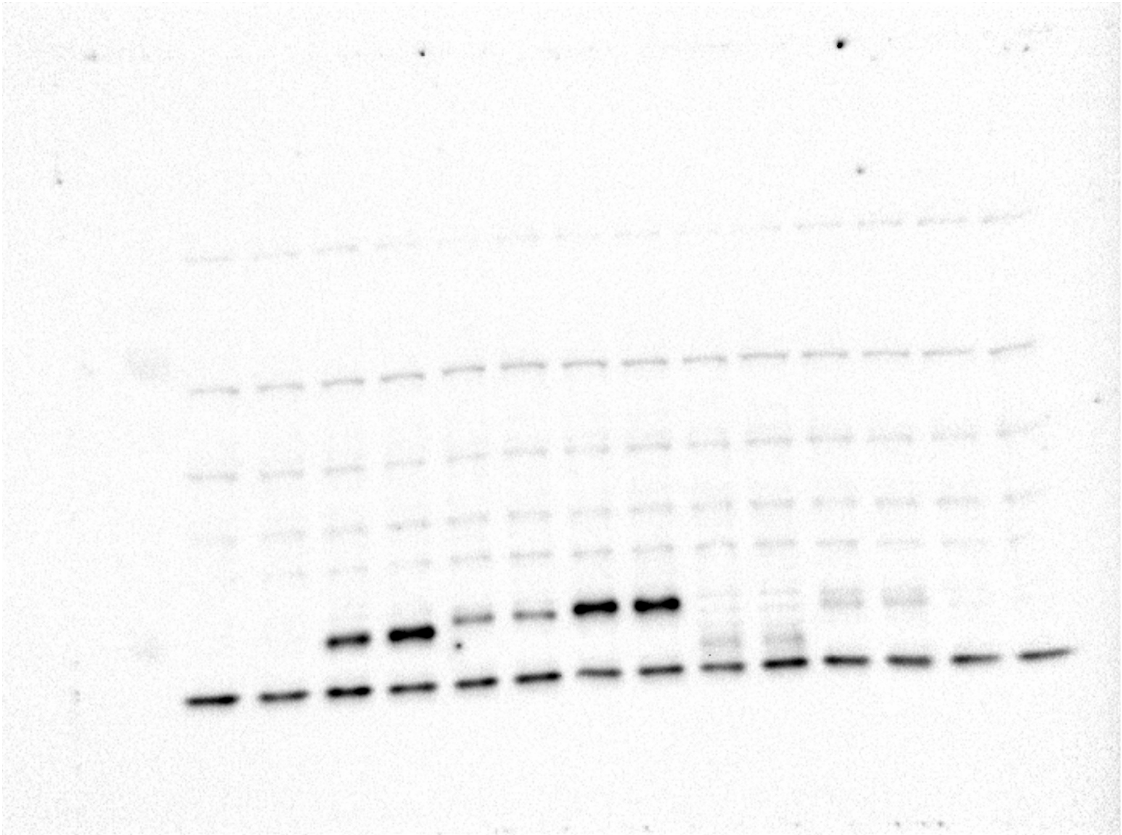

Figure S16b (left) aGAPDH

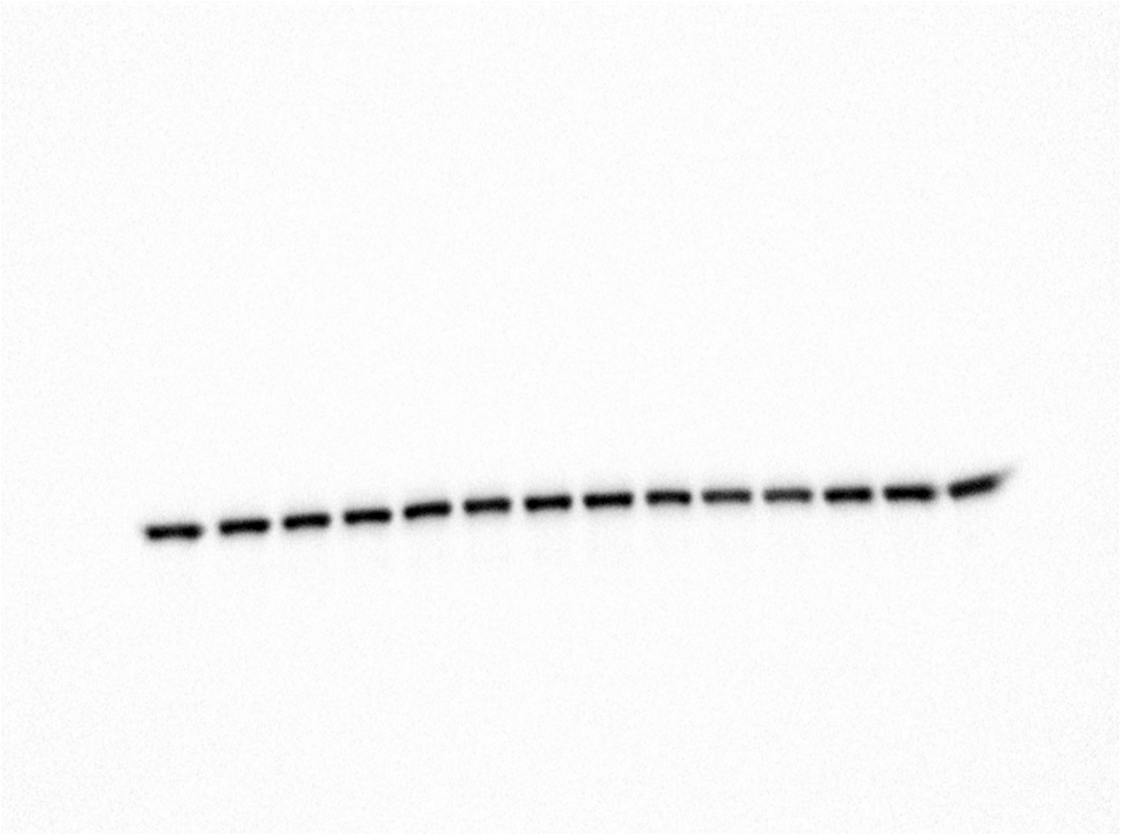

Figure S16b (right) aV5

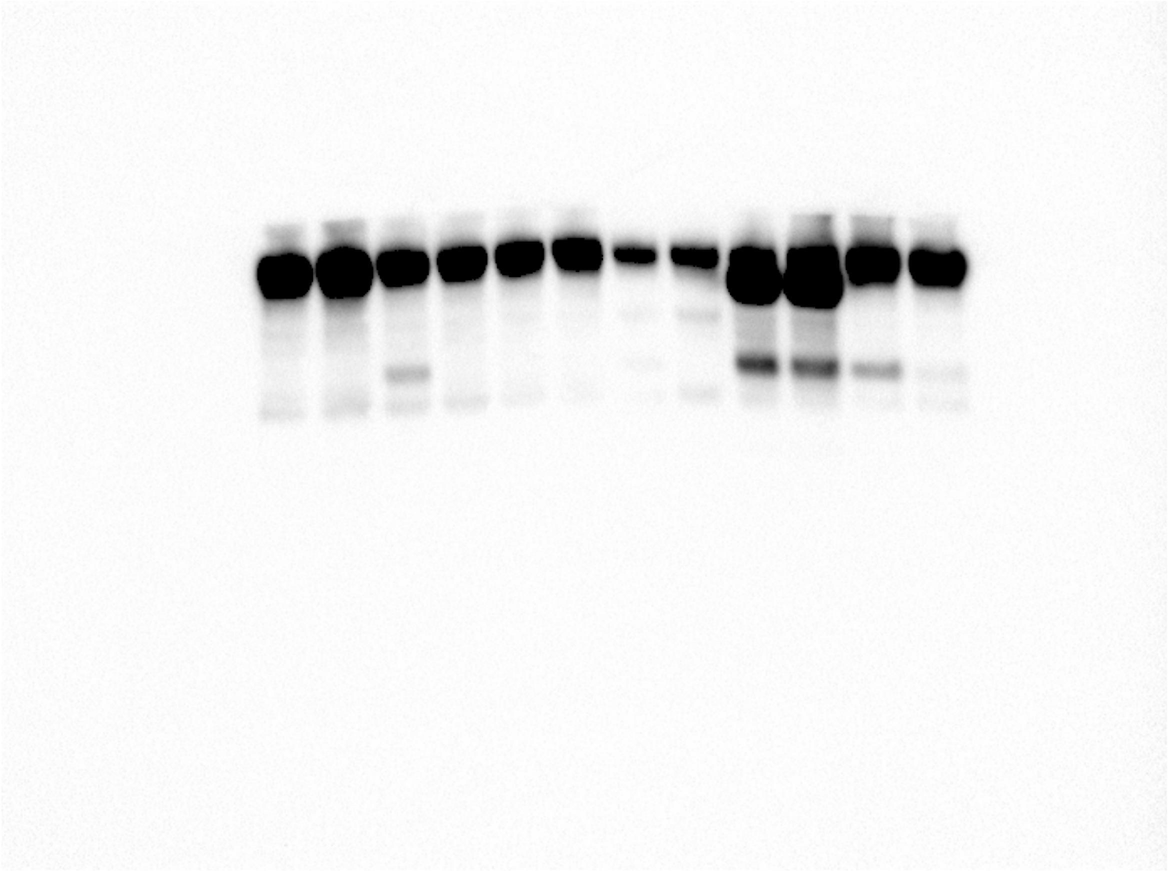

Figure S16b (right) aMyc

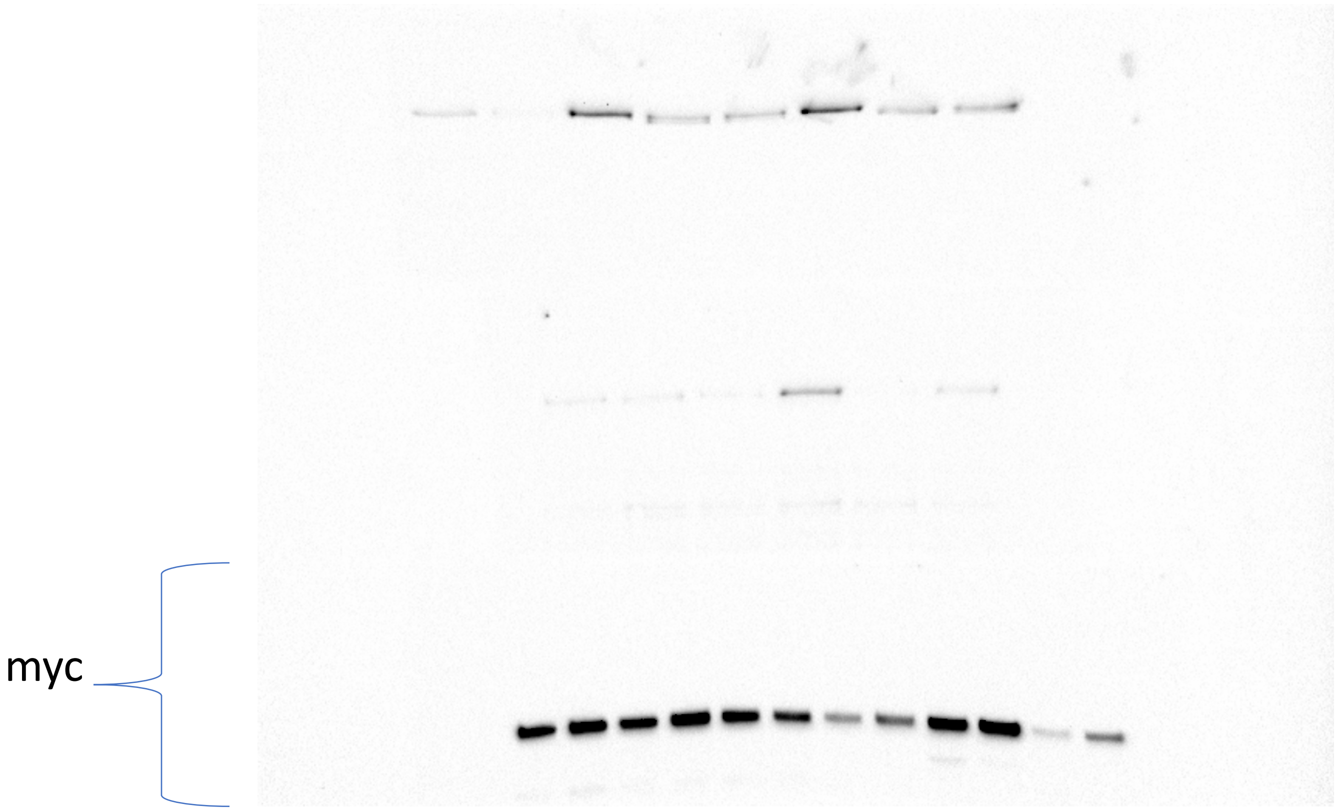

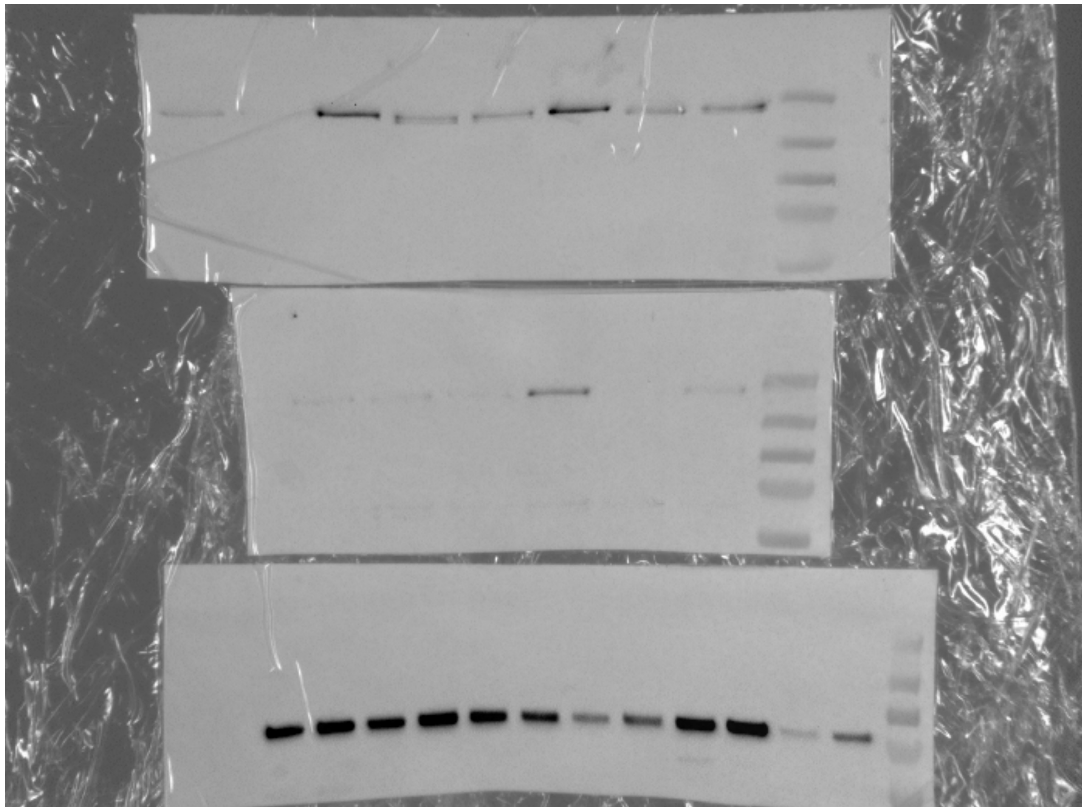

Figure S16b (right) aHA

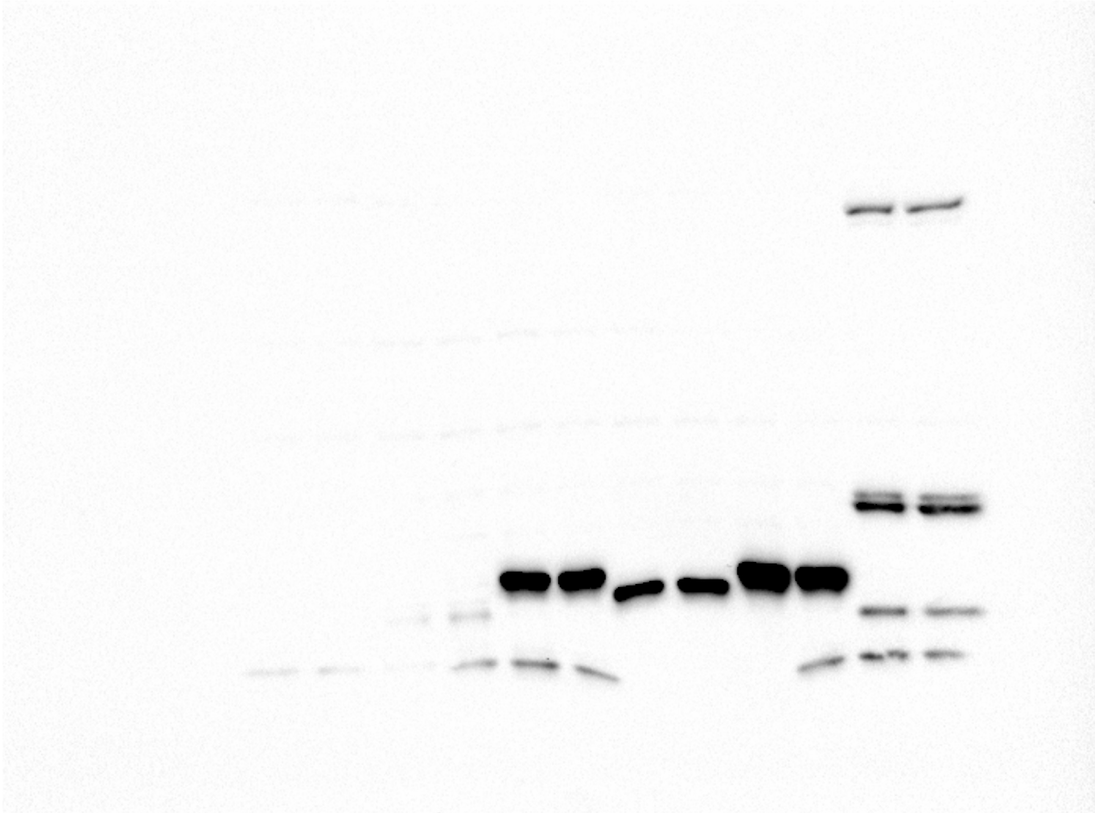

Figure S16b (right) aGAPDH

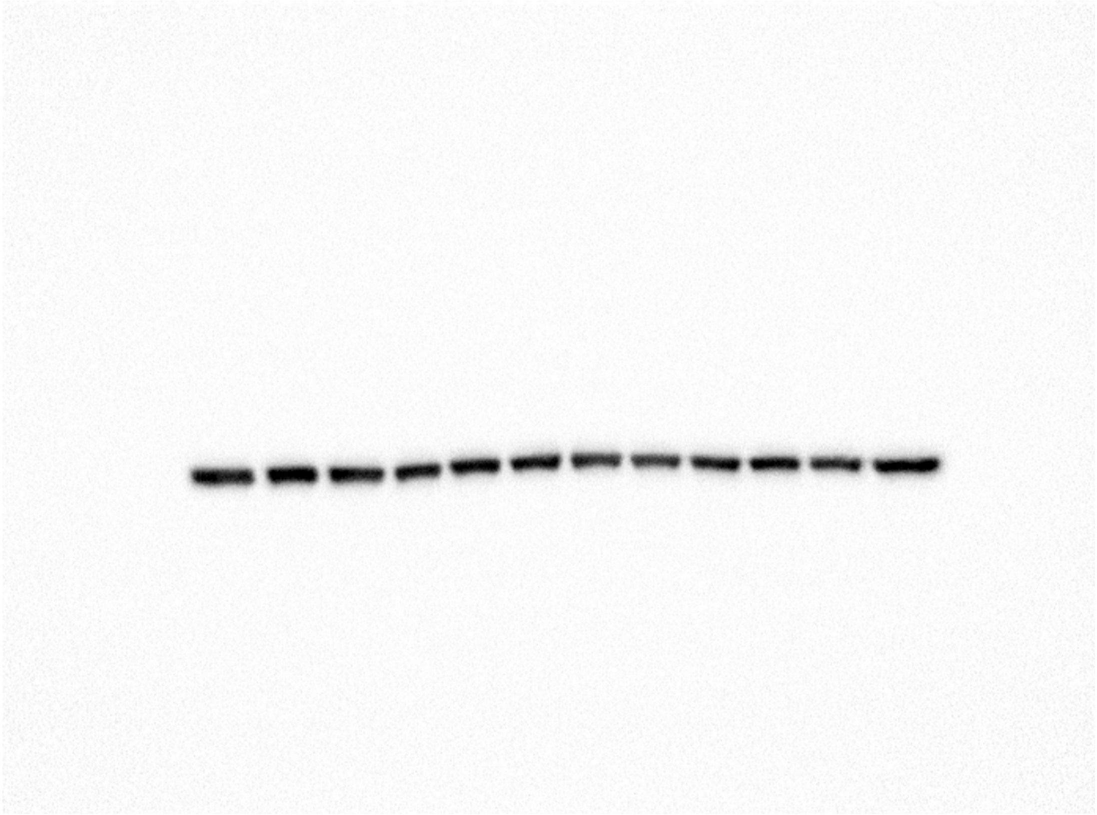

Supplement: S1 Raw Images — (PDF) [file pbio.3002144.s019.pdf]
